# Whole Genome Screening Procures a Holistic Hold of the Russian Chicken Gene Pool Heritage and Demographic History

**DOI:** 10.3390/biology12070979

**Published:** 2023-07-10

**Authors:** Michael N. Romanov, Alexandra S. Abdelmanova, Vladimir I. Fisinin, Elena A. Gladyr, Natalia A. Volkova, Dmitry V. Anshakov, Olga I. Stanishevskaya, Anatoly B. Vakhrameev, Arsen V. Dotsev, Darren K. Griffin, Natalia A. Zinovieva

**Affiliations:** 1 L. K. Ernst Federal Research Center for Animal Husbandry, Dubrovitsy, Podolsk 142132, Moscow Oblast, Russia; preevetic@mail.ru (A.S.A.); elenagladyr@mail.ru (E.A.G.); natavolkova@inbox.ru (N.A.V.); asnd@mail.ru (A.V.D.); 2 School of Biosciences, University of Kent, Canterbury CT2 7NJ, Kent, UK; d.k.griffin@kent.ac.uk; 3 Center “All-Russian Poultry Research and Technological Institute” of the Russian Academy of Sciences, Sergiev Posad 141311, Moscow Oblast, Russia; fisinin@vnitip.ru; 4 Breeding and Genetic Center “Zagorsk Experimental Breeding Farm”—Branch of the Federal Research Centre “All-Russian Poultry Research and Technological Institute” of the Russian Academy of Sciences, Sergiev Posad 141311, Moscow Oblast, Russia; a89265594669@rambler.ru; 5 Russian Research Institute of Farm Animal Genetics and Breeding—Branch of the L. K. Ernst Federal Research Center for Animal Husbandry, Pushkin, Saint Petersburg 196601, Russia; olgastan@list.ru (O.I.S.); ab_poultry@mail.ru (A.B.V.)

**Keywords:** chicken, Russian gene pool breeds, SNPs, whole genome screening, genetic diversity, phylogenetic relationships, demographic history

## Abstract

**Simple Summary:**

A collection of native farm animal breeds can be considered as a gene pool and a national heritage. Long-term artificial selection in domesticated animals has certain effects on their genomes, which can be investigated using genome-wide screens for DNA sequence variation, that is, so-called single nucleotide polymorphism (SNP) screens. Here, we looked at the genomes of 19 Russian chicken gene pool breeds, both native and imported, evaluating the contrasting egg, meat and dual-purpose types. Based on genetic diversity statistics, we identified differences between the breeds using many DNA markers (SNPs) that may represent genomic regions that are being selected for, either within a specific breed or shared between breeds. Our research will be helpful for further understanding the genomic diversity and demographic history of Russian domestic chickens. This would be essential for their successful breeding.

**Abstract:**

A study for genomic variation that may reflect putative selective signaling and be associated with economically important traits is instrumental for obtaining information about demographic and selection history in domestic animal species and populations. A rich variety of the Russian chicken gene pool breeds warrants a further detailed study. Specifically, their genomic features can derive implications from their genome architecture and selective footprints for their subsequent breeding and practical efficient exploitation. In the present work, whole genome genotyping of 19 chicken breeds (20 populations with up to 71 samples each) was performed using the Chicken 50 K BeadChip DNA chip. The studied breed sample included six native Russian breeds of chickens developed in the 17th–19th centuries, as well as eight Russian chicken breeds, including the Russian White (RW), created in the 20th century on the basis of improving local chickens using breeds of foreign selection. Five specialized foreign breeds of chickens, including the White Leghorn (WL), were used along with other breeds representing the Russian gene pool. The characteristics of the genetic diversity and phylogenetic relationships of the native breeds of chickens were represented in comparison with foreign breeds. It was established that the studied native breeds demonstrate their own genetic structure that distinguishes them from foreign breeds, and from each other. For example, we previously made an assumption on what could cause the differences between two RW populations, RW1 and RW2. From the data obtained here, it was verified that WL was additionally crossed to RW2, unlike RW1. Thus, inherently, RW1 is a purer population of this improved Russian breed. A significant contribution of the gene pool of native breeds to the global genetic diversity of chickens was shown. In general, based on the results of a multilateral survey of this sample of breeds, it can be concluded that phylogenetic relationships based on their genetic structure and variability robustly reflect the known, previously postulated and newly discovered patterns of evolution of native chickens. The results herein presented will aid selection and breeding work using this gene pool.

## 1. Introduction

The human-driven evolution of domesticated animals, including the chicken (*Gallus gallus* (Linnaeus, 1758); GGA; [1,2]), resulted in a wide variety of breeds. Historically, the dispersion of domestic animals on the planet, unlike wild species, has been driven by humans and correlated with migration and settlement of peoples, trade, military campaigns and other circumstances [3,4,5,6,7]. The formation of local (or indigenous) gene pools of farm animals has occurred, and continues to occur, under the influence of specific geographic, climatic, landscape, fodder, historical, economic and breeding conditions (e.g., [8,9,10,11,12,13,14,15]). Tixier-Boichard et al. [16] performed a component analysis of the factors influencing the topography of the location of numerous chicken populations of the global gene pool in the space of multidimensional scaling. Therewith, it turned out that the degree of selection pressure and the purpose of use of chicken breeds, i.e., factors associated with human activity, made the greatest contribution to interpopulation variability. As reviewed by Moiseyeva et al. [3], when dividing chicken breeds by origin into European and Asian categories (e.g., [17]), European breeds have a lower diversity (heterozygosity) in terms of morphological, biochemical and molecular markers. A lower genetic variability of European egg-type chickens as compared to meat-type breeds of Asian roots has been noted in several studies (e.g., [18,19,20,21]). Moiseyeva et al. [3] suggested two possible reasons for this phenomenon. Firstly, the Asian region is characterized by a higher diversity of climatic, landscape and animal fodder factors. Secondly, it is inhabited by more numerous ethnic groups with an ancient and diverse culture and, consequently, unequal interests and needs in the selection of chickens and other species of domestic animals as compared to the European territory. It is no coincidence that it is in Asia that most of the centers of domestication of wild animals and origins of cultivated plants are located (e.g., [3,22,23]).

Poultry production is an important sector of agriculture that supplies valuable foodstuffs, such as eggs and meat [24,25,26]. In the context of sustainable food security in individual countries, available poultry genetic resources, their genetic potential assessment as well as their conservation and practical use by introducing highly effective biotechnologies are of an increasing importance [27,28,29,30,31]. Currently, breeds of the Russian chicken gene pool heritage that have been divergently selected for contrasting performance and other phenotypic traits are maintained at the two research institutions, All-Russian Poultry Research and Technological Institute (ARPRTI), Sergiev Posad, Moscow Oblast [32] and Russian Research Institute of Farm Animal Genetics and Breeding (RRIFAGB), Pushkin, St. Petersburg [33]. Some of these breeds, e.g., Orloff Mille Fleur (OMF; [32,33]), Yurlov Crower (YC; [32,33,34,35,36]), Ushanka (Ush; [32,33,37]) and Poltava Clay (PC; [32,33,38,39,40,41]), were created ~150–200 years ago or even earlier in the conditions of Russian local farms. Previously, we successfully implemented the whole genome screen technology to evaluate selection footprints and key candidate genes for egg and meat performance, adaptation and other phenotypic traits in a few Russian chicken gene pool breeds [37,42]. Further genome-wide examination of many other chicken breeds in Russia is urgently needed and anticipated to reveal more unique features of their genetic blueprint useful for creating reference populations subject to subsequent genomic prediction and selection.

In this regard, the present investigation was aimed at comparing the genomic architecture of the Russian chicken gene pool, which includes breeds of different utility and demographic history. In the course of this study, we performed genome-wide genotyping of various chicken breeds (populations) using the Chicken 50 K BeadChip DNA chip. A comparative assessment of the biodiversity of the studied breeds was carried out using indices of observed and expected heterozygosity, allelic diversity, inbreeding coefficient *F*_IS_, etc. The effective population size (*N*_e_) at the present time and its change over 50–500 generations were explored. Genetic distances were calculated, and genetic relationships between breeds were established, with the genetic structure of the studied breeds being assessed. The obtained data of the single nucleotide polymorphism (SNP)-based analysis were considered in the aspect of the demographic history of the breeds and directions of selection and breeding work with the breeds.

## 2. Materials and Methods

### 2.1. Breeds, Sampling and Ethics Statement

To study the genomic architecture of the Russian chicken gene pool, we surveyed a total of 19 breeds (20 populations). These embraced the following six indigenous breeds developed in the 17th–19th centuries: OMF, PC, Russian Black Bearded (RBB), Russian Crested (RC), Ush and YC, in the creation of which imported breeds could also be used. There were also eight native chicken breeds developed in the 20th century based on the improvement of local breeds by crossing them to breeds of foreign origin and selection as follows: Adler Silver (AS), Kotlyarevsky (Kt), Kuchino Jubilee (KJ), Leningrad Mille Fleur (LMF), Pervomai (Pm), Russian White (RW, of two populations, RW1 and RW2 kept at ARPRTI and RRIFAGB, respectively), Ushanka Foot-feathered (UshF) and Zagorsk Salmon (ZS). Along with the above breeds representing the Russian gene pool, the following five specialized foreign breeds of chickens were used: Australorp Black (AoB), Cornish White (CW), New Hampshire (NH), Rhode Island Red (RIR) and White Leghorn (WL). In terms of utility type, RW and WL belonged to the egg-type breeds (ETBs) and CW to the meat-type breeds (MTBs), with all others being dual-purpose breeds (DPBs), of meat-egg (MEB; with meat traits being more targeted by selection) or egg-meat (EMB; with egg performance traits being predominantly selected) subtypes [33]. A detailed description of the studied breeds is given in Table 1. These involved a number of famous breeds created in pre-revolutionary Russia and differing in their unique phenotypes and features, e.g., OMF (descended from game breed (GB) chickens, among others; [43]), YC [34,35] and PC [38,39,40]. In addition, there were breeds that have been subjected to long-term breeding for egg and/or meat productivity, e.g., the specialized breeds RW and CW [42].

Birds of the Ush, OMF and CW breeds were purchased from the Breeding and Genetic Center “Zagorsk Experimental Breeding Farm”—Branch of the Federal Research Centre “ARPRTI” and housed in the bioresource Gene Pool Collection of Farm and Wild Animals and Birds at the L. K. Ernst Federal Research Centre for Animal Husbandry (LKEFRCAH). Samples of other breeds were obtained from the ARPRTI farm. The RRIFAGB supplied samples of the RW breed, and the Institute of Farm Animal Genetics/Friedrich-Loeffler-Institut (IFAG/FLI) those of the WL line G11. A total of 528 individual samples (up to 71 per population) were used in this study, a majority of them being feather pulp samples and fewer blood or DNA (G11) samples. In order to minimize any potential bird discomfort or distress, feather and blood samples were collected by trained lab staff as adherent to the LKEFRCAH and IFAG/FLI ethical guidelines.

### 2.2. DNA Isolation

The manufacturer’s recommendations were followed when extracting DNA using Nexttec columns (Nexttec Biotechnologie GmbH, Leverkusen, Germany). We employed a Qubit 3.0 fluorometer to define the concentration of dsDNA solutions (Thermo Fisher Scientific, Wilmington, DE, USA). Values of OD260/280 ratio were measured using a NanoDrop-2000 to determine the recovered DNA purity (Thermo Fisher Scientific).

### 2.3. SNP Markers and Genotyping Quality Control

Using a Chicken 50 K CobbCons SNP microarray (Illumina, San Diego, CA, USA), individual sample genotyping was performed. Before applying any quality control filters, 53,872 SNP markers were available. Using PLINK 1.9 software [50], the following filters were set up to adjust the quality of the SNP genotypes: at least 90% of loci (--geno 0.1) successfully genotyped in at least 80% of samples (--mind 0.2), with minor alleles being contained at least 5% of the time (--maf 0.05) and the linkage disequilibrium (LD) criterion being greater than 50% (--indep-pairwise 50 5 0.5). A total of 39,778 SNPs on 28 autosomes (GGA1 to GGA28) were selected after filtering for further examination. The R software (version 4.2.3) environment was used to create the input files for the analysis and subsequent visualization of the findings [51].

### 2.4. Genetic Diversity, Population Structure and Split and Admixture

The observed heterozygosity (*H_O_*), expected heterozygosity (*H_E_*), unbiased expected heterozygosity (*_U_H_E_*) [52], rarefied allelic richness (*A_R_*) [53] and *_U_H_E_*-based inbreeding coefficient (*_U_F*_IS_) were computed in the R package diveRsity [54] to measure genetic diversity of the breeds studied. We also carried out the analysis of molecular variance (AMOVA) for our dataset using the following hierarchical structure: breed group (old Russian, improved Russian and specialized foreign) > breed > sample. This analysis was performed using the R package poppr [55]. Using PLINK 1.9, genetic differences between the examined breeds were determined. The R package ggplot2 was used to conduct the principal component analysis (PCA) visualization [56]. Additionally, PCA plots as well as hierarchical clustering trees using Euclidean distances were generated using the Phantasus web application [57].

The SplitsTree 4.14.5 software [58] and T-REX web server [59] were employed to plot Neighbor-Net and Neighbor-Joining [60] dendrograms using a matrix of pairwise *F*_ST_ [61] and Reynolds et al. [62] genetic distances. The former distance was the respective statistic also known as the fixation index. The latter distance was calculated based on the appropriate Reynolds et al. [62] algorithm that assumed that genetic differentiation occurs only through genetic drift and without mutations. In addition, the Neighbor-Joining trees were reconstructed using the ADDTREE [63] and Unweighted Neighbor-Joining [64] methods. Moreover, heat maps and trees were generated with the ClustVis web tool [65] using Euclidean distances for both rows and columns of the matrix (with the *average* option selected for the clustering method).

Using the Admixture v1.3 program [66], model-based clustering (obtained by Bayesian clustering) was completed to refine population structure. The cross-validation (CV) procedure was used to compute numbers (K) of clusters (ancestral populations), with K values ranging from 1 to 30 and its lowest CV error being the optimal number of clusters. The findings of the admixture analysis were visualized using the R package BITE [67].

### 2.5. Runs of Homozygosity Analysis

For the estimation of ROHs, the consecutive runs technique [68] implemented in the R package detectRUNS (version 0.9.6) [69] was used, which is a window-free method for consecutive SNP-based detection. In order to prevent underestimating the number of ROHs longer than 8 Mb, we permitted one SNP with an unknown genotype and up to one potential heterozygous genotype in one run [70]. We set the minimum length of an ROH at 500 kb to account for strong linkage disequilibrium (LD), which usually extends up to about 100 kb [71], and to exclude short and very frequent ROHs. We determined the minimum number of SNPs (*l*), which was first determined by Lencz et al. [72] and subsequently modified by Purfield et al. [73], in order to reduce false-positive results:

$$l=\frac{log_{e}\frac{\alpha }{n_{s}\cdot n_{i}}}{log_{e}\left(1-\bar{het}\right)},$$

where *n_s_* is the average number of SNPs that each individual has been genotyped for, *n_i_* is the total number of individuals that have been genotyped, *α* is percentage of false-positive ROHs (equal to 0.05 as the study’s cutoff) and $\bar{het}$ is the average level of heterozygosity across all SNPs. In our situation, a minimum of 23 SNPs were required to be considered.

Each individual’s ROH length and number were calculated, and these values were then averaged across all individuals in each breed. Additionally, we calculated the genomic inbreeding coefficient based on ROH (*F*_ROH_), which is the ratio of the total autosomal SNP coverage to the aggregate of all ROHs for each animal (0.94 Gb).

As indicated by other studies [74,75], putative ROH islands were defined as overlapping homozygous regions shared by more than 50% of analyzed individuals within each breed. Given that shorter segments of 0.3–1 Mb are more common in the genome of white egg-laying hens [76], we set the minimum overlapping length size threshold at 0.3 Mb.

### 2.6. Demographic History Inference

The degree and patterns of population divergence (splits) and the level of gene flow between the studied breeds were inferred using the TreeMix 1.12 program [77]. We tested 0 to 5 migration events (edges) with 30 iterations for each event. The optimal number of migration edges was determined using the R package *OptM* [78] and TreeMix output files. The common quail (*Coturnix coturnix* (Linnaeus, 1758)), genotyped using the same chicken microarray, was used as an outgroup in reconstructing the maximum likelihood (ML) trees based on ~16K validated SNPs. The respective residual matrices were visualized as heat maps.

Using the algorithm built into the *SNeP* v.1.1 program [79] and based on LD [79,80,81,82], *N*_e_ was calculated over the previous 50 to 500 generations. Except for the recombination rate modifier computed following Sved and Feldman [83], the default settings were used. In order to study the rate and direction of *N*_e_ changes that occurred over 50 generations, an analysis of the *N*_e_ slope (*N*_e*S*_) was also performed [84]. Using the median of 50 most recent *N*_e_ values, the slope of each segment connecting pairs of adjacent *N*_e_ values was defined and the results were normalized. This enabled us to determine the *N*_e_ change coefficient for the increase of pairwise coancestry (*N*_e*C*_) [85] in order to estimate the amount of *N*_e_ change over the previous 50 generations. Using the *SNeP* program [79], it was also possible to calculate the mean *r*^2^ (LD) and standard deviation *r*^2^ in a bin, the mean distance (*dist*) between each pair of SNP markers in a cell and the number of SNP markers (*items*) used to calculate *r*^2^ in the bin.

Statistical data analysis, including calculation of means, mean standard errors and Student’s *t*-statistics, was performed using Excel (Microsoft Corporation, Redmond, WA, USA) and the appropriate web tools [86,87].

## 3. Results

### 3.1. Genetic Diversity

As follows from the genetic diversity data presented in Table 2, the *H_O_* values varied in the studied breeds from 0.164 ± 0.001 in WL to 0.365 ± 0.001 in CW. Interestingly, the same pattern, i.e., the minimum values for WL and the maximum ones for CW, persisted for other indices of genetic diversity. In particular, in terms of *H_E_*, *_U_H_E_* and *A_R_*, WL had the lowest values (0.185 ± 0.001, 0.185 ± 0.001 and 1.452 ± 0.002, respectively), and the largest ones were found in CW (0.368 ± 0.001, 0.371 ± 0.001 and 1.876 ± 0.001, respectively; *p* < 0.001). The rest of the breeds had intermediate diversity indicator values. Collectively, we can see that the studied commercial WL line, purposefully bred for egg performance, has the characteristic least genetic variability. Native and foreign DPBs (of the EMB and MEB subtypes of utility) had a significantly greater genetic variation. Another specialized ETB of RW resulted from crossing local chickens with that WL fell into the same intermediate diversity group. Finally, the maximum variability was observed in CW, a commercial MTB synthetic by origin, derived from the Asiatic GBs and MTBs of Asil, White Malay, Indian Game and Cochin, and subjected to selection for meat production traits contrastingly different from the ETB of WL. One indigenous MEB, YC, was also close to CW in terms of variability (Table 2), probably due to the fact that at least five different breeds, including the Asiatic MTBs of Brahma, Cochin and Langshan, are believed to have been used for its creation. In addition, there was a trend of higher average diversity indices (i.e., heterozygosities and allelic richness) in the old Russian indigenous breeds, intermediate ones in the group of improved Russian breeds and lower ones among the specialized foreign breeds (Table 2). We also calculated the diversity indices for the three breed groups (i.e., old Russian, improved Russian and specialized foreign) obtained by pooling individual genotypes within each breed group (Supplementary Table S1). As a result, the old Russian breed group had greater mean values of diversity indicators, with intermediate and lower values being in the improved Russian and specialized foreign breed groups, respectively.

The results of the AMOVA-based analysis for the three breed groups (i.e., old Russian, improved Russian and specialized foreign) are presented in Table 3. The main share of variation conformed to the differences between individuals within the populations (83.71%, *p*-value = 0.001). Variation between the breed groups was only 1.36% (*p*-value = 0.011). The results obtained were convincing since some breeds, including those belonging to different groups, were of mixed origin. A comparable pattern was observed in a study of Chinese gamecocks and native chicken breeds [88]. However, a relatively high proportion of variation between breeds within groups (14.93%, *p*-value = 0.001; Table 3) may be indicative of the apartness of each breed as a result of selection work.

When completing a comprehensive PCA of the genetic variability within this breed sample using the four diversity indicators *H_O_*, *H_E_*, *_U_H_E_* and *A_R_*, we were able to distinguish four breed clusters (Supplementary Figure S1A). The first one was the farthest from the rest of the clusters, located in the lower right corner of the PCA plot and formed by the single ETB of WL that had a pronounced minimum variability. The second cluster was situated in the left extreme position on the graph and included two breeds with the greatest variation, the MTB of CW and the MEB of YC. The other two clusters were located in the central part of the PCA plot and mainly consisted of DPBs. Herewith, the third cluster was composed of breeds with higher values of variability indicators, and the fourth cluster (which also involved the ETB of RW) was represented with breeds that had a lower variation. A similar distribution of the studied breeds based on the same four diversity indices was also observed using the hierarchical clustering procedure (Supplementary Figure S1B).

We also evaluated the diversity of genotyped breeds depending on inbreeding coefficients that characterized the genetic structure of populations from a little different angle (Table 2). Negative *_U_F*_IS_ values indicated a slight excess of heterozygotes in OMF, PC, LMF and NH (−0.001 to −0.067), while other breeds showed no or a slight deficiency of heterozygotes (0.000 to 0.103) than what would be expected under the Hardy–Weinberg equilibrium (Table 2). Therefore, we observed a rather significant scatter in the number of heterozygotes in the studied breeds as tested for a deviation from the Hardy–Weinberg equilibrium. Notably, WL had not only the minimum indicators of genetic variability, but also the highest values of both the generally accepted inbreeding coefficient (*F*_IS_ = 0.080) and its unbiased derivative, *_U_F*_IS_ (0.103). This was indicative of some shifts in the population structure of this selected WL line towards an excessive number of homozygotes. Judging by the *F*_IS_ coefficient, many other breeds, in contrast, showed some redundancy of heterozygotes, except for RC, Ush, YC, AS, AoB and CW. On the other hand, based on the *_U_F*_IS_ values, the population structure of some old indigenous (RBB, RC, Ush and YC), improved native (UshF, ZS, RW, Pm, Kt and AS) and specialized foreign (CW, RIR and AoB) breeds tended toward excessive homozygotes.

The distribution of genotyped breeds based on the inbreeding coefficients *F*_IS_ and *_U_F*_IS_ and using PCA and hierarchical clustering is shown in Supplementary Figure S2A,B, respectively. On both of these graphs, five major clusters can be distinguished. WL that had the maximum *F*_IS_ and *_U_F*_IS_ values formed one well-isolated single cluster. Kt was located separately from other breeds as another single cluster. OMF that had an excess of heterozygotes also formed a separate cluster. One more cluster was composed of five breeds that also demonstrated a certain excessive number of heterozygotes. The largest cluster (No. 3) can be divided into two subclusters: in one (3a), there was some bias towards an excess of heterozygotes, and in the other (3b), a redundancy of homozygotes (Supplementary Figure S2A).

### 3.2. ROH Distribution in Chicken Breed Genomes

According to Table 4, we revealed the lowest mean number of ROH segments in the YC genome (70.11) and the greatest one in WL (249.75). Furthermore, YC and CW chickens had the lowest coverage of genome by ROHs (146.91 and 154.61 Mb, respectively), whereas WL had the greatest total length of ROHs (525.64 Mb). This resulted in the lowest values of inbreeding coefficient calculated based on ROHs in YC and CW (*F*_ROH_ = 0.154 and 0.162, respectively), with the highest value being identified in WL (0.551). There was an overall tendency of lower average ROH-related metrics in the genomes of old Russian indigenous chickens, intermediate ones in the improved Russian breeds and higher values in the specialized imported breeds (Table 4). These observations were highly concordant with the above genetic diversity data for the same chicken breeds studied (Table 2). When pooling individual genotypes to form the respective three breed groups, the old Russian breeds had significantly lower ROH-based statistics than those in the improved Russian breeds (Supplementary Table S1). The respective Manhattan plots of the distribution of ROH islands in individual chicken breeds can be seen in Supplementary Figure S3.

### 3.3. Breed Relationship and Admixture

**Figure 1:**
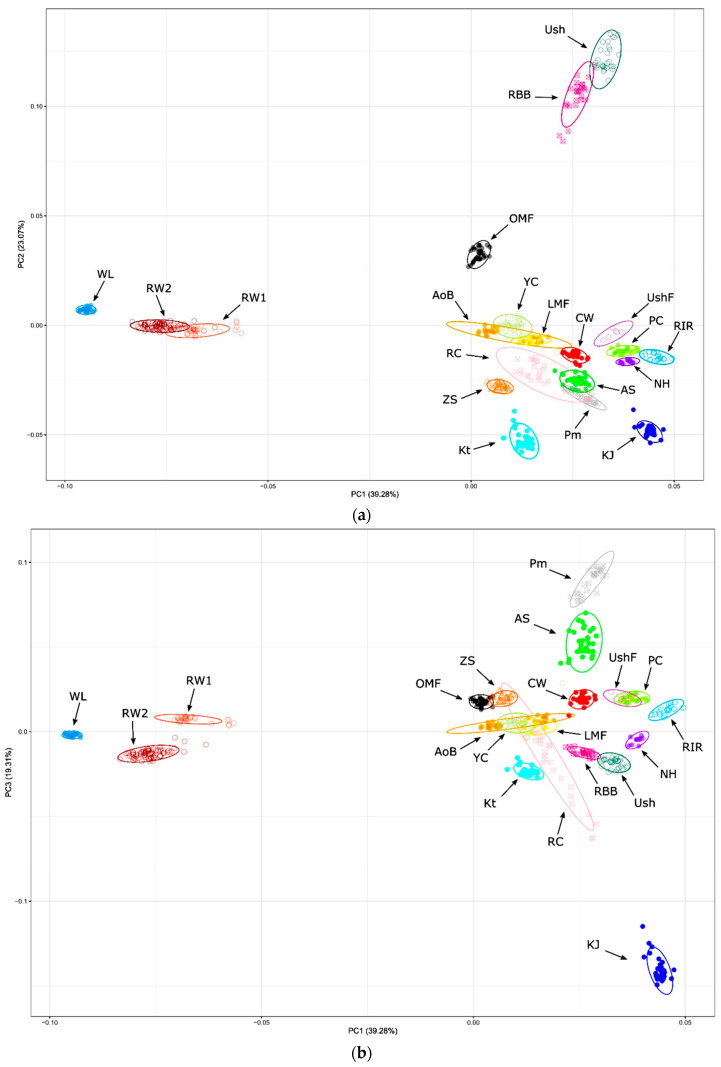
PCA plots for the studied chicken breeds (populations) based on genome-wide SNP genotypes. Distribution of breeds and individuals is shown in the dimensions of three coordinates, i.e., the first (PC1; *x*-axis; (**a**,**b**)), second (PC2; *y*-axis; (**a**)) and third (PC3; *y*-axis; (**b**)) principal components (PCs), with the respective percentage of the total variance (within the parentheses), which can be explained by each of the two PCs. Breed codes are given in Table 1.

As one of the approaches to assessing the genetic structure and differentiation of the studied breeds based on SNP genotyping, the PCA method was used. The results of the corresponding clustering of genotyped individuals and breeds are shown in Figure 1 and Supplementary Figure S4. The PCA plots in Figure 1 demonstrated a high degree of genetic identification of genotyped individuals, i.e., there was a fairly good match of a single individual to the breed cluster to which this individual belonged. This proved, firstly, a high resolution power of genome-wide genotyping of this sample of breeds using a medium-density SNP chip. Secondly, there was a definite consolidation of the genetic structure of most breeds. Remarkably, both the PC1–PC2 (Figure 1a) and PC1–PC3 (Figure 1b) plots well reflected the genetic differentiation of breeds and explained about the same amount of total variance, pointing out their fairly significant and adequate display of clustering patterns. As can be seen on these PCA plots (Figure 1a,b), certain breeds were characterized by a rather pronounced genetic uniqueness. These included a distinctive ETB cluster of compactly displayed WL individuals and two closer, but still differentiated, populations RW1 and RW2. Such old indigenous breeds as Ush and RBB, as well as OMF, were also well distinguished from the other breeds, with Ush and RBB showing a great genetic similarity relative to each other (Figure 1a). An improved native breed of KJ appeared to have some unique genetic features, too (Figure 1b), whereas the other, mostly synthetic by origin, DPBs formed a conglomeration of breeds situated quite close to each other (Figure 1a). The latter embraced clusters of individuals of the breeds that were more similar in genetic structure to each other and were located more crowded in the bottom right (Figure 1a) and upper right (Figure 1b) parts of the two graphs. Within this conglomeration, we observed a closer vicinity of RIR, NH (derived from RIR) and PC (presumably intercrossed with RIR and NH; Figure 1a). Closer to each other were also Pm and AS (originated from Pm).

**Figure 2:**
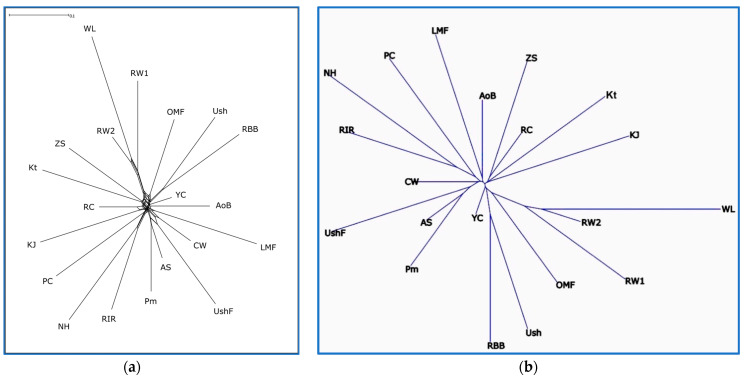
Phylogenetic relationships established between the studied chicken breeds (populations) based on the *F*_ST_ genetic distances. Unrooted radial trees were reconstructed using the Neighbor-Net algorithm [58] (**a**) and the Neighbor-Joining/ADDTREE method [63] (**b**) Breed codes are given in Table 1.

The pairwise interbreed genetic distances that were computed using the *F*_ST_ statistic and the Reynolds et al. [62] algorithm are presented in Supplementary Tables S2 and S3, respectively. Using these obtained distances for the full spectrum of breeds genotyped here, the respective phylogenetic trees were built as shown in Figure 2 and Supplementary Figures S5 and S6. According to the similar tree topology presented in these figures, we identified the presence of between-breed phylogenetic relationships, e.g., for such pairs/trios of breeds as (1) the ETBs of WL and RW, (2) the MEBs of Ush and RBB, (3) the EMBs of RIR, NH and PC, (4) the MEBs of Pm and AS, (5) the MEBs of ZS and Kt and (6) the DPBs of AoB and LMF.

**Figure 3:**
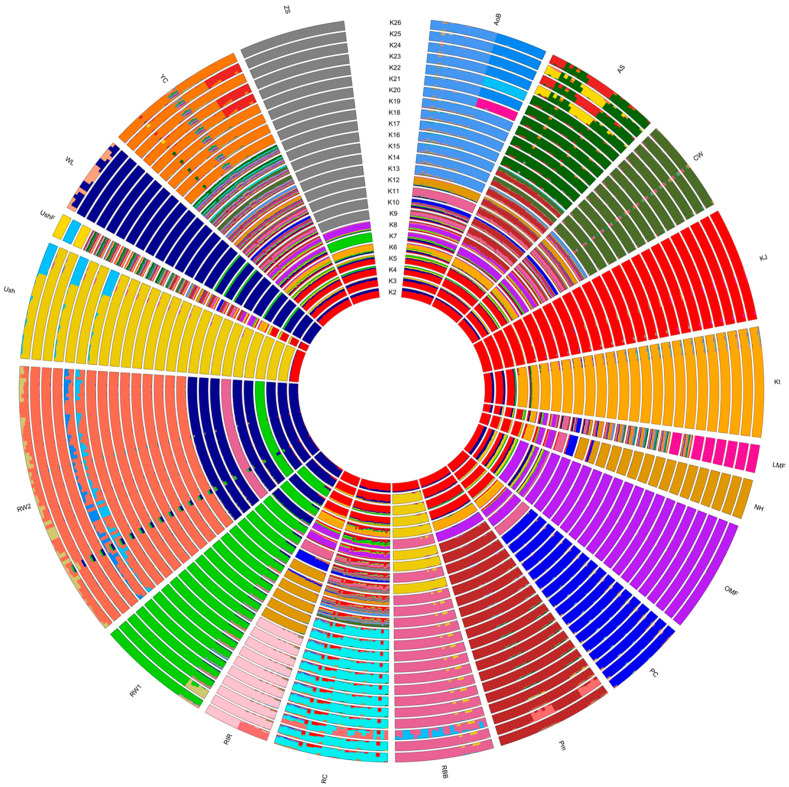
Admixture circular diagram plotted in the R package BITE [67] and representing cluster structure and individual ancestry proportions in the studied populations at K = 2 to 26. Breed codes are given in Table 1.

In addition, we performed an admixture-based genetic structure analysis, determined the mixing degree in the composition of the given set of individuals and populations (breeds) based on the SNP genotype information and estimated the origin of populations from K hypothetical ancestral populations. As followed from the procedure for calculating CV errors for different K values, the minimum error value corresponded to K = 26 (Supplementary Figure S7). When discriminating the populations using admixture, the corresponding mixing patterns of breed composition were observed (Figure 3, Supplementary Figures S8 and S9). The value of K = 2 appeared to conform to the two main evolutionary lineages of domestic chickens, of the Mediterranean (ETB) and Asiatic (MTB) roots [89]. WL, a typical breed of Mediterranean origin, had a solid dark blue bar coloration on the admixture plot at K = 2. Two ETB populations of RW had a significant proportion of WL genotypes in their genomes, with their respective bars being largely dark blue on this plot. In the genomes of other breeds, the red Asiatic component dominated, while dark blue Mediterranean genotype variants were present to a much lesser extent, as was also reflected on the admixture plot. At K = 3, one more ancient evolutionary Asiatic lineage of GB (of yellow bar color) may have been added, which participated to some extent in the formation of many later breeds [89]. At K = 4, another ETB sublineage (of green bar color) emerged. Accordingly, WL apparently began to break up into two sublineages, whereas RW1 was mostly composed of the second ETB sublineage variants, which could, to some extent, affect further steps in the identification of ancestral populations. The optimal patterns of the breed-specific genetic structure were found at K = 26. On the corresponding bar plot, the unique genomic composition of each breed can be seen (Figure 3, Supplementary Figures S8 and S9), suggesting the distinctive patterns of breed consolidation and stratification in accordance with their origin and breeding history. Although the situation when K is more than the number of populations may seem unusual, such a pattern can be observed when the samples of various breeds studied are of mixed origin. Accordingly, the number of clusters K equal to, or lower than, the number of populations was not enough in our case to differentiate samples between breeds clearly. With an increase in the number K, clusters began forming within breeds, uniting samples not by breed, but by other factors, e.g., belonging to a line or a family.

### 3.4. Demographic History Inference

#### 3.4.1. Population Divergence and Gene Flow

**Figure 4:**
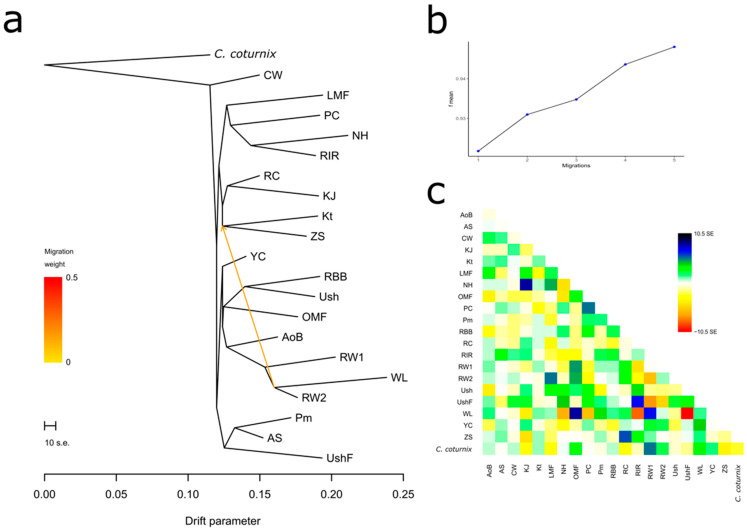
Example of the assessed degree of divergence and the level of gene flow between the studied breeds using 25 iterations. (**a**) Rooted maximum likelihood tree with one migration event. Common quail (*C. coturnix*) was used as a root; cut length 10 s.e. corresponds to ten times the average standard error (s.e.) estimated from the sample covariance matrix. Estimated gene flow is shown by an arrow pointing from a donor population (ancestor of WL and RW2) to a recipient one (ancestor of Kt and ZS) and is colored red in proportion to the intensity of the gene flow. (**b**) Plot representing the proportion of variance (f-index) in the sample covariance matrix (¶W) accounted for by the model covariance matrix (W) as a function of the number of migration events. (**c**) Residual matrix derived from the TreeMix analysis for a single migration event, expressed as the number of standard error deviations for the observations in the respective breeds.

One of the demographic history inference aspects explored here was the assessment of breed/population divergence (or split) and gene flow (or migration events) [77]. The respective analysis results are presented in Figure 4 and Supplementary Figure S10A–D. As exemplified in Figure 4a, there was a migration event from the common ancestor of RW and WL to the ancestor of ZS and Kt. This does not contradict the history of breed origin: RW, indeed, was used in developing ZS and Kt, and this explains the discovered gene flow example. We also discovered several other gene flow events that conformed to the known origin and breeding history of the breeds (Supplementary Figure S10A). In addition, maximum likelihood trees in Figure 4a and Supplementary Figure S10A,B reflected the plausible breed/population divergence that was largely in agreement with the PCA plots (Figure 1) and phylogenetic dendrograms (Figure 2, Supplementary Figures S5 and S6). However, the maximum likelihood tree reconstruction algorithm was more sensitive in placing the MTB of CW as a single basal offshoot not related directly to any other breed.

#### 3.4.2. Effective Population Size

**Figure 5:**
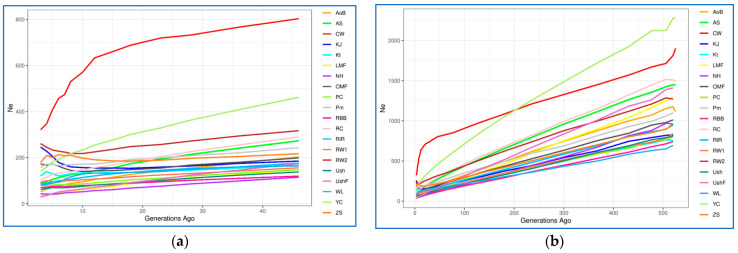

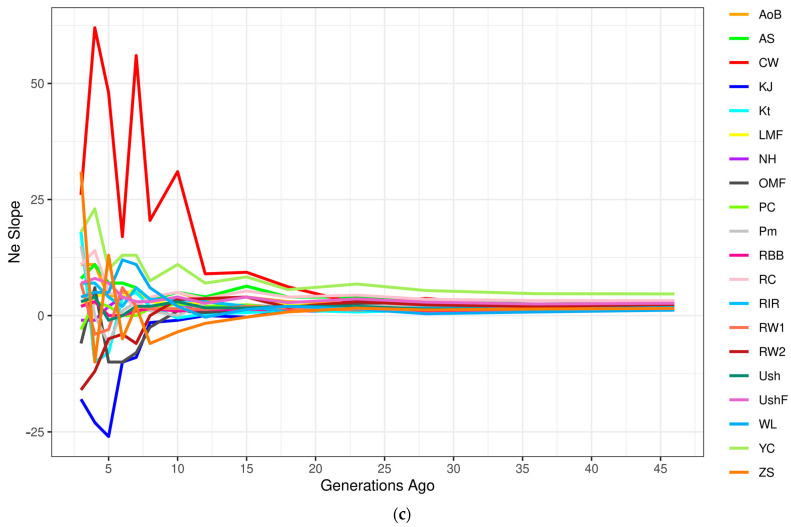
Dynamics of effective population size (*N*_e_) changes in the studied chicken breeds (populations). Absolute *N*_e_ change across generations from approximately 50 (**a**) and 500 (**b**) generations ago; (**c**) *N*_e_ slope (*N*_e*S*_), or relative *N*_e_ change across generations from approximately 50 generations ago: the continuous rate of change is shown as a horizontal straight line at point 0 on the *y*-axis, while deviations above and below zero represent, respectively, the relative increase and decrease in the changing *N*_e*S*_ as compared to the previous generation. Breed codes are given in Table 1.

The assessment results of demographic history in terms of *N*_e_ values at the present time and their change over recent 50–500 generations among the studied breeds are presented in Figure 5, Supplementary Table S4 and Supplementary Figures S11 and S12. It is known that *N*_e_ is an important indicator that describes the demographic history of a particular population. Consideration of this indicator also allows us to assume how many individuals could participate in the formation of the breed [90]. Based on the genome-wide analysis, CW had the largest *N*_e_ value three generations ago (*N*_e_ = 321; Supplementary Table S4; Figure 5a). The remaining breeds (populations) formed four large groups of breeds: (1) KJ and RW2, *N*_e_ = [246; 261]; (2) Kt, YC, Pm, OMF and ZS, *N*_e_ = [121; 179]; (3) WL, PC, RIR, AS, RW1 and RC, *N*_e_ = [80; 103]; and (4) UshF, LMF, NH, AoB, Ush and RBB, *N*_e_ = [29; 70] (Supplementary Table S4; Figure 5a). This pattern of *N*_e_ values has been observed over the last 10–20 generations; the exceptions were OMF, ZS, KJ and RW2 that previously had somewhat reduced *N*_e_ values. When considering the state of the studied populations 46 generations ago (Supplementary Table S4; Figure 5a), most breeds had *N*_e_ values ranging from 115 to 203, three breeds (ZS, Pm, AS, RC) were characterized by higher *N*_e_ values (217–290) and three more breeds (RW2, YC and CW) significantly outperformed the others by this indicator (317–803). If we look at an even earlier demographic history of populations (at the level of 500–525 generations ago, or approximately 230–240 years ago (Supplementary Table S4, Figure 5b), WL had the smallest *N*_e_ (694), while CW and YC had the largest *N*_e_ (1814 and 2279, respectively). Remarkably, there was a sharp *N*_e_ reduction in YC (from 2279 to 141), suggesting that this breed went through a severe genetic bottleneck, as confirmed by the fact that YC was almost extinct by the end of World War II. It can be assumed that breeds with a low *N*_e_, both at present and 50–500 generations ago, apparently descended from a limited number of ancestors or were under significant selection pressure in a number of generations. However, it should be noted that, as a rule, a majority of the studied breeds were developed by hybridization of several breeds, as well as by “blood refreshing” (i.e., introduction of single telic mating to another population or breed), which could generally be reflected in similar dynamics patterns of their demographic history and *N*_e_ changes in a series of generations.

Using the *N*_e*S*_ method [84], we also identified subtle changes in the estimated *N*_e_ curves (Figure 5c, Supplementary Figure S12), which are not visually detectable in the *N*_e_ plots (Figure 5a,b, Supplementary Figure S11) and provided more detailed information about changes in *N*_e_. This analysis revealed sharp fluctuations in *N*_e_ in CW that occurred approximately over the last 15–25 generations (Figure 5c). Less pronounced fluctuations in *N*_e_ were observed over the last 5–15 generations in some other populations, e.g., OMF, RC, YC, RW1, RW2, ZS and KJ. The earlier demographic history was characterized by approximately similar and uniform *N*_e*S*_ in all populations that corresponded to a relative increase in *N*_e_ change and was represented as a bunch of horizontal lines above zero on the *y*-axis (Figure 5c and Supplementary Figure S12).

In addition to the obtained plots of *N*_e_ change dynamics (Figure 5, Supplementary Figures S11 and S12), we characterized the respective breed distribution using PCA (Supplementary Figure S13A) and hierarchical clustering (Supplementary Figure S13B). When combining the *N*_e_ data for 3, 46 and 520–525 generations ago, the greatest distinctiveness and distance of the trendline relative to the other breeds was inherent in CW and, to a lesser extent, YC. Further, it was possible to observe the proximity for the following three pairs of breeds: WL–Ush, RW–Pm and OMF–ZS. The rest of the breeds formed a crowded core, which can generally indicate the similarity of their dynamics of *N*_e_ changes over the last 520–525 generations. Based on the combined data of *r*^2^, *dist* and *items* for three generations ago (not shown) as assessed using the SNP-based genome-wide analysis, the patterns of breed distribution by LD were also obtained (Supplementary Figure S14). The lowest LD values were characteristic of the ETBs of WL and RW, and the highest one was identified in the MEB of Kt, all three being located separately on the respective PCA plot (Supplementary Figure S14A). The remaining breeds formed two large clusters, one with slightly lower and the other with slightly higher LD values. Finally, a comprehensive assessment of the studied breeds by parameters showing the current *N*_e_ (three generations ago) and taking into account the three parameters of LD between SNP markers made it possible to identify four clusters using PCA (Supplementary Figure S15A) and hierarchical clustering (Supplementary Figure S15B) methods. One cluster involved the MTB of CW, the other one combined two ETBs, WL and RW, and all other breeds were distributed between two large clusters.

## 4. Discussion

To the best of our knowledge, this is the first thorough examination of the Russian chicken gene pool heritage using whole genome screening and focusing on its overall genetic diversity, phylogeny and demographic history. For this purpose, we employed 39,778 autosomal SNPs, explored genome-wide SNP genotypes amongst Russian native breeds developed in the Russian Empire and Soviet Union, along with the specialized foreign breeds.

Based on the performed analysis of the biodiversity of a large sample of native and foreign breeds, we can suggest the presence of very specific patterns of variability and genetic structure in the surveyed populations. For example, WL chickens were different from other breeds that are basically synthetic by origin. This appears to indicate a stronger and longer selection for egg production traits, which this line and the WL breed as a whole were subjected to, and, possibly, a narrower structural variability in the genomes of its ancestral forms. The greatest *_U_F*_IS_ value (0.103) was identified in the WL population, which, along with the lowest diversity indices, evidenced a severe selection pressure in this line of laying hens.

The advantage of examining and contrasting the 19 breeds that are typical examples of important, distinct and, in some ways, opposing evolutionary lineages occurred throughout the process of domesticating and breeding chickens [89] has been used in the current study. In particular, WL and RW were representatives of ETBs selected for fecundity, egg number and other egg production traits, CW for meat traits and the others for dual purpose. We discovered that 47.5 to 87.5% of the variability was attributed to allelic variation within the breeds, while 12.5 to 52.5% of the variability was brought on by genetic variations between the breeds compared in the pairwise mode (*F*_ST_ = 0.125 in the YC–RC pair to 0.525 in WL–NH). WL was characterized by less genetic variability than the other breeds (Table 1). The genetic drift that had a place in the WL line, which has undergone extensive selection for egg performance as a closed population, may be one explanation for this. A larger degree of genetic variation in CW chickens, however, may possibly be a result of crossbreeding of few diverse breeds used for developing CW. WL had the greatest *F*_ROH_ inbreeding coefficient (0.551), and YC and CW the lowest ones (0.154 and 0.162, respectively). This may be an indication that WL descended from a small number of founders (e.g., [91]), whereas YC and CW, in contrast, originated from a larger number of founders. WL may have an excess of homozygotes due to a higher level of selection pressure for egg production traits, while some other breeds, especially DPBs, with an excess of heterozygotes, are subject to selection for more diverse breeding targets in comparison with WL [92]. Another reason why the number of homozygotes or heterozygotes significantly deviated among the chicken breeds studied from what would be predicted under Hardy–Weinberg equilibrium is genetic drift.

The PCA plotting, phylogenetic analysis and admixture clustering results (Figure 1, Figure 2 and Figure 3, Supplementary Figure S9) suggested that the breeds were to a large extent consolidated and showed peculiar (i.e., breed-specific) admixture patterns. The genome-wide examination convincingly distinguished the breeds and supported their distinct genetic origins and selection history. In particular, two ETBs, WL and RW, demonstrated a close genetic kinship, with RW being previously exposed to crossing with WL and both breeds being bred for egg performance traits. Interestingly, RW1 and RW2 showed a clear differentiation, with RW2 being closer to WL, which suggested a varied genetic background and significant differences in genetic structure of these two RW populations. Previously, Abdelmanova et al. [42] suggested the differences between RW1 and RW2, only assuming what caused them. From the data obtained, it can be seen that RW2 was once again crossed with WL, in contrast to RW1. Inherently, RW1 is a purer population of this improved Russian breed. In contrast, CW developed from the crossing of GBs and MTBs and selected for characteristics related to growth, muscle development and meat production was located in an opposite cluster in Figure 1 and Figure 2. Overall, as shown in Figure 2, there were three major superclusters composed of the 20 breeds/populations studied. The first supercluster involved ETBs (WL, RW1 and RW2) and old Russian indigenous DPBs of OMF, Ush, RBB and YC. The second supercluster embraced one MTB (CW) and most of the DPBs, both of specialized foreign and Russian native origins. The third supercluster was made up by four Russian native DPBs (KJ, Kt, RC and ZS). Interestingly, there was a migration event from the common ancestor of WL and RW2 to Kt and ZS, confirming the known information about the origin of Kt and ZS. Collectively, our findings revealed genetic uniqueness of such Russian breeds as OMF, Ush, RBB, YC, KJ, Kt, RC and ZS, suggesting their further continued preservation and breeding.

Many breeds explored here and manifesting different phenotypic traits were previously included in other phylogenetic studies and showed similar relationship patterns (e.g., [15,16,17,18,19,20,37,89,93,94,95,96,97,98]). For instance, a thorough comparative phylogenetic assessment of several chicken breeds was conducted by Moiseyeva et al. [89] utilizing two sets of morphological discrete traits, body measurements, biochemical markers and the activity of serum esterase-1 (i.e., carboxylesterase 1 like 1, or CES1L1). In the created dendrograms reflecting evolutionary relationships in chickens, RW was grouped with WL and other ETBs, whereas CW formed shared clusters with GBs and MTBs [89]. Recently, Dementieva et al. [97] localized RW and WC on the opposing branches of an *F*_ST_-based Neighbor-Joining tree using the Illumina Chicken 60K SNP iSelect BeadChip. The RW and WC breeds, which represent typical ETBs and MTBs, were also used in our previous investigation [42] based on Chicken 50 K_CobbCons chip-assisted SNP genotypes. The earlier diversity and phylogenetic relationship analyses for RW, WC and other breeds were strongly validated by our present data, supported also by the admixture, gene flow and demographic history patterns we identified here.

Being at the junction between East and West, the vast territory of Russia has historically been a crossroads of trade routes, along which there was an intensive exchange of breeds of domestic animals, including poultry. Having settled in Russia, these breeds adapted to diverse and often harsh climatic conditions (e.g., [99]), and often lost their original breed traits, turning into local ecotypes and populations of mongrel animals or crossbreds, giving rise to new local breeds [100,101,102,103,104]. The degree of polymorphism in Russian indigenous breeds is comparable to that of Asian breeds generally. Russian breeds represent a mixed Eurasian group by origin and, therefore, included the gene pools of the two global regions, with a predominant contribution from the Asian region, which could predetermine their high polymorphism [3]. When considering the reasons for a greater variability of Russian breeds shown here in comparison with foreign breeds (mostly of Western origin), one should not forget about the large territory occupied by the Russian Empire and the USSR in the past and by the Russian Federation at present. Here, as for the whole Asian region, we should recognize the significance of the diversity of climatic, landscape and ethnic conditions for the emergence of many and different ecotypes and breeds of animals, including poultry [3].

## 5. Conclusions

Judging from the biodiversity analysis of this large sample of native and foreign breeds, we noted very specific patterns of variability and genetic structure of the surveyed breeds, with WL being especially different from other breeds of synthetic origin. This, apparently, may indicate a stronger and longer selection for egg-laying traits, which this line and the WL breed as a whole were subjected to, and, possibly, a narrower structural variation in the genomes of its ancestral stocks. The phylogenetic analysis results did not contradict the available data on the origin and breeding history of the breeds. For example, WL and RW are typical specialized ETBs selected for egg production; moreover, WL roosters were used for the creation of RW. Ush and RBB are old indigenous breeds that are tolerant to cold, have a brooding instinct and are also used for fancy purposes. RIR was an original breed for developing NH, and both were supposedly involved in the creation of PC. One of the initial breeds in the development of AS was Pm; in addition, when creating both breeds, YC was also used. ZS was used to produce Kt, and both breeds descended from NH and RW among other parent breeds. This historical breed information was in line with the present whole genome screening study.

In general, using the results of a multilateral survey of this sample of breeds, it can be argued that phylogenetic relationships based on their genetic structure and variability reflect the known, previously postulated and discovered patterns of evolution and selection in chickens [33,42,89,105,106,107]. These findings add to our understanding of genomic diversity, phylogeny and admixture in the genomes of unique Russian gene pool breeds with divergent selection histories and phenotypic features. The outcome of the current study will be beneficial for Russian chicken breed conservation, sustainable breeding and effective selection.

## Figures and Tables

**Table 1:** Chicken breeds (populations) screened in the present genome-wide study.

Breed (Population)		Code	*n*	Breed Type	Origin	Refs	Image Source
*Old Russian indigenous breeds*							
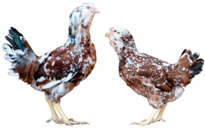	Orloff Mille Fleur	OMF	29	DPB/MEB, fancy and semi-game breed (GB), cold and heat tolerant, chicks are late feathering and cannot withstand cold and humidity, incubation instinct	Central Russia, late 18th century, from local chickens, Gilian and Old English Game	[32,33,37,43]	a
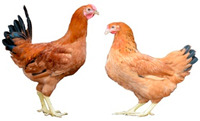	Poltava Clay	PC	21	DPB/MEB	Poltava Governorate, Russia; mid 19th century, from local chickens, Buff Orpington and possibly RIR and NH, Wyandotte, etc., bred at the Ukrainian Poultry Research Institute, USSR since 1951	[32,33,38,39,40,41]	a
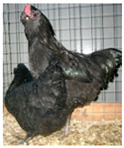	Russian Black Bearded (Galan)	RBB	25	DPB/MEB and fancy, cold tolerant, incubation instinct	Kursk, Oryol and neighboring governorates, Russia, 19th century (2nd half), from (1) Wyandotte and Crevecoeur or (2) Orloff Black and Wyandotte	[32]	b
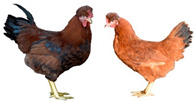	Russian Crested	RC	29	DPB/MEB and fancy, cold tolerant, incubation instinct	Russia, 19th century, from local chickens and possibly Asian breeds	[32]	a
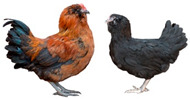	Ushanka (or Ukrainian Muffed)	Ush	30	DPB/MEB and fancy, sex-linked early feathering in chicks, cold tolerant, incubation instinct	South Russia and territory of Ukraine, 17th century to 1880s, from local chickens, probable random mating to other breeds	[32,33,37]	a
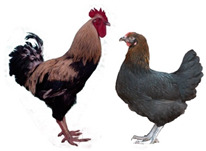	Yurlov Crower	YC	36	DPB/MEB, long crowing, of two varieties (silver and golden), sex-linked late feathering in chicks	Russia, 19th century (2nd half), from local and GB chickens, Brahma, Cochin and Langshan, almost extinct in 1941–1945, brought to ARPRTI, Zagorsk, USSR in 1948	[32,33,34,35,36]	a
*Improved Russian breeds*							
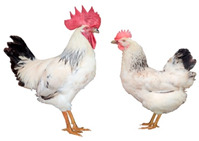	Adler Silver	AS	30	DPB/MEB	Adler Poultry Farm, Krasnodar Krai, USSR, 1951–1965, from Pm, RW, NH, White Plymouth Rock and YC	[32]	a
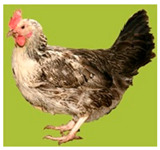	Kotlyarevsky	Kt	28	DPB/MEB	Kotlyarevsky Breeding Farm, Kabardino-Balkarian ASSR, USSR, 20th century (2nd half), from NH, RW, ZS, Naked Neck and Barred Plymouth Rock	[32]	b
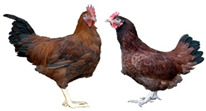	Kuchino Jubilee	KJ	29	DPB/MEB, sex-linked early feathering in chicks	Kuchinsky State Breeding Farm, Moscow Oblast, USSR, 1947–1990, from RW, NH, RIR, AoB, Plymouth Rock White, YC, Brown Leghorn (Italian Partridge) and Livny	[32]	a
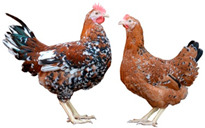	Leningrad Mille Fleur	LMF	7	DPB/EMB	RRIFAGB, Pushkin, Leningrad Oblast, USSR, 1985, from Australorp Black Speckled, NH and PC	[33]	a
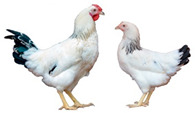	Pervomai	Pm	30	DPB/MEB	Pervoe Maya State Farm, Kharkov Oblast, Ukrainian SSR, USSR, 1935–1941; Pachelma State Farm, Penza Oblast, RSFSR, USSR, 1942–1963; from White Wyandotte, RIR and YC	[32,33]	a
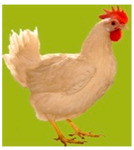	Russian White (ARPRTI)	RW1	29	ETB	USSR, 1929–1953, from local white chickens and WL, bred at ARPRTI, Zagorsk/Sergiev Posad, USSR/Russia	[32,37,42]	b
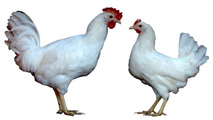	Russian White (RRIFAGB)	RW2	71	ETB, cold tolerant	Bred at RRIFAGB, Pushkin, USSR/Russia since 1952, a single telic mating of an RRIFAGB inbred line of RW, selected for cold tolerance, to WL	[32,33,37,42,44,45,46]	a
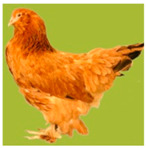	Ushanka Foot-feathered	UshF	6	DPB/MEB and fancy, cold tolerant	ARPRTI, Sergiev Posad, Russia, 20th century (more recently), from Pavlov, Orloff and possibly Ush	[32]	b
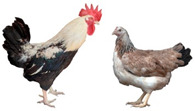	Zagorsk Salmon	ZS	27	DPB/MEB, sex-linked late feathering and down color in chicks	Zagorsk, USSR, 1950–1959, from RW, NH, RIR and YC	[32,33]	a
*Specialized foreign breeds*							
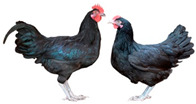	Australorp Black	AoB	30	DPB/MEB	Australia, 1890s to 1929, from Black Orpington, RIR, Black Minorca, WL, Langshan and Barred Plymouth Rock, bred in Russia since 1946	[32,33]	a
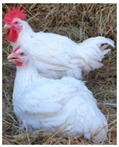	Cornish White	CW	24	MTB	England, 1886, from local GB chickens, Asil, White Malay, Indian Game and Cochin, used as paternal stock in commercial broiler crosses, bred in Russia	[33,37,42]	a
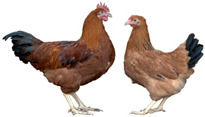	New Hampshire	NH	10	DPB/MEB	USA, early 20th century to 1935, from RIR	[32,33]	a
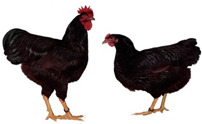	Rhode Island Red	RIR	17	DPB/EMB	States of Rhode Island and Massachusetts, USA, 1880s to 1904, from Cochin, Java, Malay, Shanghai and Brown Leghorn	[32,47]	a
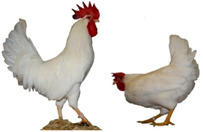	White Leghorn	WL	20	ETB, the only Mediterranean breed of economic importance today	Tuscany, Italy, 19th century, from light rural breeds, brought to USA in 1828, a white variety imported to USA in 1853, line G11 bred in Germany since 1965	[48,49]	a

Image sources: a, authors’ own photographs; b, owned by one of the authors, D.V.A., and reproduced by him on his website (Ref. [32]).

**Table 2:** Descriptive statistics for genetic diversity ^1^ in the studied breeds (populations) based on SNP genotypes.

Breed	*H_O_* (M ± SE)	*H_E_* (M ± SE)	*_U_H_E_* (M ± SE)	*A_R_* (M ± SE)	*F*_IS_ [CI 95%]	*_U_F*_IS_ [CI 95%]
*Old Russian indigenous breeds*						
OMF	0.291 ± 0.001	0.286 ± 0.001	0.291 ± 0.001	1.709 ± 0.002	−0.019 [−0.021; −0.017]	−0.001 [−0.003; 0.001]
PC	0.257 ± 0.001	0.249 ± 0.001	0.255 ± 0.001	1.655 ± 0.002	−0.029 [−0.031; −0.027]	−0.003 [ −0.005; −0.001]
RBB	0.250 ± 0.001	0.247 ± 0.001	0.252 ± 0.001	1.624 ± 0.002	−0.008 [−0.010; −0.006]	0.012 [0.010; 0.014]
RC	0.325 ± 0.001	0.327 ± 0.001	0.333 ± 0.001	1.819 ± 0.002	0.005 [0.003; 0.007]	0.022 [0.020; 0.024]
Ush	0.249 ± 0.001	0.255 ± 0.001	0.259 ± 0.001	1.647 ± 0.002	0.016 [0.014; 0.018]	0.033 [0.031; 0.035]
YC	0.347 ± 0.001	0.358 ± 0.001	0.363 ± 0.001	1.867 ± 0.001	0.029 [0.027; 0.031]	0.043 [0.041; 0.045]
Av.	0.287 ± 0.017	0.287 ± 0.019	0.292 ± 0.019	1.720 ± 0.041	−0.001 ± 0.009 [−0.031; 0.031]	0.018 ± 0.008 [−0.005; 0.045]
*Improved Russian breeds*						
AS	0.321 ± 0.001	0.329 ± 0.001	0.335 ± 0.001	1.811 ± 0.002	0.020 [0.018; 0.022]	0.037 [0.035; 0.039]
KJ	0.256 ± 0.001	0.252 ± 0.001	0.256 ± 0.001	1.665 ± 0.002	−0.018 [−0.020; −0.016]	0.000 [−0.002; 0.002]
Kt	0.257 ± 0.001	0.256 ± 0.001	0.261 ± 0.001	1.650 ± 0.002	−0.006 [−0.008; −0.004]	0.012 [0.010; 0.014]
LMF	0.249 ± 0.001	0.228 ± 0.001	0.246 ± 0.001	1.606 ± 0.002	−0.090 [−0.094; −0.086]	−0.011 [−0.015; −0.007]
Pm	0.291 ± 0.001	0.290 ± 0.001	0.295 ± 0.001	1.729 ± 0.002	−0.006 [−0.008; −0.004]	0.011 [0.009; 0.013]
RW1	0.255 ± 0.001	0.248 ± 0.001	0.252 ± 0.001	1.632 ± 0.002	−0.016 [−0.018; −0.014]	0.001 [−0.001; 0.003]
RW2	0.306 ± 0.001	0.305 ± 0.001	0.307 ± 0.001	1.763 ± 0.002	−0.001 [−0.002; 0.000]	0.006 [0.005; 0.007]
UshF	0.252 ± 0.001	0.232 ± 0.001	0.253 ± 0.001	1.645 ± 0.003	−0.088 [−0.092; −0.084]	0.003 [−0.001; 0.007]
ZS	0.275 ± 0.001	0.271 ± 0.001	0.276 ± 0.001	1.688 ± 0.002	−0.015 [−0.017; −0.013]	0.004 [0.002; 0.006]
Av.	0.274 ± 0.009	0.268 ± 0.011	0.276 ± 0.010	1.688 ± 0.022	−0.024 ± 0.013 [−0.094; 0.022]	0.007 ± 0.004 [−0.015; 0.039]
*Specialized foreign breeds*						
AoB	0.296 ± 0.001	0.314 ± 0.001	0.319 ± 0.001	1.777 ± 0.002	0.045 [0.043; 0.047]	0.061 [0.059; 0.063]
CW	0.365 ± 0.001	0.368 ± 0.001	0.371 ± 0.001	1.876 ± 0.001	0.001 [−0.001; 0.003]	0.022 [0.020; 0.024]
NH	0.233 ± 0.001	0.205 ± 0.001	0.216 ± 0.001	1.542 ± 0.002	−0.122 [−0.125; −0.119]	−0.067 [−0.070; −0.064]
RIR	0.256 ± 0.001	0.256 ± 0.001	0.264 ± 0.001	1.661 ± 0.002	−0.003 [−0.006; 0.000]	0.027 [0.024; 0.030]
WL	0.164 ± 0.001	0.185 ± 0.001	0.185 ± 0.001	1.452 ± 0.002	0.080 [0.077; 0.083]	0.103 [0.100; 0.106]
Av.	0.263 ± 0.033	0.266 ± 0.033	0.271 ± 0.034	1.662 ± 0.077	0.000 ± 0.034 [−0.125; 0.083]	0.029 ± 0.028 [−0.070; 0.106]

^1^ *H_O_*, observed heterozygosity; M, mean value; SE, standard error; *H_E_*, expected heterozygosity; *_U_H_E_*, unbiased expected heterozygosity%]; *A_R_*, rarefied allelic richness; *F*_IS_, inbreeding coefficient [CI 95%, range variation of *_U_F*_IS_ coefficient at a confidence interval of 95%]; *_U_F*_IS_, unbiased inbreeding coefficient; Av., average (M ± SE). All pairwise breed differences were significant at *p* < 0.001. Breed codes are given in Table 1.

**Table 3:** Estimation of genetic variation ^1^ for the three major breed groups using AMOVA.

Source of Variation	df ^1^	Sum of Squares	Variance Components	Percentage of Variation	*p*-Value of Fixation
Among groups	2	450,114,870.5	0.014	1.36	0.011
Among populations within group	17	4,945,631,101	0.151	14.93	0.001
Within populations	508	27,729,967,336	0.163	83.71	0.001
Total	527	33,125,713,307	0.328	100	

^1^ Degree of freedom.

**Table 4:** Runs of homozygosity (ROHs) descriptive statistics ^1^ for the studied breeds.

Breed	ROH Length, Mb (M ± SE)	ROH No. (M ± SE)	*F*_ROH_ (M ± SE)
*Old Russian indigenous breeds*			
OMF	266.42 ± 12.42	125.17 ± 4.03	0.279 ± 0.013
PC	337.93 ± 15.74	145.00 ± 6.50	0.354 ± 0.017
RBB	369.41 ± 16.07	141.32 ± 5.01	0.387 ± 0.017
RC	197.84 ± 19.08	89.28 ± 6.88	0.207 ± 0.020
Ush	375.63 ± 14.23	127.13 ± 2.70	0.394 ± 0.015
YC	146.91 ± 11.94	70.11 ± 2.62	0.154 ± 0.013
Average (M ± SE)	282.36 ± 38.78	121.75 ± 12.26	0.296 ± 0.041
*Improved Russian breeds*			
AS	205.05 ± 9.33	94.87 ± 2.86	0.215 ± 0.010
KJ	368.96 ± 7.20	142.00 ± 2.71	0.387 ± 0.008
Kt	360.79 ± 9.22	143.86 ± 3.83	0.378 ± 0.010
LMF	375.11 ± 18.37	112.14 ± 4.13	0.393 ± 0.019
Pm	274.02 ± 9.01	139.87 ± 3.77	0.287 ± 0.009
RW1	323.45 ± 15.34	151.38 ± 2.41	0.339 ± 0.016
RW2	195.24 ± 5.89	130.83 ± 2.74	0.205 ± 0.006
UshF	357.71 ± 35.25	116.00 ± 4.56	0.375 ± 0.037
ZS	301.06 ± 9.48	154.04 ± 4.9	0.316 ± 0.010
Average (M ± SE)	306.82 ± 23.04	131.67 ± 6.66	0.322 ± 0.024
*Specialized foreign breeds*			
AoB	260.03 ± 10.18	107.03 ± 2.89	0.273 ± 0.011
CW	154.61 ± 5.98	126.29 ± 3.28	0.162 ± 0.006
NH	403.39 ± 11.15	120.80 ± 1.43	0.423 ± 0.012
RIR	352.15 ± 14.42	151.94 ± 8.01	0.369 ± 0.015
WL	525.64 ± 6.12	249.75 ± 2.48	0.551 ± 0.006
Average (M ± SE)	339.16 ± 63.00	151.16 ± 25.70	0.356 ± 0.066

^1^ ROH No., number of ROHs in a genome; Mb, megabases; M, mean value; SE, standard error; ROH Length, overall length of ROHs in a genome; *F*_ROH_, inbreeding coefficient calculated based on ROHs.

## Data Availability

The genotyping data presented in this study can be shared with third parties upon approval from the GWMAS Consortium.

## Supplementary Materials

The following are available online at https://www.mdpi.com/article/10.3390/biology12070979/s1. Table S1: Statistics for diversity and runs of homozygosity when grouping breeds by origin. Table S2: Pairwise *F*_ST_-based interbreed genetic distances. Table S3: Pairwise Reynolds et al. [62] distances between the chicken breeds studied. Table S4: Effective population size changes over recent 50–500 generations. Figure S1: Distribution of SNP-genotyped chicken breeds into four clusters depending on their genetic variability. Figure S2: Distribution of SNP-genotyped chicken breeds into five clusters depending on their genetic structure. Figure S3: Manhattan plots of the distribution of runs of homozygosity (ROH) islands in each chicken breed. Figure S4: 3D PCA plot for the studied chicken breeds/populations based on genome-wide SNP genotypes. Figure S5: Unrooted Neighbor-Joining phylogenetic trees reconstructed for the 20 studied chicken breeds/populations based on the *F*_ST_ genetic distances. Figure S6: Unrooted radial Neighbor-Joining phylogenetic trees for 17 breeds (except KJ and NH) according to the results of SNP genotyping. Figure S7: Plot of cross-validation errors (CV error). Figure S8: Admixture-derived [66,67] structure of studied populations in the form of a semicircle. Figure S9: Admixture-derived structure of the studied populations with K = 2 to 26 ancestral populations. Figure S10: Assessment of the degree of divergence and the level of gene flow between the studied breeds. Figure S11: Dynamics of changes in the effective population size (*N*_e_). Figure S12: Analysis of the *N*_e_ slopes [84]. Figure S13: Distribution of the 17 studied breeds (except KJ and NH) when combining the *N*_e_ data for 3, 46 and 520–525 generations ago. Figure S14: Distribution of the 17 studied breeds (except KJ and NH) when combining the *r*^2^, *dist* and *items* data for 3 generations ago. Figure S15: Distribution of the 17 studied breeds (except KJ and NH) when combining the *N*_e_, *r*^2^, *dist* and *items* data for 3 generations ago.
