# Supplementary material for: Whole Genome Screening Procures a Holistic Hold of the Russian Chicken Gene Pool Heritage and Demographic History
Source: Biology (Basel). 2023 Jul 10;12(7):979. doi: 10.3390/biology12070979 (PMC10376169; doi:10.3390/biology12070979)
Supplement: Supplementary file 1 [file biology-12-00979-s001.zip › Suppl Figure S9 edited.pdf]

Supplementary Figure S9. ADMIXTURE-derived structure of the studied populations with K = 2 to 26 ancestral populations.

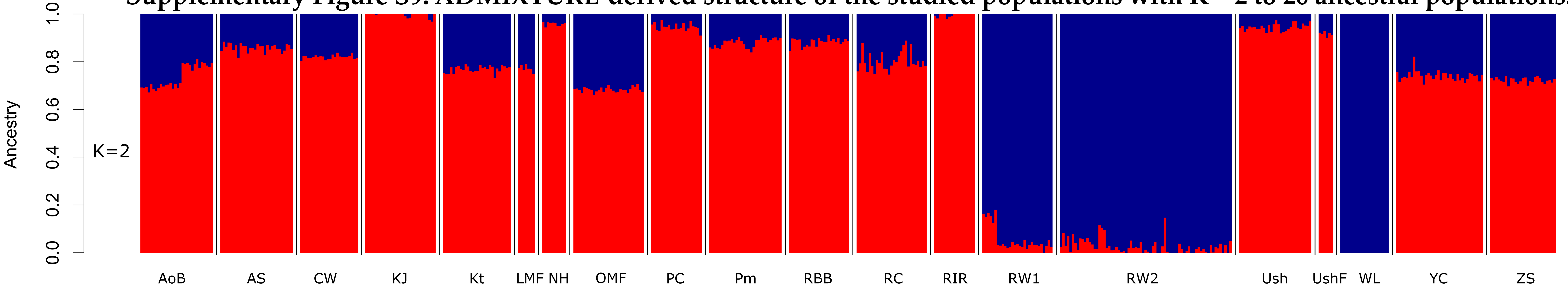

Each admixture diagram represents the cluster structure of the studied breed at the given number K of clusters.

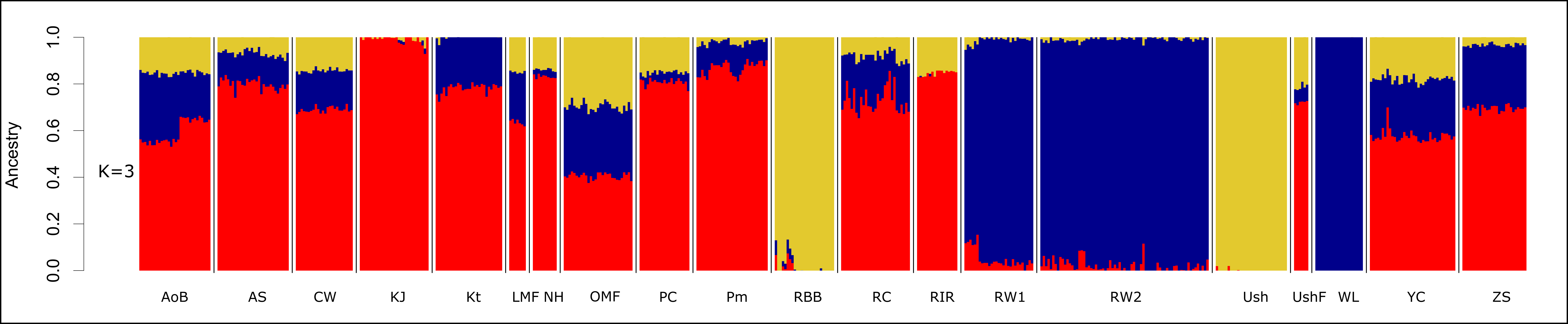

Ancestry
K=3
0.0
0.2
0.4
0.6
0.8
1.0
AoB
AS
CW
KJ
Kt
LMF
NH
OMF
PC
Pm
RBB
RC
RIR
RW1
RW2
Ush
UshF
WL
YC
ZS

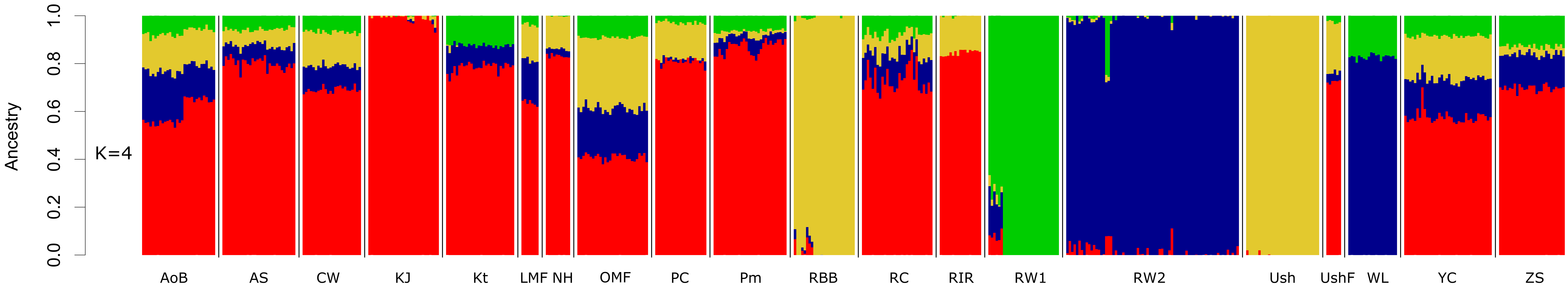

Ancestry
1.0
0.8
0.6
0.4
0.2
0.0
K=4
AoB
AS
CW
KJ
Kt
LMF
NH
OMF
PC
Pm
RBB
RC
RIR
RW1
RW2
Ush
UshF
WL
YC
ZS

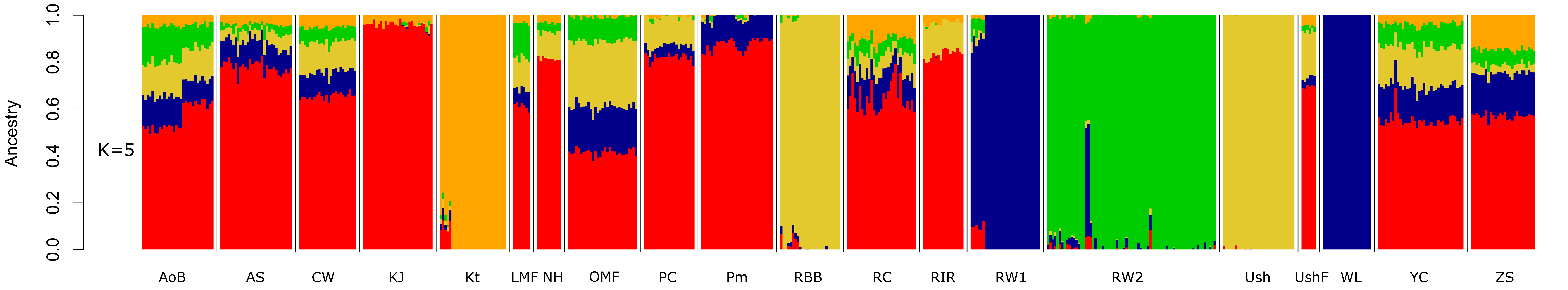
Ancestry
1.0
0.8
0.6
0.4
0.2
0.0
K=5
AoB
AS
CW
KJ
Kt
LMF
NH
OMF
PC
Pm
RBB
RC
RIR
RW1
RW2
Ush
UshF
WL
YC
ZS

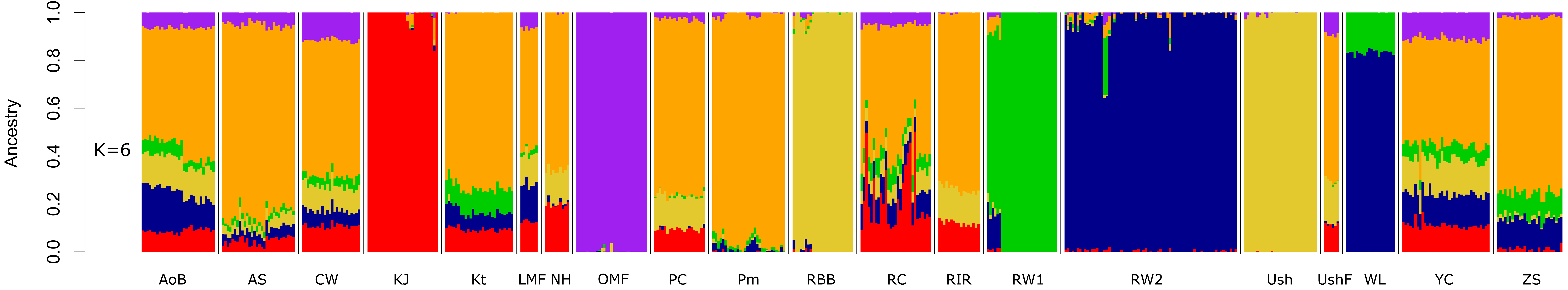

Ancestry
1.0
0.8
0.6
0.4
0.2
0.0
K=6
AoB
AS
CW
KJ
Kt
LMF
NH
OMF
PC
Pm
RBB
RC
RIR
RW1
RW2
Ush
UshF
WL
YC
ZS

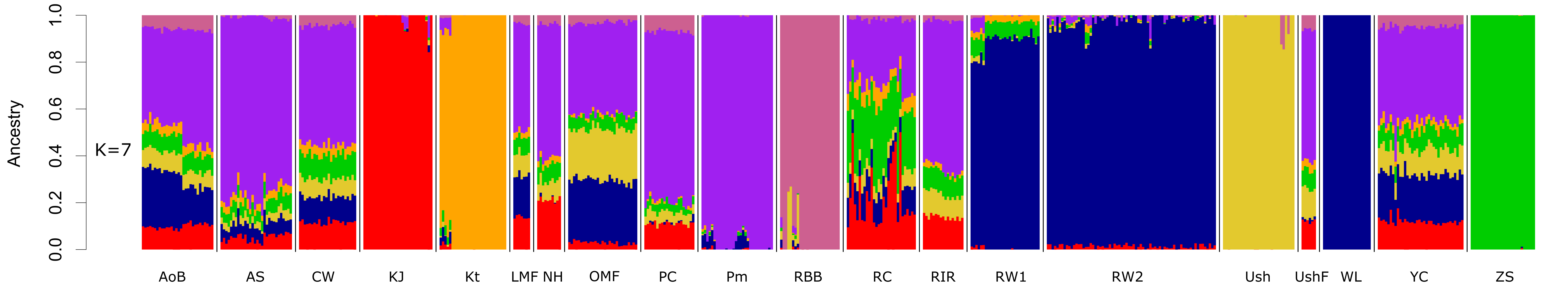
Ancestry
1.0
0.8
0.6
0.4
0.2
0.0
K=7
AoB
AS
CW
KJ
Kt
LMF
NH
OMF
PC
Pm
RBB
RC
RIR
RW1
RW2
Ush
UshF
WL
YC
ZS

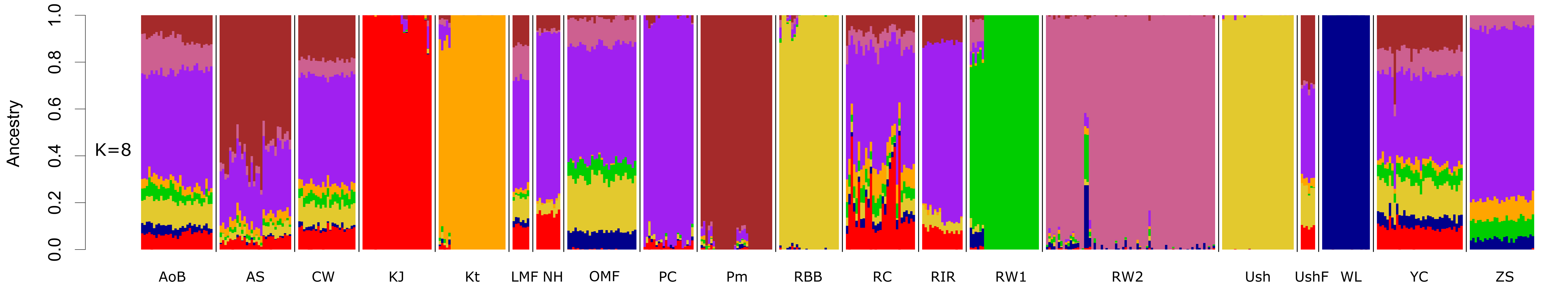

Ancestry
1.0
0.8
0.6
0.4
0.2
0.0
K=8
AoB
AS
CW
KJ
Kt
LMF
NH
OMF
PC
Pm
RBB
RC
RIR
RW1
RW2
Ush
UshF
WL
YC
ZS

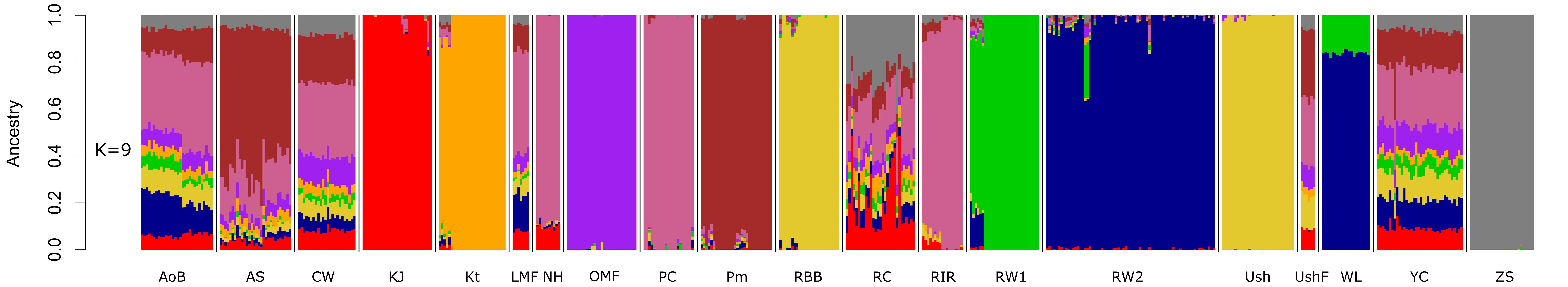
Ancestry
1.0
0.8
0.6
0.4
0.2
0.0
K=9
AoB
AS
CW
KJ
Kt
LMF
NH
OMF
PC
Pm
RBB
RC
RIR
RW1
RW2
Ush
UshF
WL
YC
ZS

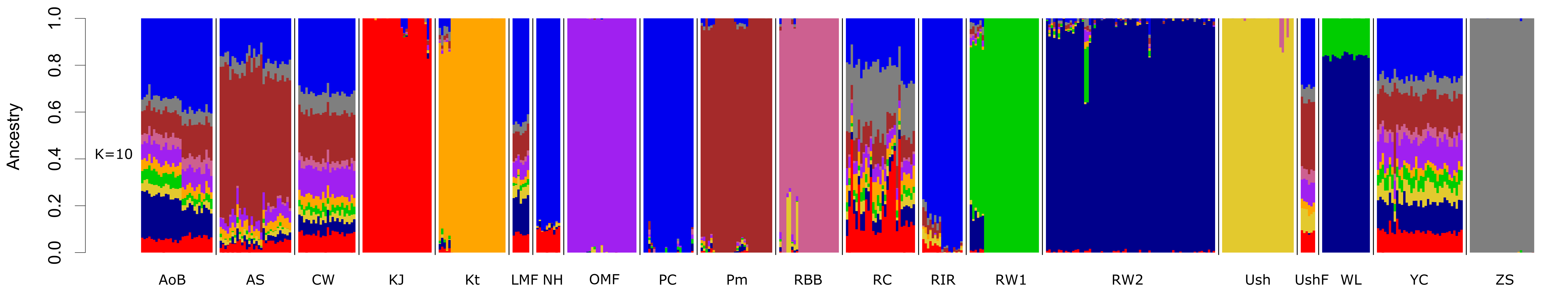

Ancestry
1.0
0.8
0.6
0.4
0.2
0.0
K=10
AoB
AS
CW
KJ
Kt
LMF
NH
OMF
PC
Pm
RBB
RC
RIR
RW1
RW2
Ush
UshF
WL
YC
ZS

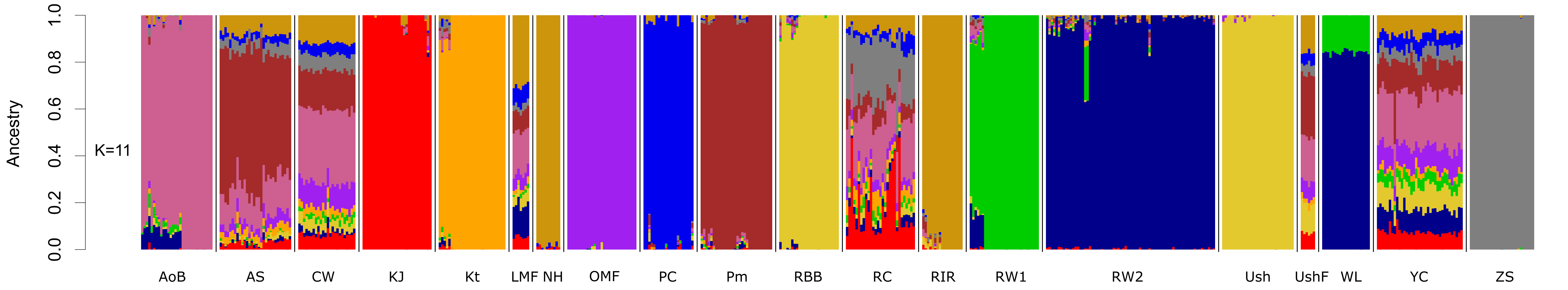
Ancestry
1.0
0.8
0.6
0.4
0.2
0.0
K=11
AoB
AS
CW
KJ
Kt
LMF
NH
OMF
PC
Pm
RBB
RC
RIR
RW1
RW2
Ush
UshF
WL
YC
ZS

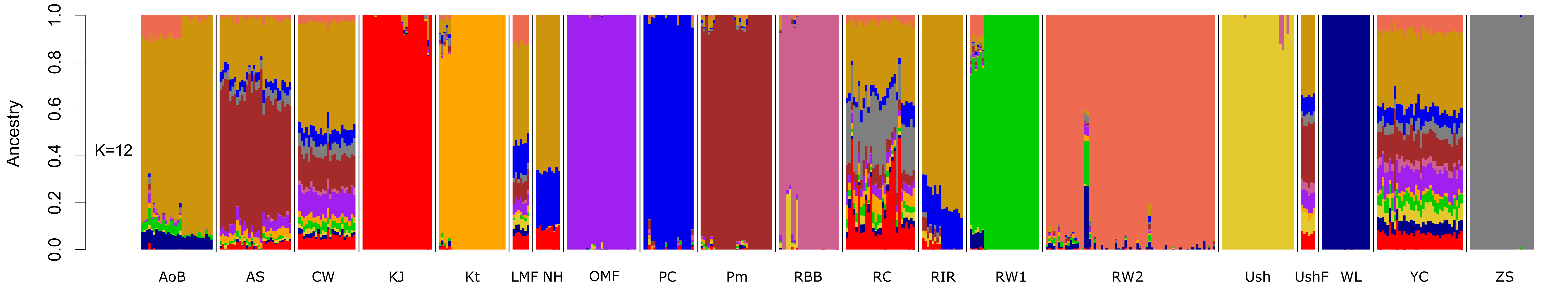

Ancestry
0.0
0.2
0.4
0.6
0.8
1.0
K=12
AoB
AS
CW
KJ
Kt
LMF
NH
OMF
PC
Pm
RBB
RC
RIR
RW1
RW2
Ush
UshF
WL
YC
ZS

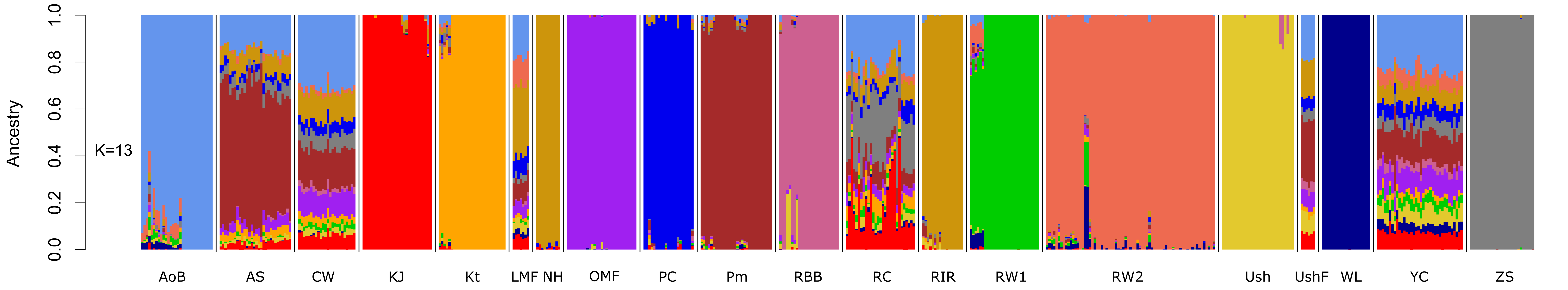

Ancestry
1.0
0.8
0.6
0.4
0.2
0.0
K=13
AoB
AS
CW
KJ
Kt
LMF
NH
OMF
PC
Pm
RBB
RC
RIR
RW1
RW2
Ush
UshF
WL
YC
ZS

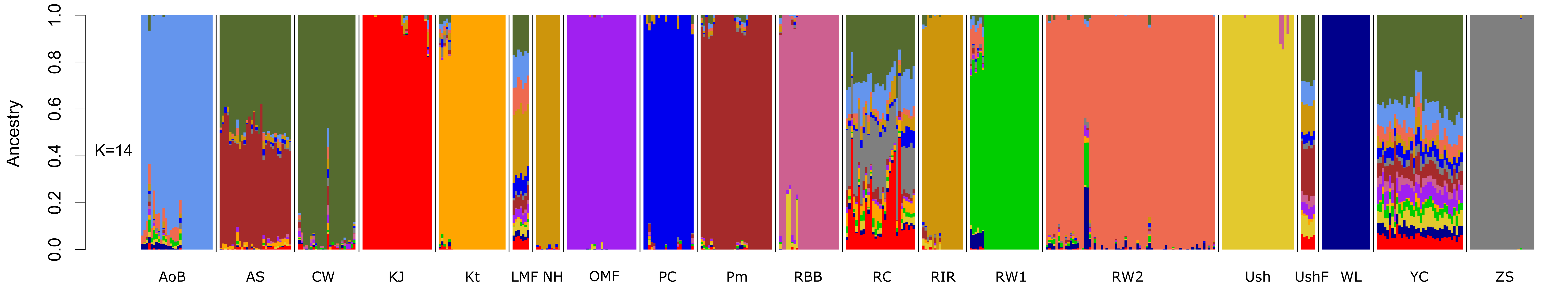
Ancestry
0.0
0.2
0.4
0.6
0.8
1.0
K=14
AoB
AS
CW
KJ
Kt
LMF
NH
OMF
PC
Pm
RBB
RC
RIR
RW1
RW2
Ush
UshF
WL
YC
ZS

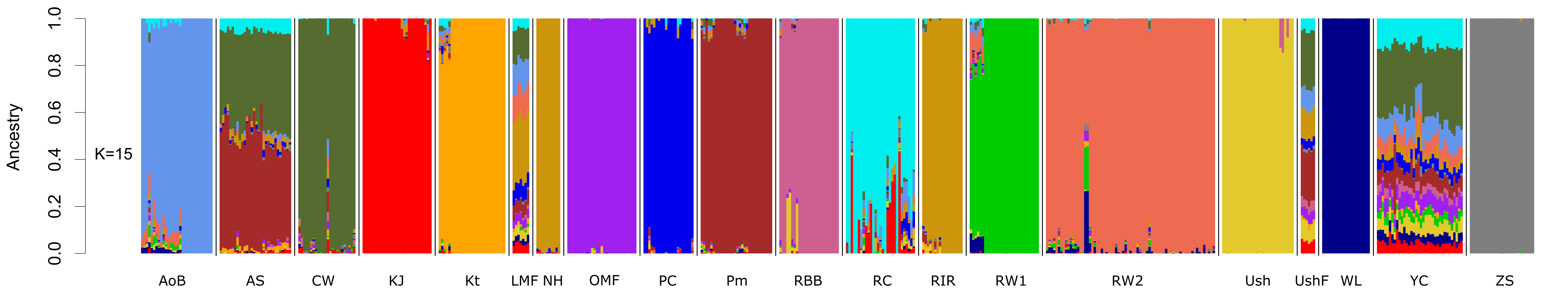

Ancestry
1.0
0.8
0.6
0.4
0.2
0.0
K=15
AoB
AS
CW
KJ
Kt
LMF
NH
OMF
PC
Pm
RBB
RC
RIR
RW1
RW2
Ush
UshF
WL
YC
ZS

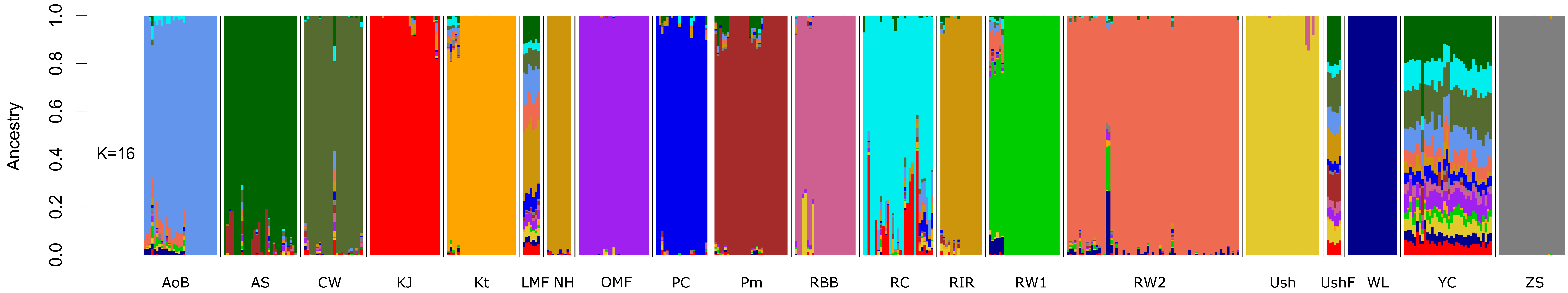
Ancestry
1.0
0.8
0.6
0.4
0.2
0.0
K=16
AoB
AS
CW
KJ
Kt
LMF
NH
OMF
PC
Pm
RBB
RC
RIR
RW1
RW2
Ush
UshF
WL
YC
ZS

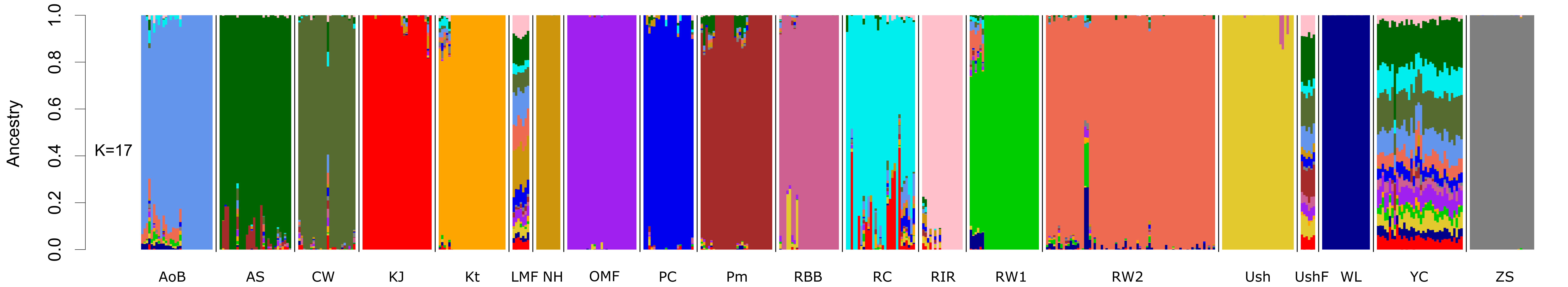
Ancestry
0.0
0.2
0.4
0.6
0.8
1.0
K=17
AoB
AS
CW
KJ
Kt
LMF
NH
OMF
PC
Pm
RBB
RC
RIR
RW1
RW2
Ush
UshF
WL
YC
ZS

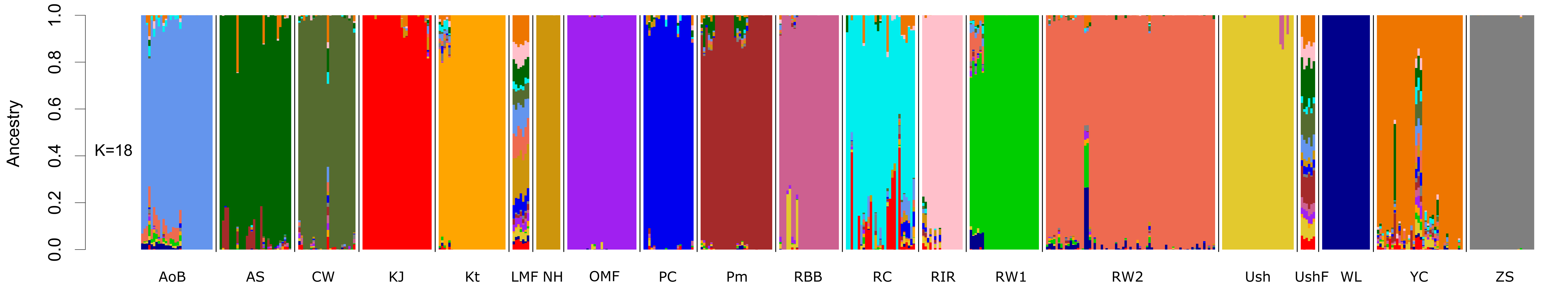

Ancestry
1.0
0.8
0.6
0.4
0.2
0.0
K=18
AoB
AS
CW
KJ
Kt
LMF
NH
OMF
PC
Pm
RBB
RC
RIR
RW1
RW2
Ush
UshF
WL
YC
ZS

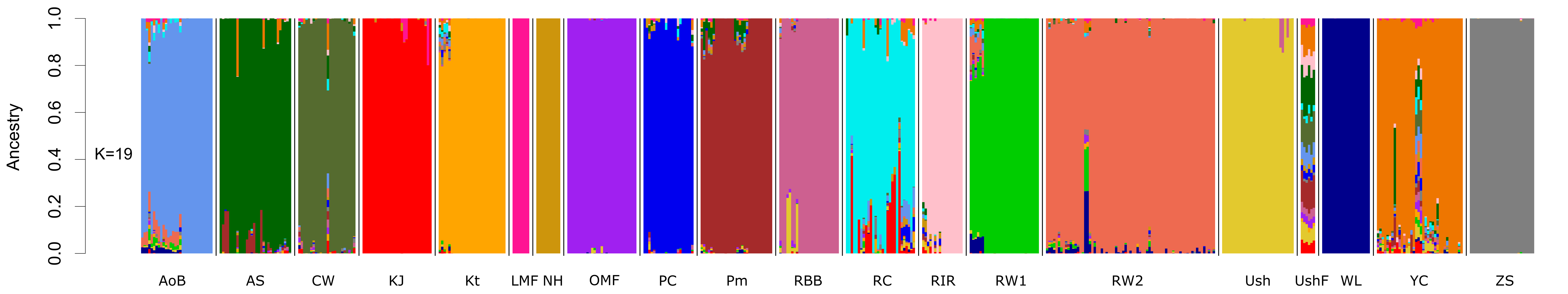

Ancestry
1.0
0.8
0.6
0.4
0.2
0.0
K=19
AoB
AS
CW
KJ
Kt
LMF
NH
OMF
PC
Pm
RBB
RC
RIR
RW1
RW2
Ush
UshF
WL
YC
ZS

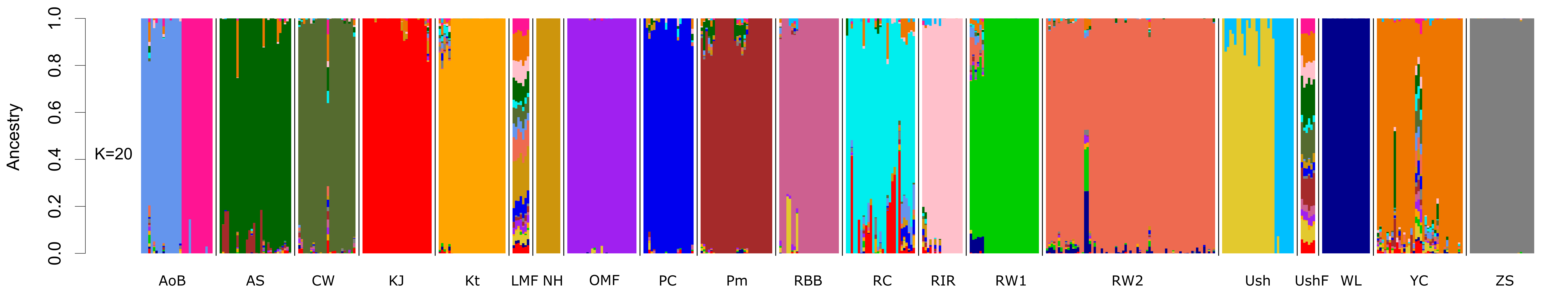
Ancestry
1.0
0.8
0.6
0.4
0.2
0.0
K=20
AoB
AS
CW
KJ
Kt
LMF
NH
OMF
PC
Pm
RBB
RC
RIR
RW1
RW2
Ush
UshF
WL
YC
ZS

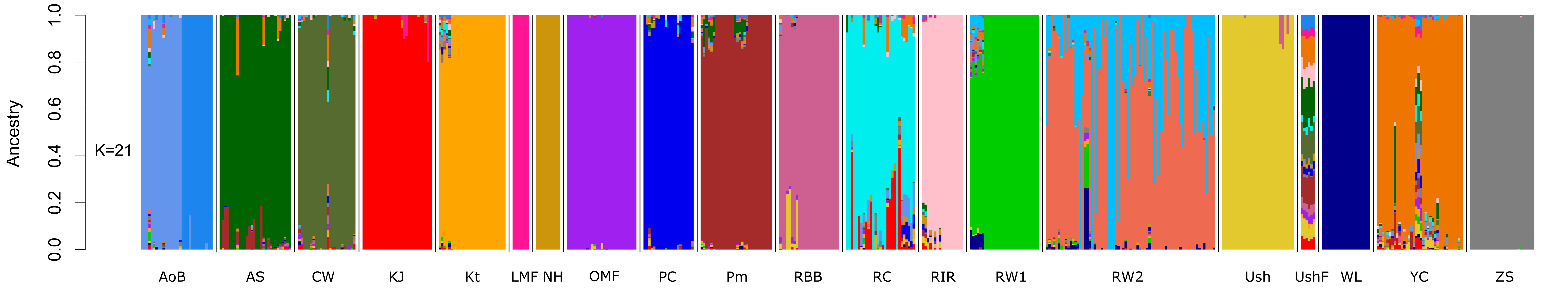

Ancestry
0.0
0.2
0.4
0.6
0.8
1.0
K=21
AoB
AS
CW
KJ
Kt
LMF
NH
OMF
PC
Pm
RBB
RC
RIR
RW1
RW2
Ush
UshF
WL
YC
ZS

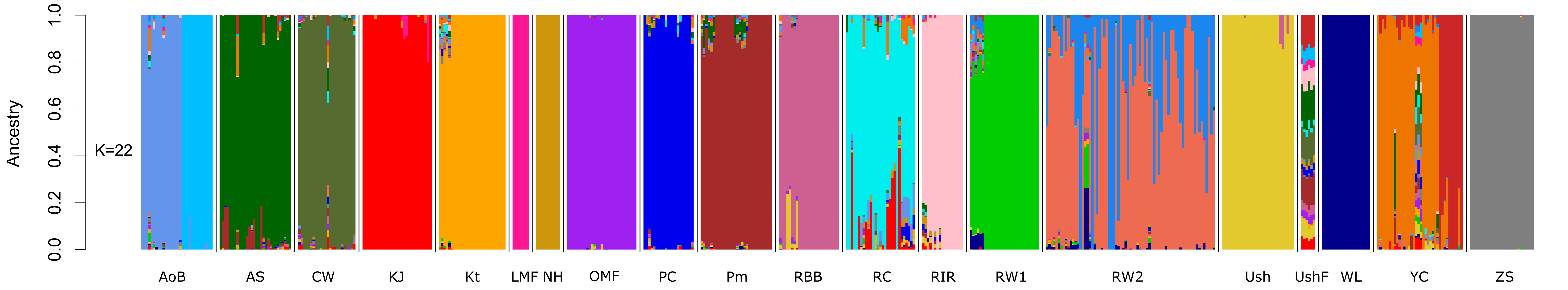
Ancestry
1.0
0.8
0.6
0.4
0.2
0.0
K=22
AoB
AS
CW
KJ
Kt
LMF
NH
OMF
PC
Pm
RBB
RC
RIR
RW1
RW2
Ush
UshF
WL
YC
ZS

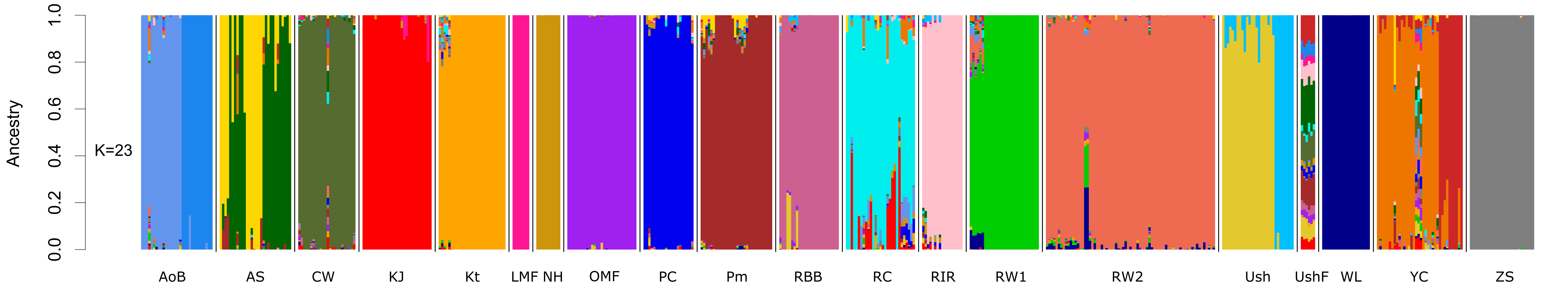

Ancestry
1.0
0.8
0.6
0.4
0.2
0.0
K=23
AoB
AS
CW
KJ
Kt
LMF
NH
OMF
PC
Pm
RBB
RC
RIR
RW1
RW2
Ush
UshF
WL
YC
ZS

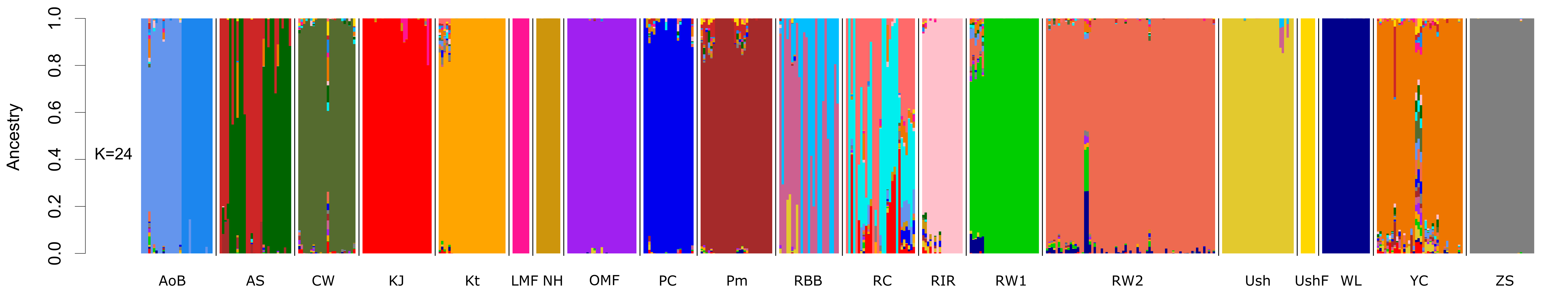
Ancestry
1.0
0.8
0.6
0.4
0.2
0.0
K=24
AoB
AS
CW
KJ
Kt
LMF
NH
OMF
PC
Pm
RBB
RC
RIR
RW1
RW2
Ush
UshF
WL
YC
ZS

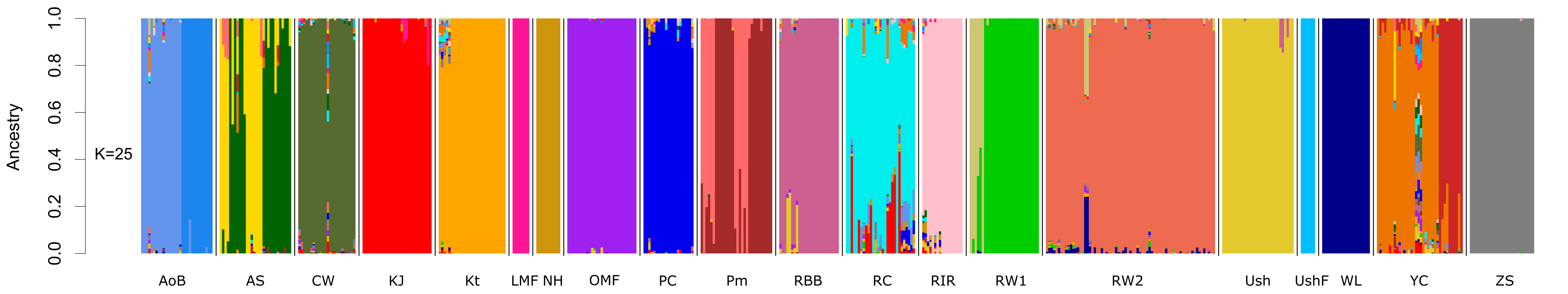

Ancestry
0.0
0.2
0.4
0.6
0.8
1.0
K=25
AoB
AS
CW
KJ
Kt
LMF
NH
OMF
PC
Pm
RBB
RC
RIR
RW1
RW2
Ush
UshF
WL
YC
ZS

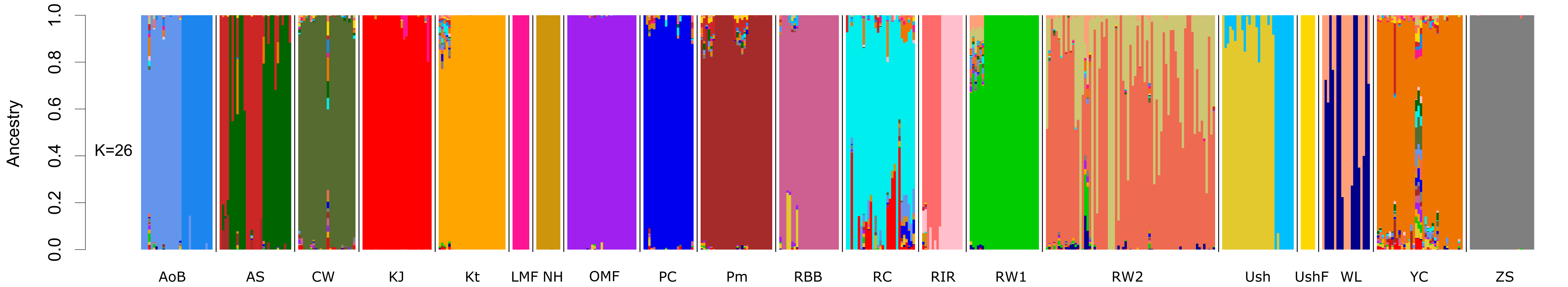
Ancestry
1.0
0.8
0.6
0.4
0.2
0.0
K=26
AoB
AS
CW
KJ
Kt
LMF
NH
OMF
PC
Pm
RBB
RC
RIR
RW1
RW2
Ush
UshF
WL
YC
ZS
