# Supplementary material for: Whole Genome Screening Procures a Holistic Hold of the Russian Chicken Gene Pool Heritage and Demographic History
Source: Biology (Basel). 2023 Jul 10;12(7):979. doi: 10.3390/biology12070979 (PMC10376169; doi:10.3390/biology12070979)
Supplement: Supplementary file 1 [file biology-12-00979-s001.zip › Suppl Figures S10¿CS15 edited.pdf]

A

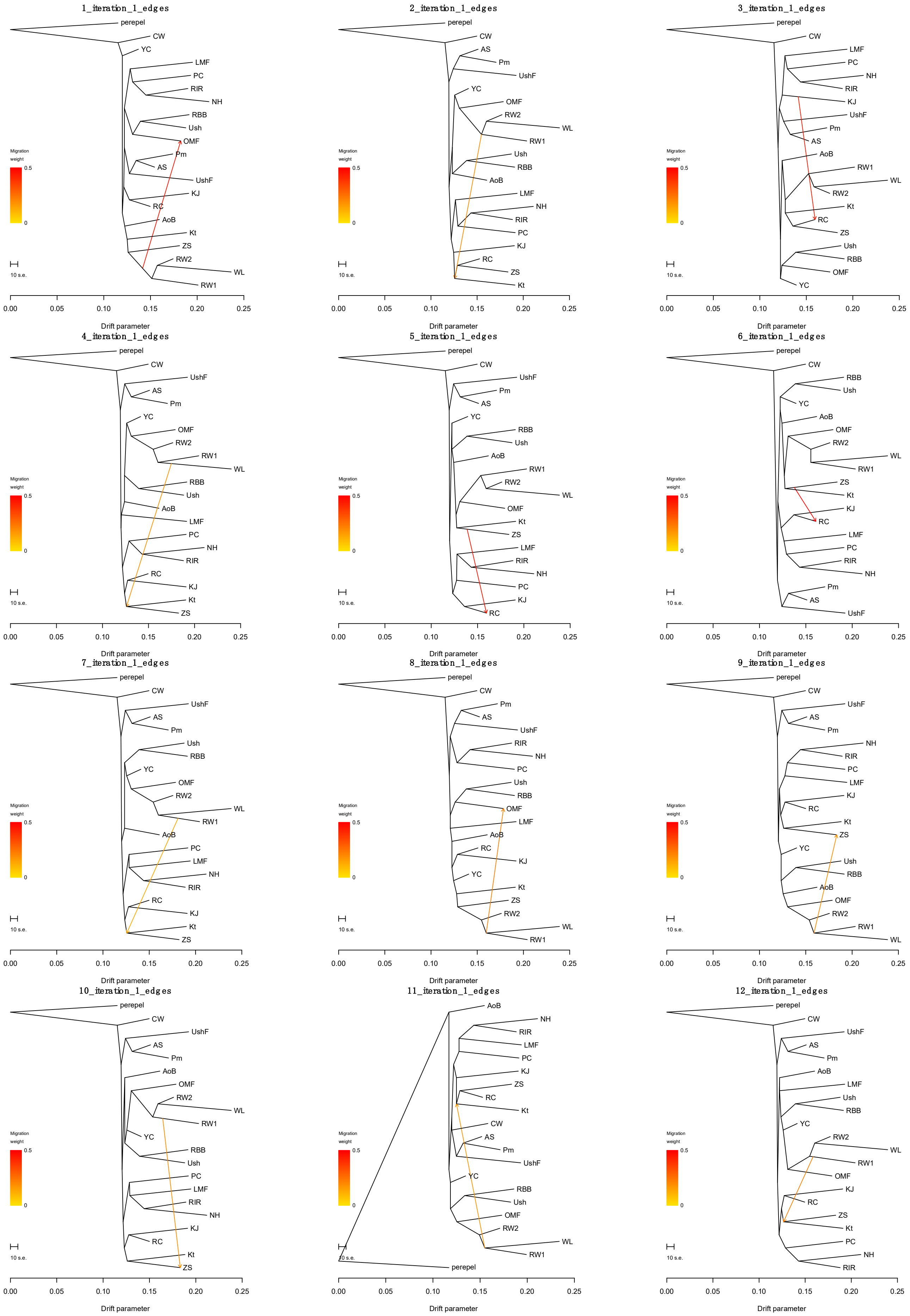

1_iteration_1_edges
perepel
CW
YC
LMF
PC
RIR
NH
RBB
Ush
OMF
Pm
AS
UshF
KJ
RC
AoB
Kt
ZS
RW2
WL
RW1
2_iteration_1_edges
perepel
CW
AS
Pm
UshF
YC
OMF
RW2
WL
RW1
Ush
RBB
AoB
LMF
NH
RIR
PC
KJ
RC
ZS
Kt
3_iteration_1_edges
perepel
CW
LMF
PC
NH
RIR
KJ
UshF
Pm
AS
AoB
RW1
WL
RW2
Kt
RC
ZS
Ush
RBB
OMF
YC
4_iteration_1_edges
perepel
CW
UshF
AS
Pm
YC
OMF
RW2
RW1
WL
RBB
Ush
AoB
LMF
PC
NH
RIR
RC
KJ
Kt
ZS
5_iteration_1_edges
perepel
CW
UshF
Pm
AS
YC
RBB
Ush
AoB
RW1
RW2
WL
OMF
Kt
ZS
LMF
RIR
NH
PC
KJ
RC
6_iteration_1_edges
perepel
CW
RBB
Ush
YC
AoB
OMF
RW2
WL
RW1
ZS
Kt
KJ
RC
LMF
PC
RIR
NH
Pm
AS
UshF
7_iteration_1_edges
perepel
CW
UshF
AS
Pm
Ush
RBB
YC
OMF
RW2
WL
RW1
AoB
PC
LMF
NH
RIR
RC
KJ
Kt
ZS
8_iteration_1_edges
perepel
CW
Pm
AS
UshF
RIR
NH
PC
Ush
RBB
OMF
LMF
AoB
RC
KJ
YC
Kt
ZS
RW2
WL
RW1
9_iteration_1_edges
perepel
CW
UshF
AS
Pm
NH
RIR
PC
LMF
KJ
RC
Kt
ZS
YC
Ush
RBB
AoB
OMF
RW2
RW1
WL
10_iteration_1_edges
perepel
CW
UshF
AS
Pm
AoB
OMF
RW2
WL
RW1
YC
RBB
Ush
PC
LMF
RIR
NH
KJ
RC
Kt
ZS
11_iteration_1_edges
AoB
NH
RIR
LMF
PC
KJ
ZS
RC
Kt
CW
AS
Pm
UshF
YC
RBB
Ush
OMF
RW2
WL
RW1
perepel
12_iteration_1_edges
perepel
CW
UshF
AS
Pm
AoB
LMF
Ush
RBB
YC
RW2
WL
RW1
OMF
KJ
RC
ZS
Kt
PC
NH
RIR
Migration weight
0.5
0
10 s.e.
0.00
0.05
0.10
0.15
0.20
0.25
Drift parameter

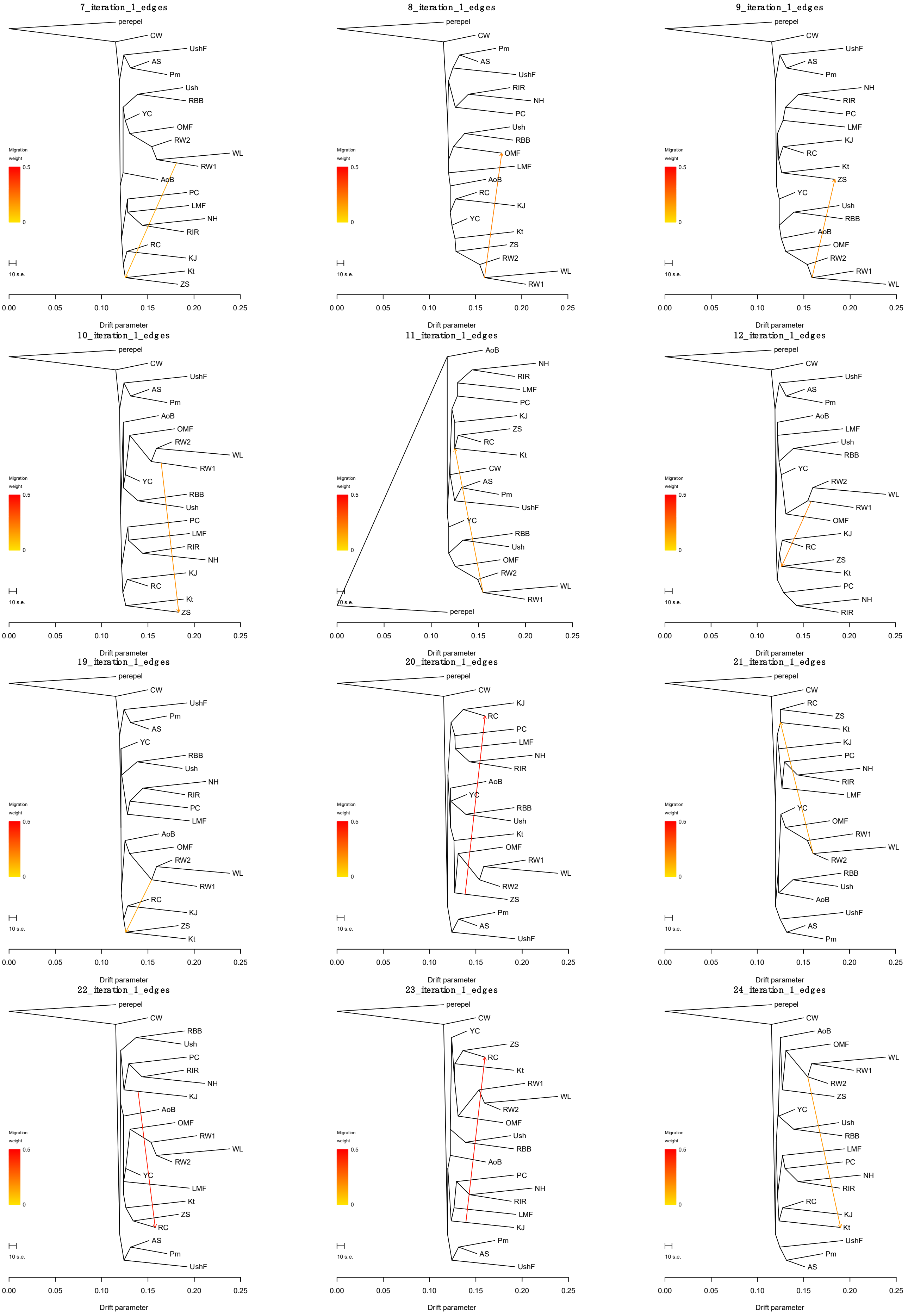
7_iteration_1_edges
perepel
CW
UshF
AS
Pm
Ush
RBB
YC
OMF
RW2
WL
RW1
AoB
PC
LMF
NH
RIR
RC
KJ
Kt
ZS
Migration weight
0.5
0
10 s.e.
0.00
0.05
0.10
0.15
0.20
0.25
Drift parameter
8_iteration_1_edges
9_iteration_1_edges
10_iteration_1_edges
11_iteration_1_edges
12_iteration_1_edges
19_iteration_1_edges
20_iteration_1_edges
21_iteration_1_edges
22_iteration_1_edges
23_iteration_1_edges
24_iteration_1_edges

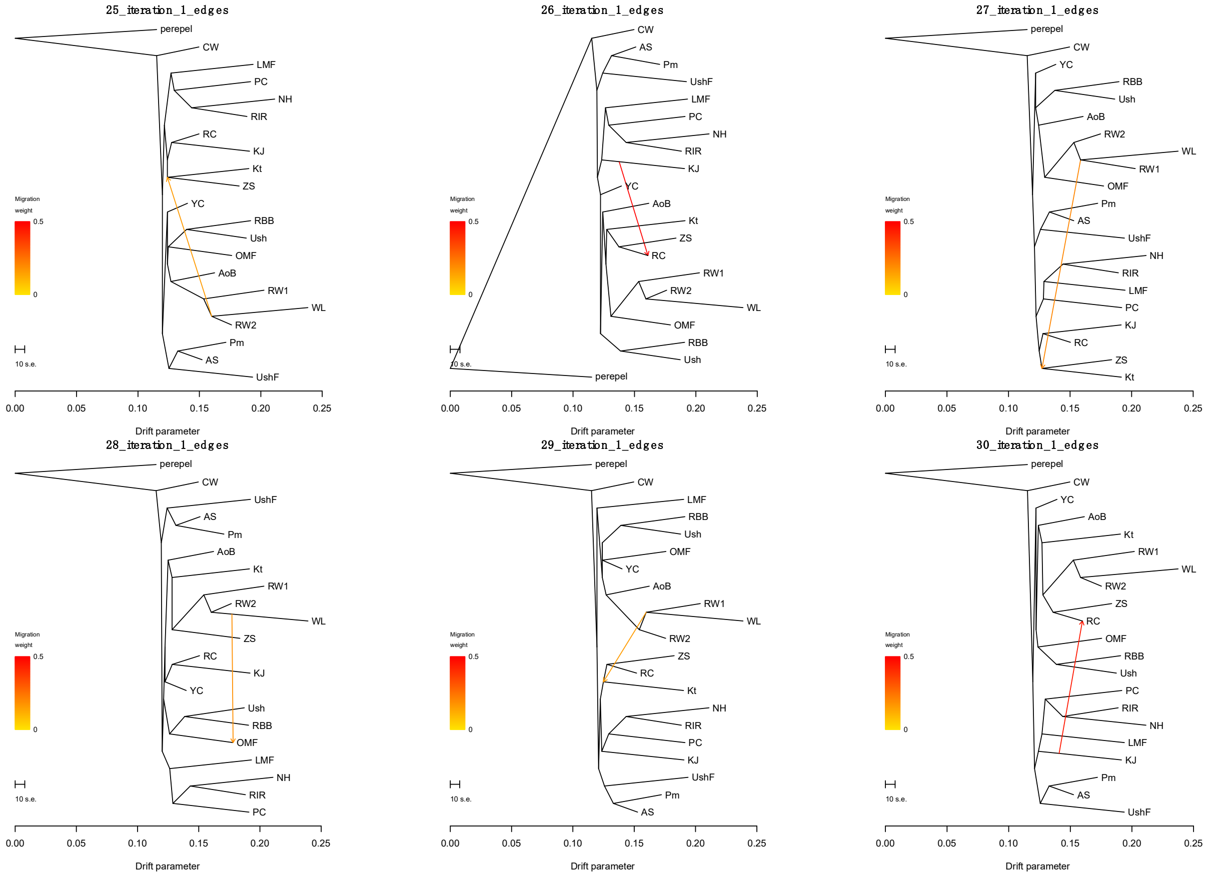

Supplementary Figure S10A. Assessment of the degree of divergence and the level of gene flow between the studied breeds. Rooted maximum likelihood trees with no migration event. Common quail (*C. coturnix*) was used as an outgroup; cut length 10 s.e. corresponds to ten times the average standard error (s.e.) estimated from the sample covariance matrix. Breed codes: AoB, Australorp Black; AS, Adler Silver; CW, Cornish White; KJ, Kuchino Jubilee; Kt, Kotlyarevsky; LMF, Leningrad Mille Fleur; NH, New Hampshire; OMF, Orloff Mille Fleur; PC, Poltava Clay; Pm, Pervomai; RBB, Russian Black Bearded; RC, Russian Crested; RIR, Rhode Island Red; RW1, Russian White (ARPRTI); RW2, Russian White (RRIFAGB); Ush, Ushanka; UshF, Ushanka Foot-feathered; WL, White Leghorn; YC, Yurlov Crower; ZS, Zagorsk Salmon.

B

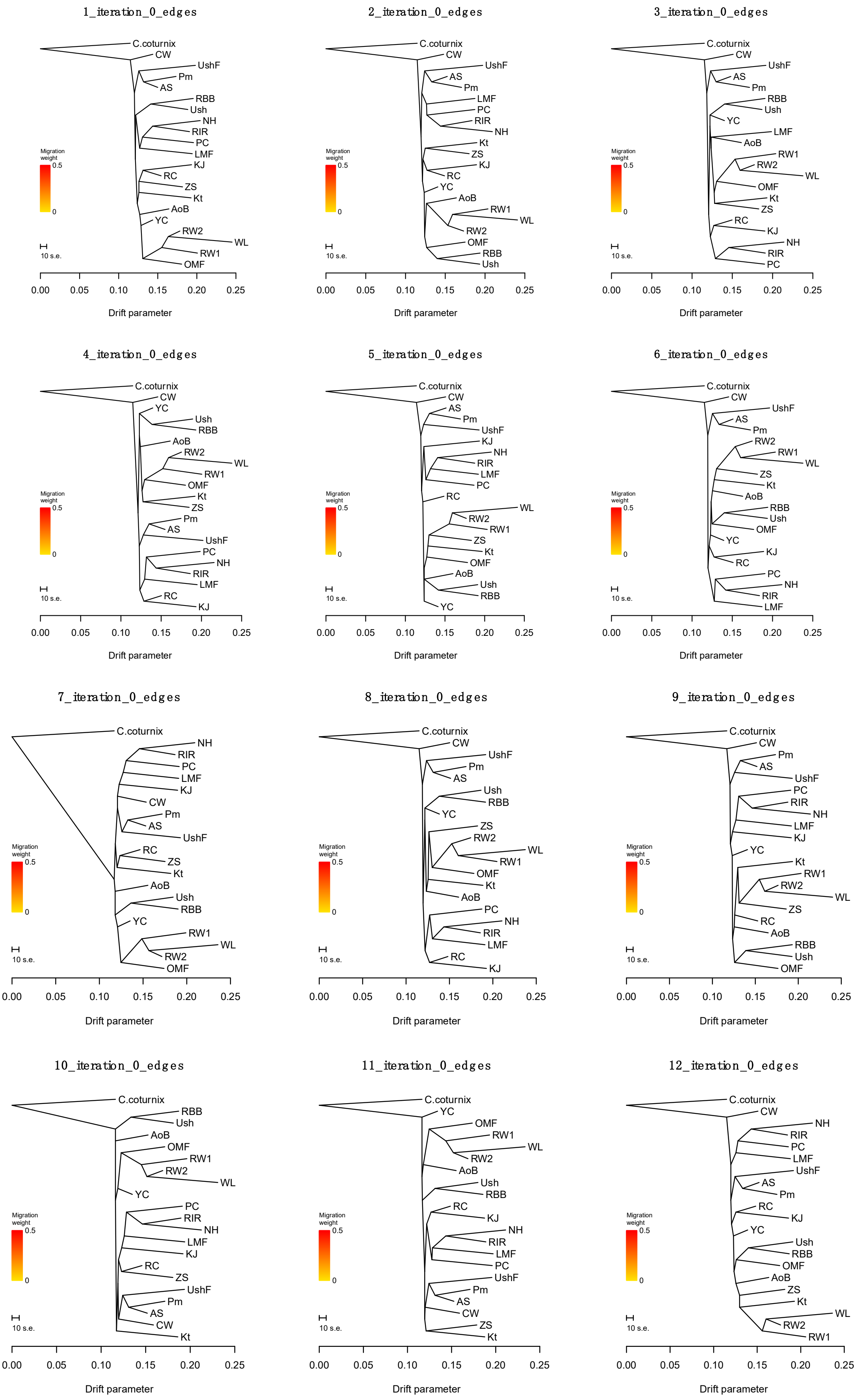
1_iteration_0_edges
C.coturnix
CW
UshF
Pm
AS
RBB
Ush
NH
RIR
PC
LMF
KJ
RC
ZS
Kt
AoB
YC
RW2
WL
RW1
OMF
Migration weight
0.5
0
10 s.e.
0.00
0.05
0.10
0.15
0.20
0.25
Drift parameter
2_iteration_0_edges
C.coturnix
CW
UshF
AS
Pm
LMF
PC
RIR
NH
Kt
ZS
KJ
RC
YC
AoB
RW1
WL
RW2
OMF
RBB
Ush
Migration weight
0.5
0
10 s.e.
0.00
0.05
0.10
0.15
0.20
0.25
Drift parameter
3_iteration_0_edges
C.coturnix
CW
UshF
AS
Pm
RBB
Ush
YC
LMF
AoB
RW1
RW2
WL
OMF
Kt
ZS
RC
KJ
NH
RIR
PC
Migration weight
0.5
0
10 s.e.
0.00
0.05
0.10
0.15
0.20
0.25
Drift parameter
4_iteration_0_edges
C.coturnix
CW
YC
Ush
RBB
AoB
RW2
WL
RW1
OMF
Kt
ZS
Pm
AS
UshF
PC
NH
RIR
LMF
RC
KJ
Migration weight
0.5
0
10 s.e.
0.00
0.05
0.10
0.15
0.20
0.25
Drift parameter
5_iteration_0_edges
C.coturnix
CW
AS
Pm
UshF
KJ
NH
RIR
LMF
PC
RC
WL
RW2
RW1
ZS
Kt
OMF
AoB
Ush
RBB
YC
Migration weight
0.5
0
10 s.e.
0.00
0.05
0.10
0.15
0.20
0.25
Drift parameter
6_iteration_0_edges
C.coturnix
CW
UshF
AS
Pm
RW2
RW1
WL
ZS
Kt
AoB
RBB
Ush
OMF
YC
KJ
RC
PC
NH
RIR
LMF
Migration weight
0.5
0
10 s.e.
0.00
0.05
0.10
0.15
0.20
0.25
Drift parameter
7_iteration_0_edges
C.coturnix
NH
RIR
PC
LMF
KJ
CW
Pm
AS
UshF
RC
ZS
Kt
AoB
Ush
RBB
YC
RW1
WL
RW2
OMF
Migration weight
0.5
0
10 s.e.
0.00
0.05
0.10
0.15
0.20
0.25
Drift parameter
8_iteration_0_edges
C.coturnix
CW
UshF
Pm
AS
Ush
RBB
YC
ZS
RW2
WL
RW1
OMF
Kt
AoB
PC
NH
RIR
LMF
RC
KJ
Migration weight
0.5
0
10 s.e.
0.00
0.05
0.10
0.15
0.20
0.25
Drift parameter
9_iteration_0_edges
C.coturnix
CW
Pm
AS
UshF
PC
RIR
NH
LMF
KJ
YC
Kt
RW1
RW2
WL
ZS
RC
AoB
RBB
Ush
OMF
Migration weight
0.5
0
10 s.e.
0.00
0.05
0.10
0.15
0.20
0.25
Drift parameter
10_iteration_0_edges
C.coturnix
RBB
Ush
AoB
OMF
RW1
RW2
WL
YC
PC
RIR
NH
LMF
KJ
RC
ZS
UshF
Pm
AS
CW
Kt
Migration weight
0.5
0
10 s.e.
0.00
0.05
0.10
0.15
0.20
0.25
Drift parameter
11_iteration_0_edges
C.coturnix
YC
OMF
RW1
WL
RW2
AoB
Ush
RBB
RC
KJ
NH
RIR
LMF
PC
UshF
Pm
AS
CW
ZS
Kt
Migration weight
0.5
0
10 s.e.
0.00
0.05
0.10
0.15
0.20
0.25
Drift parameter
12_iteration_0_edges
C.coturnix
CW
NH
RIR
PC
LMF
UshF
AS
Pm
RC
KJ
YC
Ush
RBB
OMF
AoB
ZS
Kt
WL
RW2
RW1
Migration weight
0.5
0
10 s.e.
0.00
0.05
0.10
0.15
0.20
0.25
Drift parameter

13_iteration_0_edges

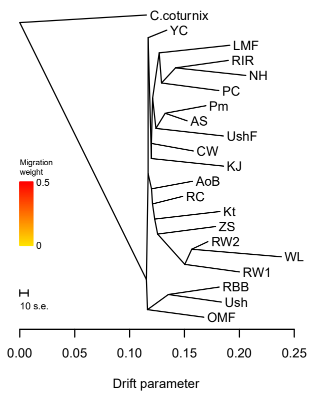

14_iteration_0_edges

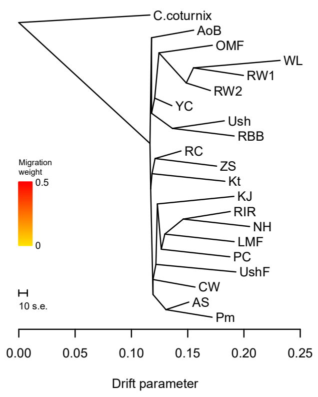

15_iteration_0_edges

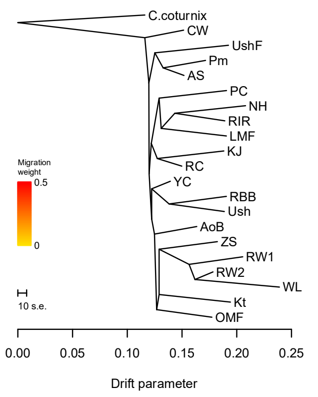

16_iteration_0_edges

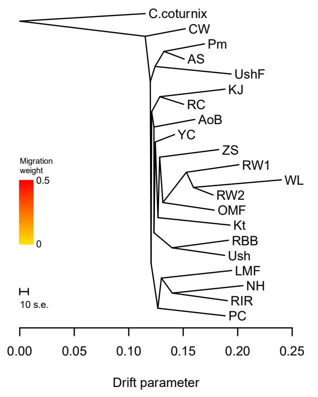

17_iteration_0_edges

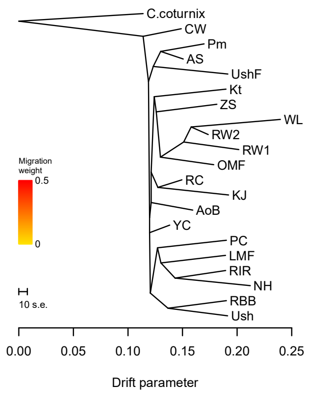

18_iteration_0_edges

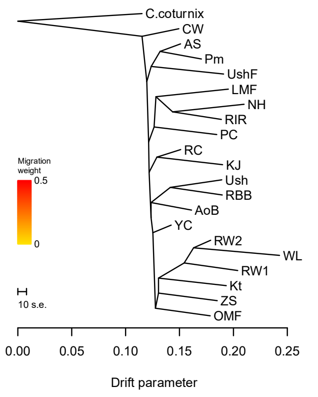

19_iteration_0_edges

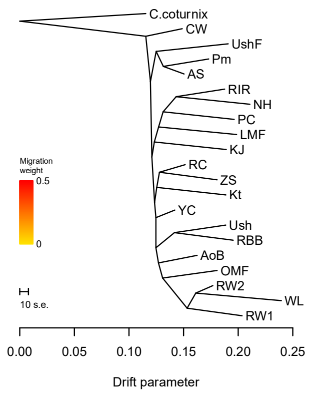

20_iteration_0_edges

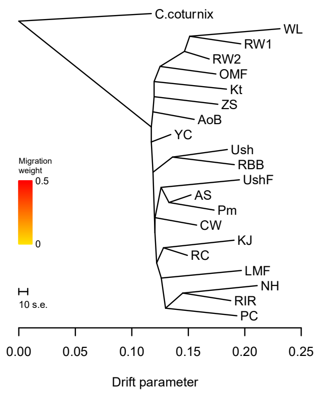

21_iteration_0_edges

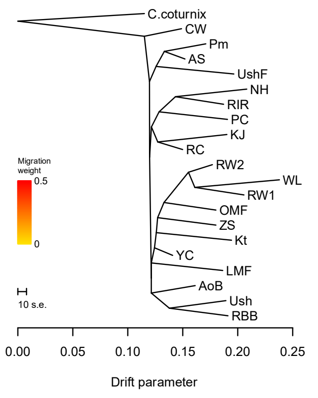

22_iteration_0_edges

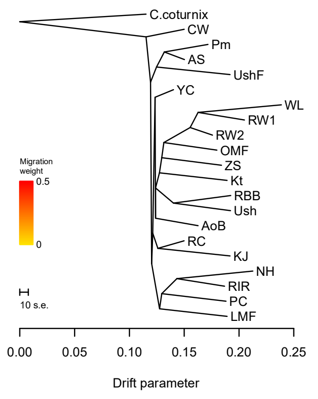

23_iteration_0_edges

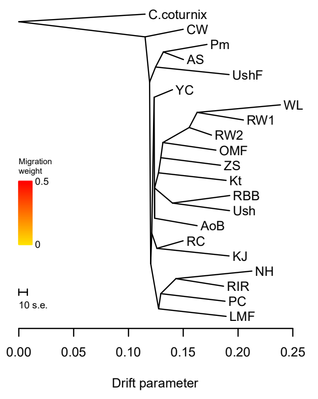

24_iteration_0_edges

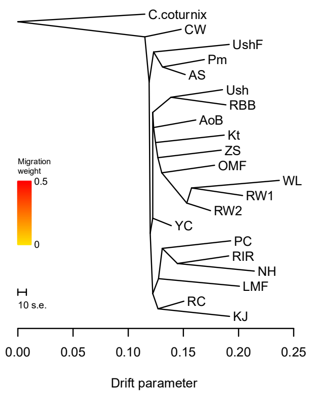

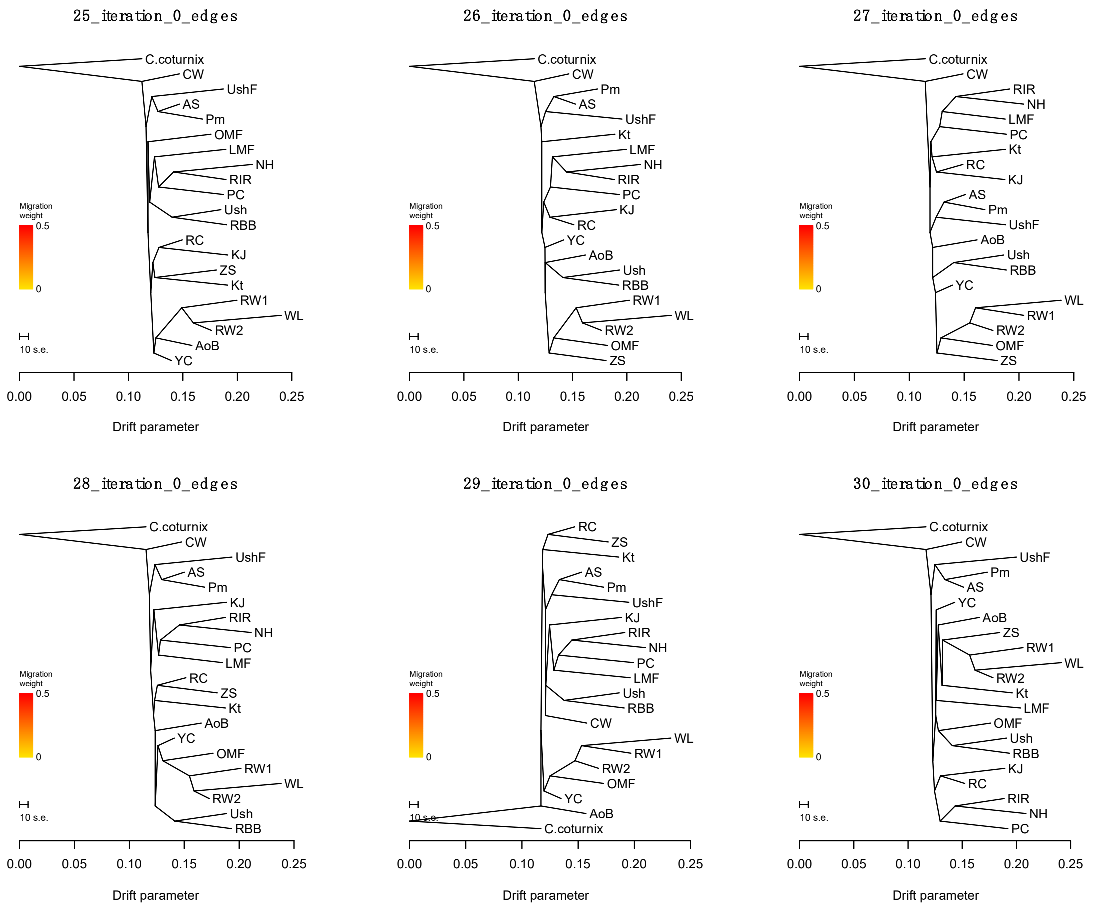

Supplementary Figure S10B. Same as Supplementary Figure S10A, except for rooted maximum likelihood trees with no migration event. Breed codes: AoB, Australorp Black; AS, Adler Silver; CW, Cornish White; KJ, Kuchino Jubilee; Kt, Kotlyarevsky; LMF, Leningrad Mille Fleur; NH, New Hampshire; OMF, Orloff Mille Fleur; PC, Poltava Clay; Pm, Pervomai; RBB, Russian Black Bearded; RC, Russian Crested; RIR, Rhode Island Red; RW1, Russian White (ARPRTI); RW2, Russian White (RRIFAGB); Ush, Ushanka; UshF, Ushanka Foot-feathered; WL, White Leghorn; YC, Yurlov Crower; ZS, Zagorsk Salmon. Outgroup: *C. coturnix*, quail.

C

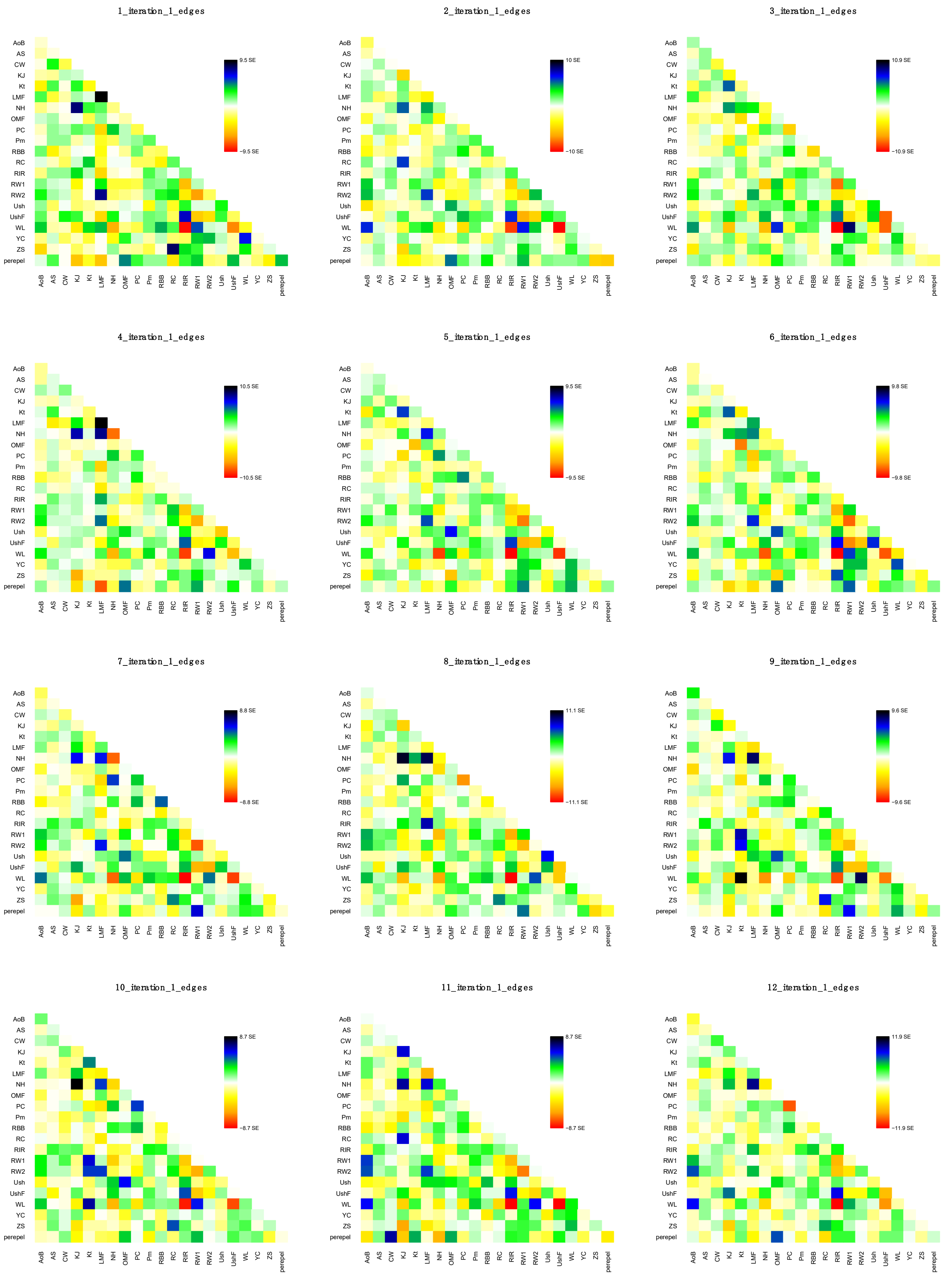
1_iteration_1_edges
9.5 SE
−9.5 SE
2_iteration_1_edges
10 SE
−10 SE
3_iteration_1_edges
10.9 SE
−10.9 SE
4_iteration_1_edges
10.5 SE
−10.5 SE
5_iteration_1_edges
9.5 SE
−9.5 SE
6_iteration_1_edges
9.8 SE
−9.8 SE
7_iteration_1_edges
8.8 SE
−8.8 SE
8_iteration_1_edges
11.1 SE
−11.1 SE
9_iteration_1_edges
9.6 SE
−9.6 SE
10_iteration_1_edges
8.7 SE
−8.7 SE
11_iteration_1_edges
8.7 SE
−8.7 SE
12_iteration_1_edges
11.9 SE
−11.9 SE
AoB
AS
CW
KJ
Kt
LMF
NH
OMF
PC
Pm
RBB
RC
RIR
RW1
RW2
Ush
UshF
WL
YC
ZS
perepel

13_iteration_1_edges

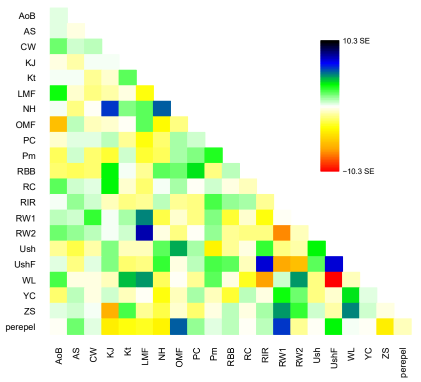

14_iteration_1_edges

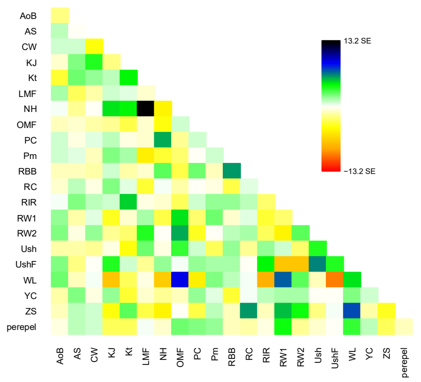

15_iteration_1_edges

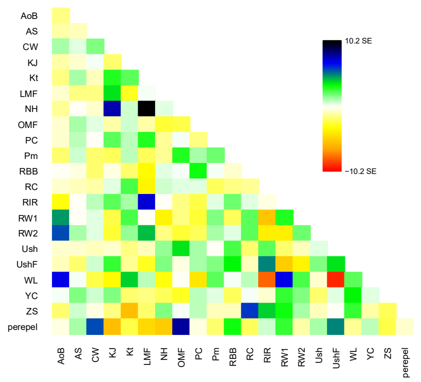

16_iteration_1_edges

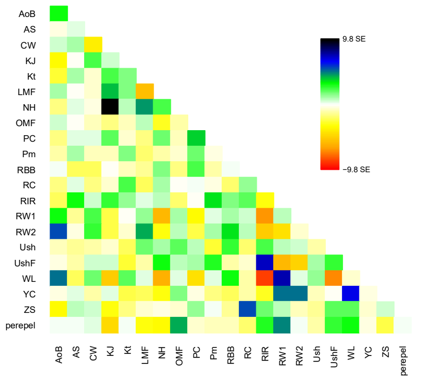

17_iteration_1_edges

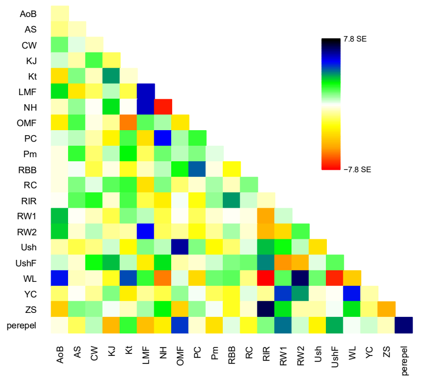

18_iteration_1_edges

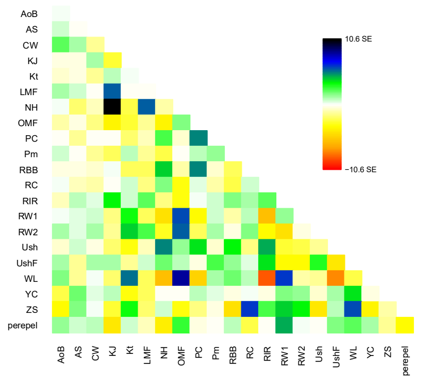

19_iteration_1_edges

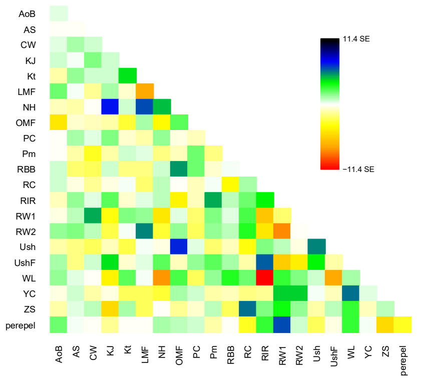

20_iteration_1_edges

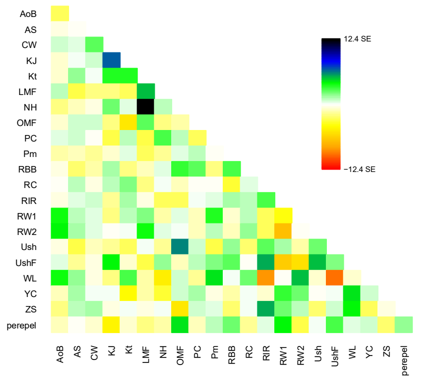

21_iteration_1_edges

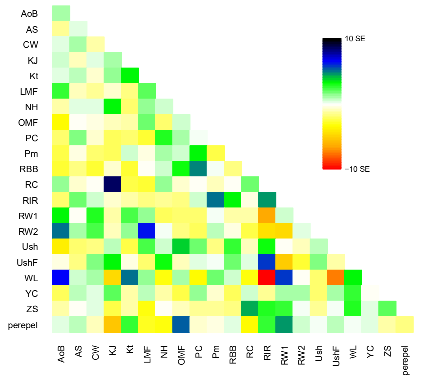

22_iteration_1_edges

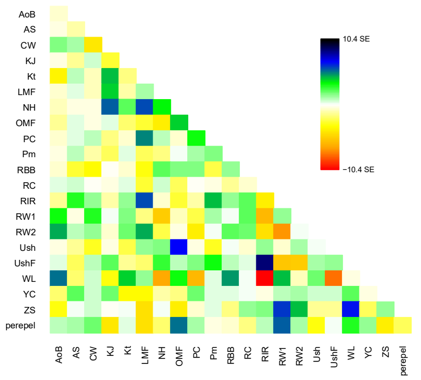

23_iteration_1_edges

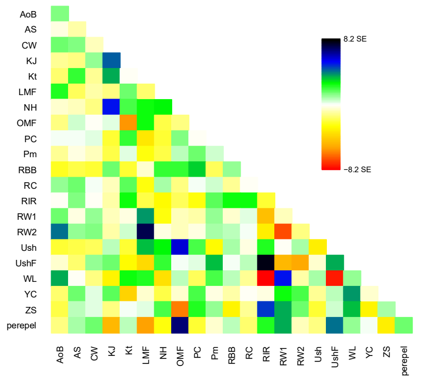

24_iteration_1_edges

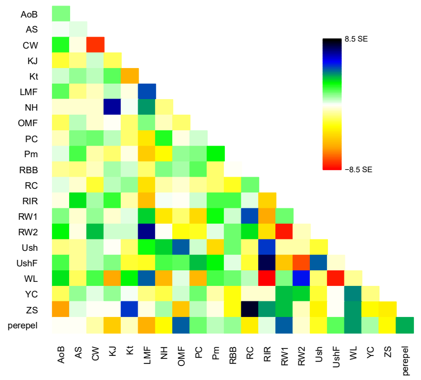

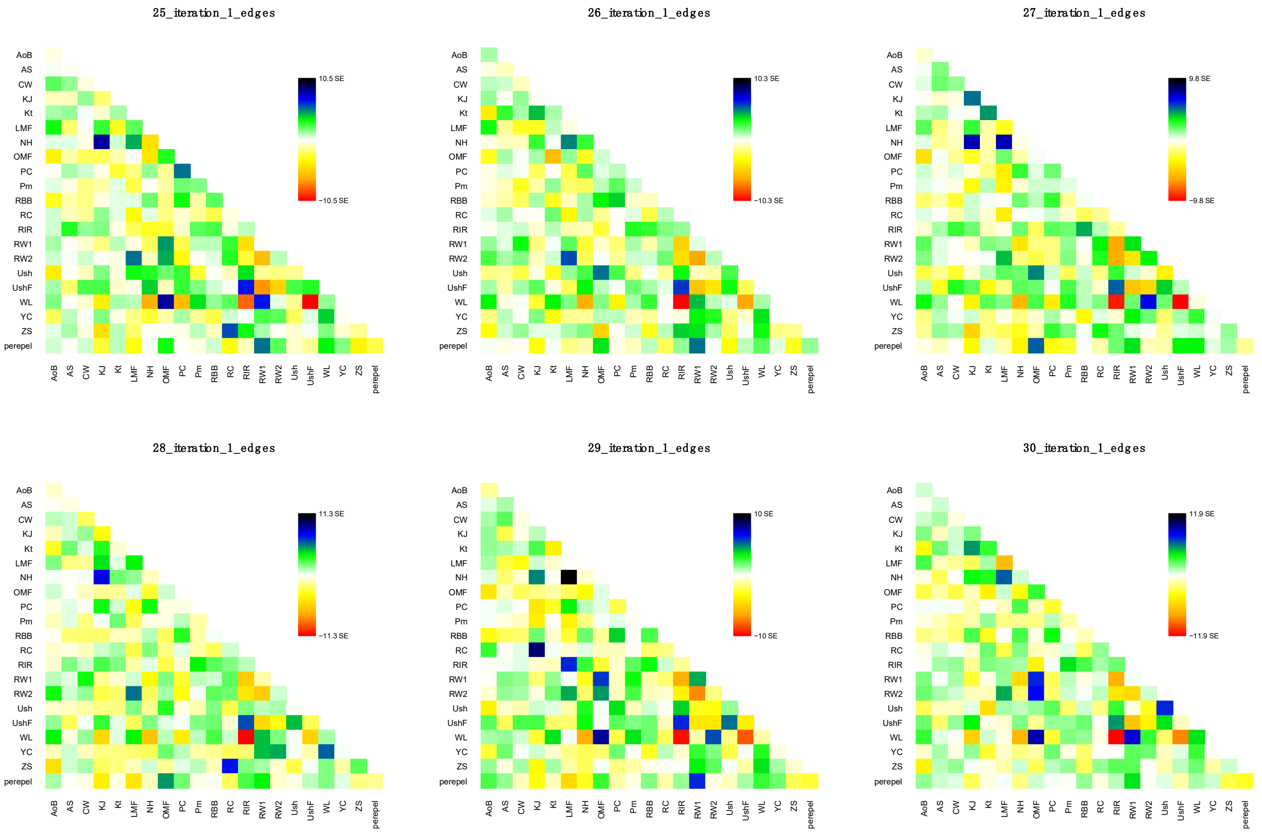

Supplementary Figure S10C. Assessment of the degree of divergence and the level of gene flow between the studied breeds. Residual matrix derived from the TreeMix analysis for a single migration event, expressed as the number of standard error deviations for the observations in the respective breeds. Breed codes: AoB, Australorp Black; AS, Adler Silver; CW, Cornish White; KJ, Kuchino Jubilee; Kt, Kotlyarevsky; LMF, Leningrad Mille Fleur; NH, New Hampshire; OMF, Orloff Mille Fleur; PC, Poltava Clay; Pm, Pervomai; RBB, Russian Black Bearded; RC, Russian Crested; RIR, Rhode Island Red; RW1, Russian White (ARPRTI); RW2, Russian White (RRIFAGB); Ush, Ushanka; UshF, Ushanka Foot-feathered; WL, White Leghorn; YC, Yurlov Crower; ZS, Zagorsk Salmon. Outgroup: perepel, quail (*C. coturnix*).

D

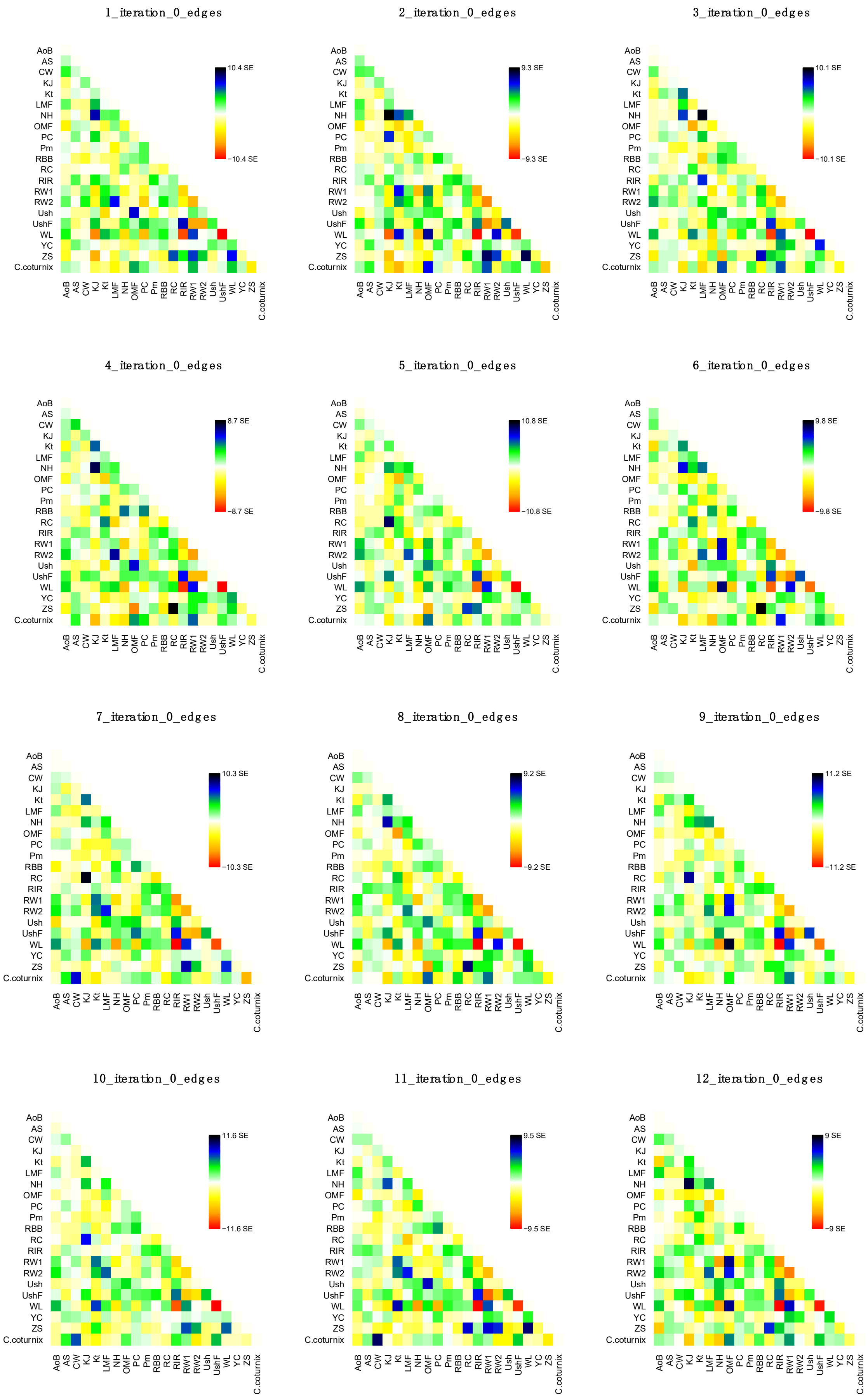

1_iteration_0_edges
10.4 SE
−10.4 SE
2_iteration_0_edges
9.3 SE
−9.3 SE
3_iteration_0_edges
10.1 SE
−10.1 SE
4_iteration_0_edges
8.7 SE
−8.7 SE
5_iteration_0_edges
10.8 SE
−10.8 SE
6_iteration_0_edges
9.8 SE
−9.8 SE
7_iteration_0_edges
10.3 SE
−10.3 SE
8_iteration_0_edges
9.2 SE
−9.2 SE
9_iteration_0_edges
11.2 SE
−11.2 SE
10_iteration_0_edges
11.6 SE
−11.6 SE
11_iteration_0_edges
9.5 SE
−9.5 SE
12_iteration_0_edges
9 SE
−9 SE
AoB
AS
CW
KJ
Kt
LMF
NH
OMF
PC
Pm
RBB
RC
RIR
RW1
RW2
Ush
UshF
WL
YC
ZS
C.coturnix

13_iteration_0_edges

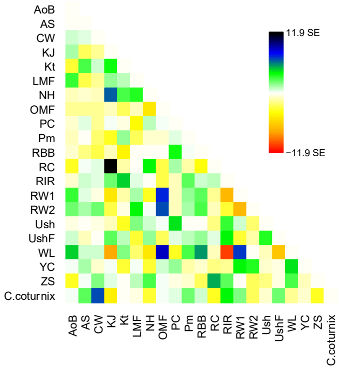

14_iteration_0_edges

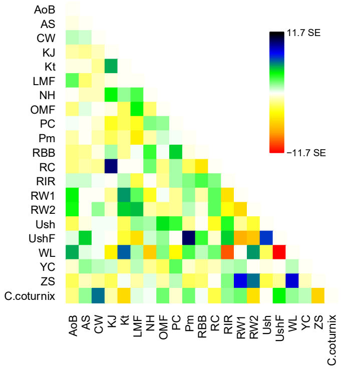

15_iteration_0_edges

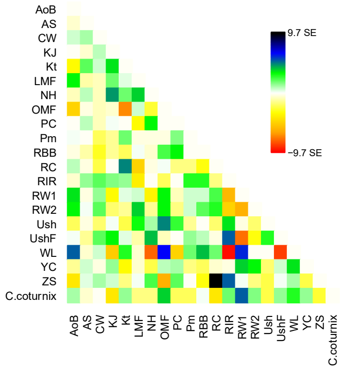

16_iteration_0_edges

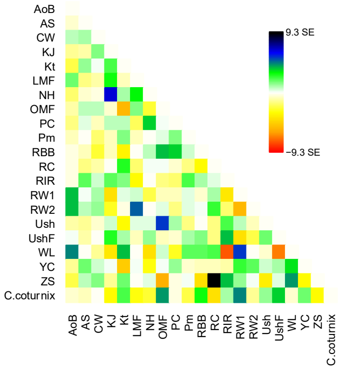

17_iteration_0_edges

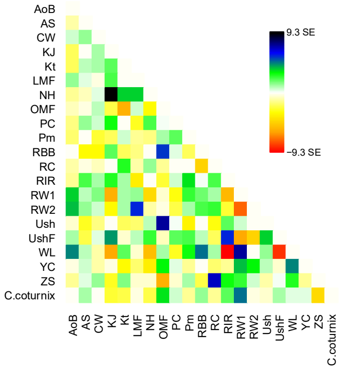

18_iteration_0_edges

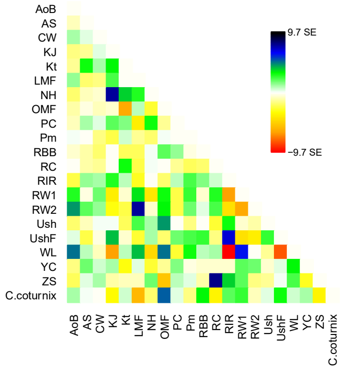

19_iteration_0_edges

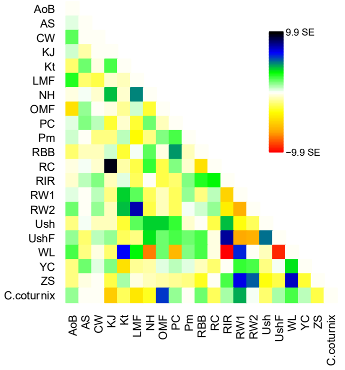

20_iteration_0_edges

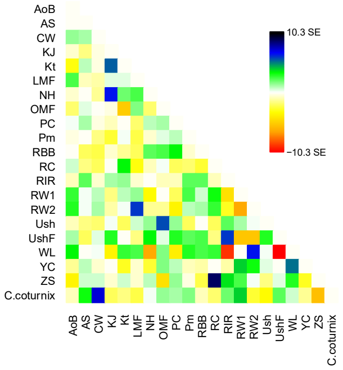

21_iteration_0_edges

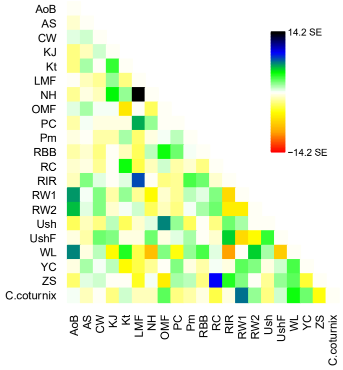

22_iteration_0_edges

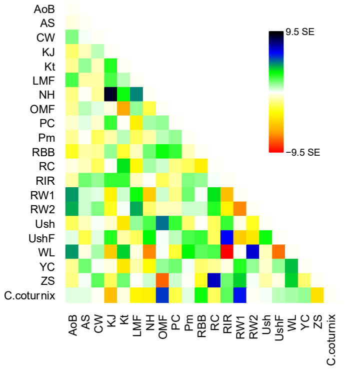

23_iteration_0_edges

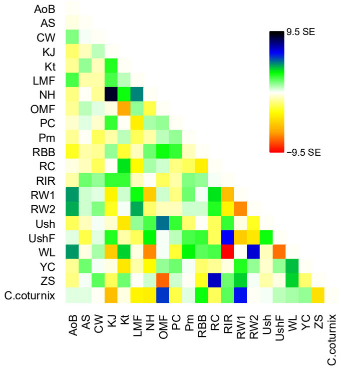

24_iteration_0_edges

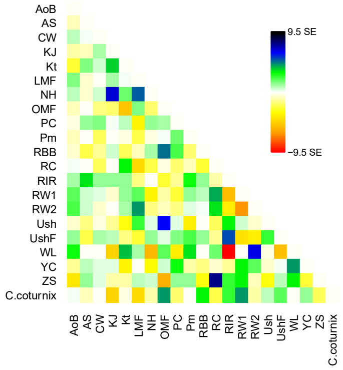

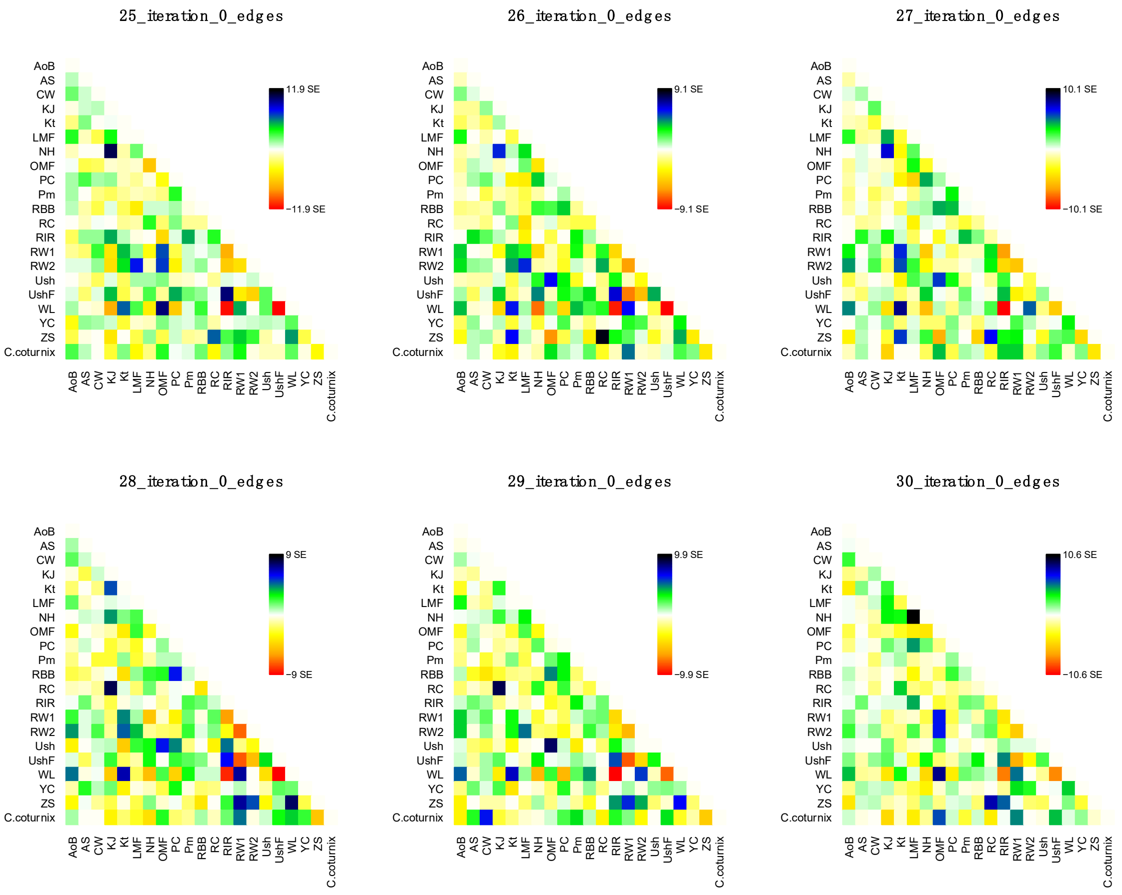

Supplementary Figure S10D. Same as Supplementary Figure S10C, except for no migration events. Breed codes: AoB, Australorp Black; AS, Adler Silver; CW, Cornish White; KJ, Kuchino Jubilee; Kt, Kotlyarevsky; LMF, Leningrad Mille Fleur; NH, New Hampshire; OMF, Orloff Mille Fleur; PC, Poltava Clay; Pm, Pervomai; RBB, Russian Black Bearded; RC, Russian Crested; RIR, Rhode Island Red; RW1, Russian White (ARPRTI); RW2, Russian White (RRIFAGB); Ush, Ushanka; UshF, Ushanka Foot-feathered; WL, White Leghorn; YC, Yurlov Crower; ZS, Zagorsk Salmon. Outgroup: *C. coturnix*, quail.

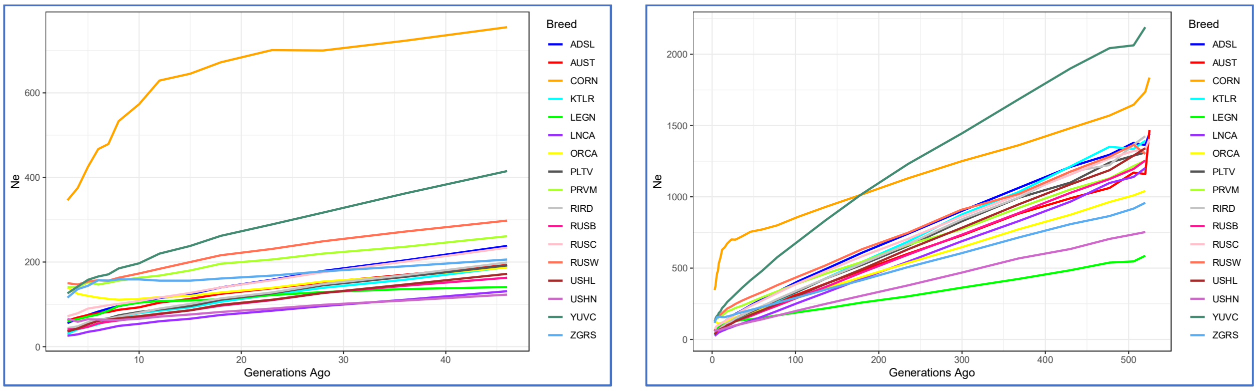

A **B**

Supplementary Figure S11. Dynamics of changes in the effective population size (N_e). This was plotted using the *SNeP* program [78] for the 17 studied breeds (except KJ and NH) in the interval of 3–46 (**A**) and 3–525 (**B**) generations ago. Breed abbreviations: ADSL, Adler Silver; AUST, Australorp Black; CORN, Cornish White; KTLR, Kotlyarevsky; LEGN, White Leghorn; LNCA, Leningrad Mille Fleur; ORCA, Orloff Mille Fleur; PLTV, Poltava Clay; PRVM, Pervomai; RIRD, Rhode Island Red; RUSB, Russian Black Bearded; RUSC, Russian Crested; RUSW, Russian White; USHL, Ushanka Foot-feathered; USHN, Ushanka; YUVC, Yurlov Crower; ZGRS, Zagorsk Salmon.

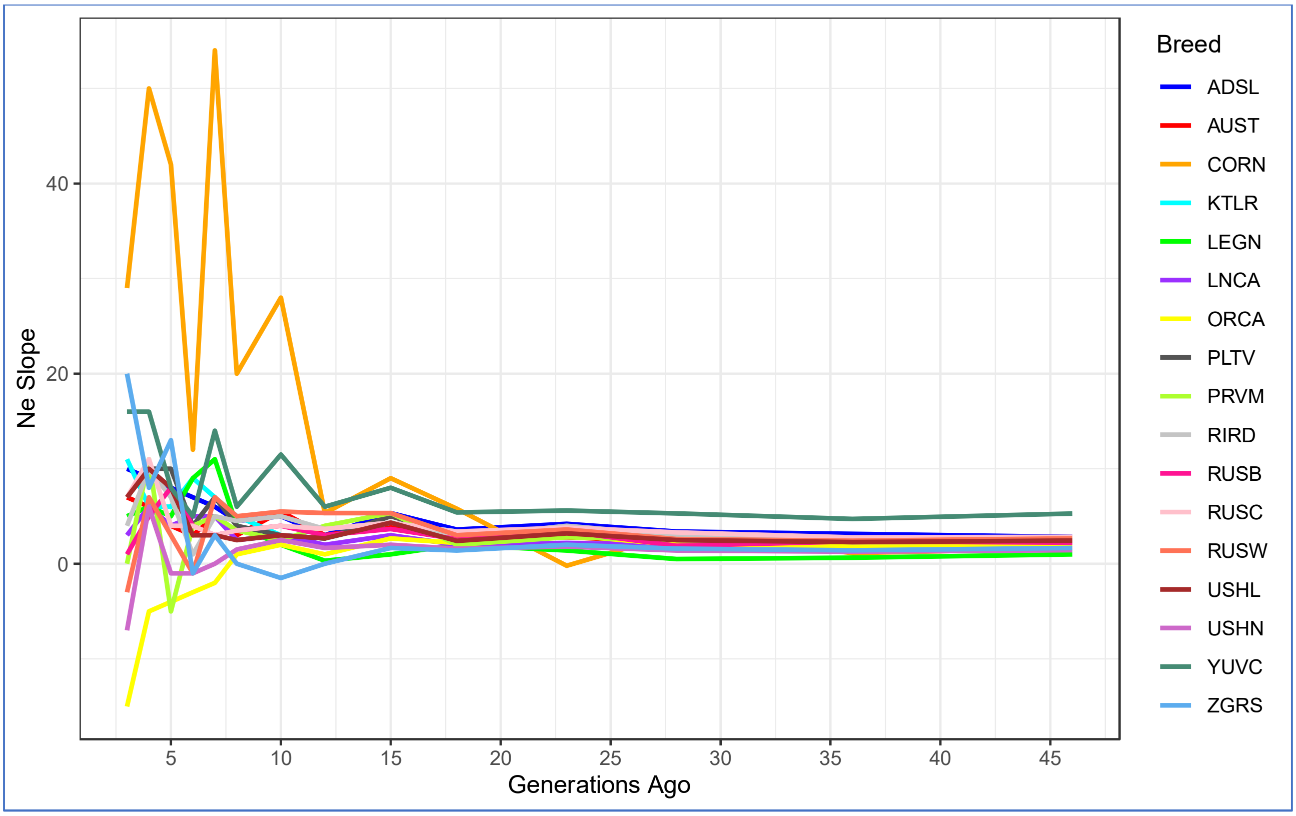

Supplementary Figure S12. Analysis of the N_e slopes [83]. These exhibit N_e changes in the 17 studied breeds (except KJ and NH) over a period of 3–46 generations ago. The constant rate of change is shown as a horizontal straight line at point 0 on the *y*-axis, while deviations above and below 0 represent, respectively, the relative increase and decrease in the changing N_e slope as compared to the previous generation. Breed abbreviations: ADSL, Adler Silver; AUST, Australorp Black; CORN, Cornish White; KTLR, Kotlyarevsky; LEGN, White Leghorn; LNCA, Leningrad Mille Fleur; ORCA, Orloff Mille Fleur; PLTV, Poltava Clay; PRVM, Pervomai; RIRD, Rhode Island Red; RUSB, Russian Black Bearded; RUSC, Russian Crested; RUSW, Russian White; USHL, Ushanka Foot-feathered; USHN, Ushanka; YUVC, Yurlov Crower; ZGRS, Zagorsk Salmon.

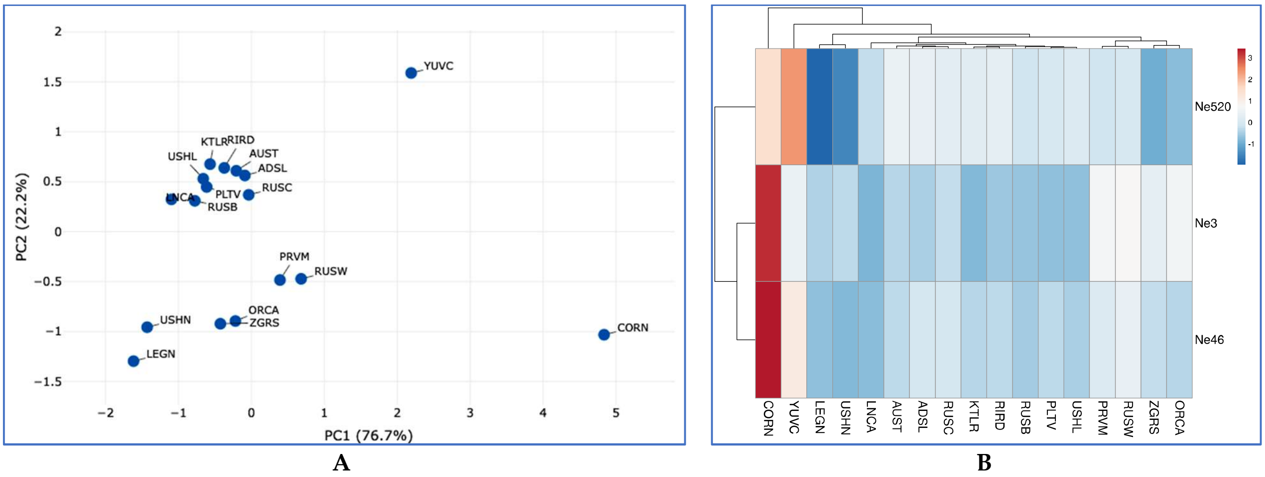

Supplementary Figure S13. Distribution of the 17 studied breeds (except KJ and NH) when combining the N_e data for 3, 46, and 520–525 generations ago. (**A**) PCA plot built using the Phantasus program [56]. Unit variance scaling is applied to the rows of the original matrix; multilevel singular value decomposition (SVD) with imputation was used to calculate the principal components (PCs). The axes *x* and *y* show PC1 and PC2 that account for 76.7 and 22.2% of the total variance, respectively. (**B**) Heat map and trees obtained using the ClustVis program [64] and Euclidean distances for both rows and columns of the matrix (with the *average* option selected for the clustering method). Breed abbreviations: ADSL, Adler Silver; AUST, Australorp Black; CORN, Cornish White; KTLR, Kotlyarevsky; LEGN, White Leghorn; LNCA, Leningrad Mille Fleur; ORCA, Orloff Mille Fleur; PLTV, Poltava Clay; PRVM, Pervomai; RIRD, Rhode Island Red; RUSB, Russian Black Bearded; RUSC, Russian Crested; RUSW, Russian White; USHL, Ushanka Foot-feathered; USHN, Ushanka; YUVC, Yurlov Crower; ZGRS, Zagorsk Salmon.

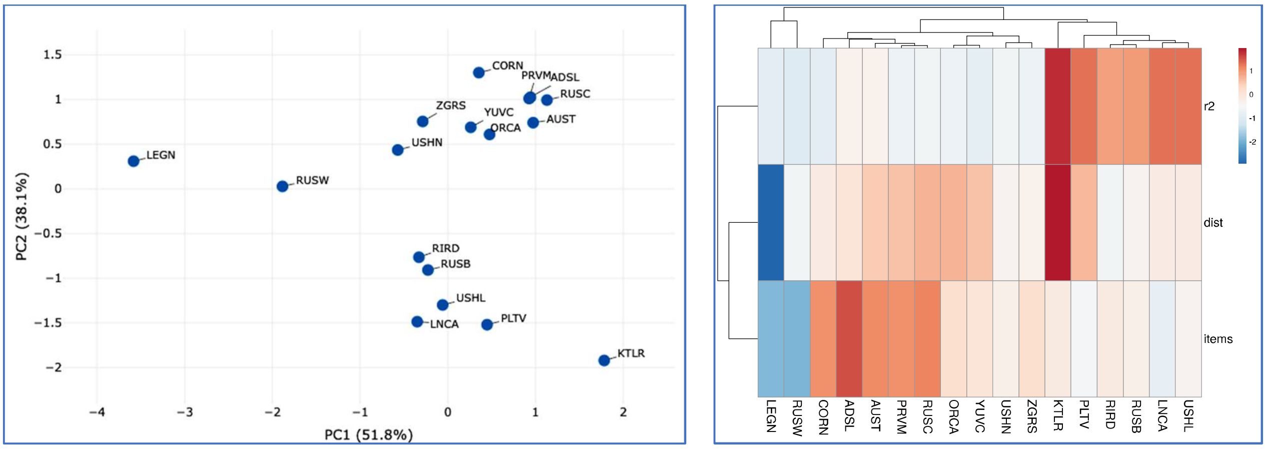

A B

Supplementary Figure S14. Distribution of the 17 studied breeds (except KJ and NH) when combining the r^2, *dist* and *items* data for 3 generations ago. (**A**) PCA plot built using the Phantasus program [56]. Unit variance scaling is applied to the rows of the original matrix; multilevel singular value decomposition (SVD) with imputation was used to calculate the principal components (PCs). The axes x and y show PC1 and PC2 that account for 51.8 and 38.1% of the total variance, respectively. (**B**) Heat map and trees obtained using the ClustVis program [64] and Euclidean distances for both rows and columns of the matrix (with the *average* option selected for the clustering method). Breed abbreviations: ADSL, Adler Silver; AUST, Australorp Black; CORN, Cornish White; KTLR, Kotlyarevsky; LEGN, White Leghorn; LNCA, Leningrad Mille Fleur; ORCA, Orloff Mille Fleur; PLTV, Poltava Clay; PRVM, Pervomai; RIRD, Rhode Island Red; RUSB, Russian Black Bearded; RUSC, Russian Crested; RUSW, Russian White; USHL, Ushanka Foot-feathered; USHN, Ushanka; YUVC, Yurlov Crower; ZGRS, Zagorsk Salmon.

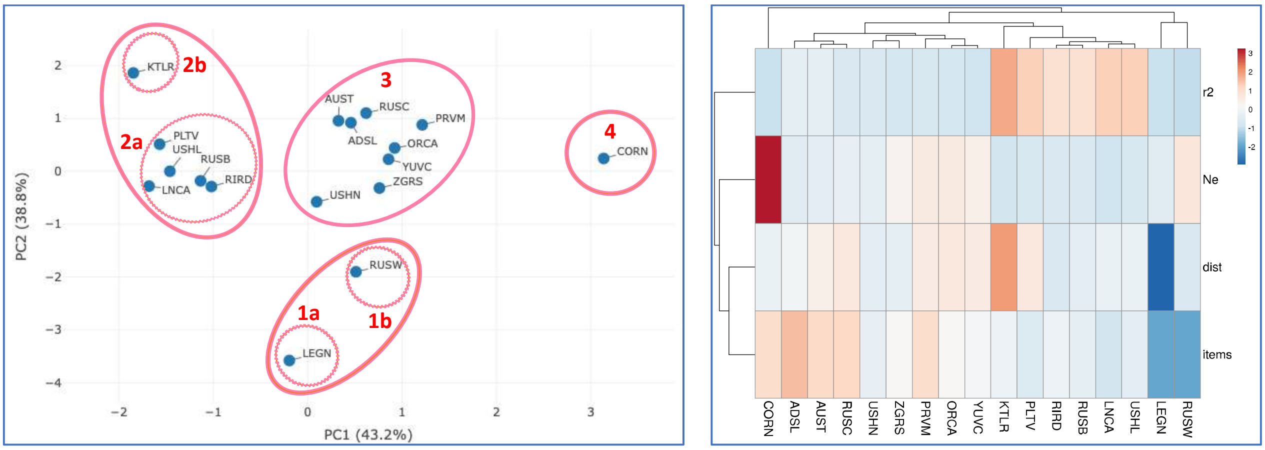

A B

Supplementary Figure S15. Distribution of the 17 studied breeds (except KJ and NH) when combining the N_e, r^2, *dist* and *items* data for 3 generations ago. (**A**) PCA plot built using the Phantasus program [56]. Unit variance scaling is applied to the rows of the original matrix; multilevel singular value decomposition (SVD) with imputation was used to calculate the principal components (PCs). The axes x and y show PC1 and PC2 that account for 43.2 and 38.8% of the total variance, respectively. N = 17 data points (breeds). (**B**) Heat map and trees obtained using the ClustVis program [64] and Euclidean distances for both rows and columns of the matrix (with the *average* option selected for the clustering method). Breed abbreviations: ADSL, Adler Silver; AUST, Australorp Black; CORN, Cornish White; KTLR, Kotlyarevsky; LEGN, White Leghorn; LNCA, Leningrad Mille Fleur; ORCA, Orloff Mille Fleur; PLTV, Poltava Clay; PRVM, Pervomai; RIRD, Rhode Island Red; RUSB, Russian Black Bearded; RUSC, Russian Crested; RUSW, Russian White; USHL, Ushanka Foot-feathered; USHN, Ushanka; YUVC, Yurlov Crower; ZGRS, Zagorsk Salmon.
