# Supplementary material for: Whole Genome Screening Procures a Holistic Hold of the Russian Chicken Gene Pool Heritage and Demographic History
Source: Biology (Basel). 2023 Jul 10;12(7):979. doi: 10.3390/biology12070979 (PMC10376169; doi:10.3390/biology12070979)
Supplement: Supplementary file 1 [file biology-12-00979-s001.zip › Suppl Figures S1¿CS8 edited.pdf]

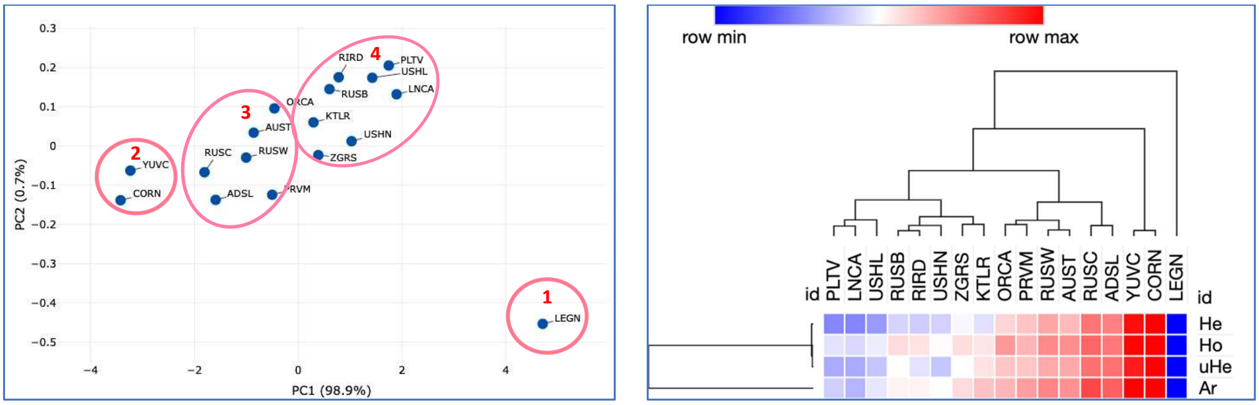

A **B**

Supplementary Figure S1. Distribution of SNP-genotyped chicken breeds into four clusters depending on their genetic variability. This was calculated from the observed (*Ho*), expected heterozygosity (*He*), unbiased expected (objectively expected) degree of heterozygosity (*uHe*) and rarefied allelic diversity (*Ar*) using the Phantasus web tool [56]. (**A**) PCA plot based on the unit variance scaling applied to the rows of the original matrix and multilevel singular value decomposition (SVD) with imputation used to calculate the principal components (PC). The *x* and *y* axes represent PC1 and PC2 that account for 98.9 and 0.7% of the total variance, respectively. (**B**) Hierarchical clustering tree using Euclidean distances. See also explanations in the text. Breed abbreviations: ADSL, Adler Silver; AUST, Australorp Black; CORN, Cornish White; KTLR, Kotlyarevsky; LEGN, White Leghorn; LNCA, Leningrad Mille Fleur; ORCA, Orloff Mille Fleur; PLTV, Poltava Clay; PRVM, Pervomai; RIRD, Rhode Island Red; RUSB, Russian Black Bearded; RUSC, Russian Crested; RUSW, Russian White; USHL, Ushanka Foot-feathered; USHN, Ushanka; YUVC, Yurlov Crower; ZGRS, Zagorsk Salmon.

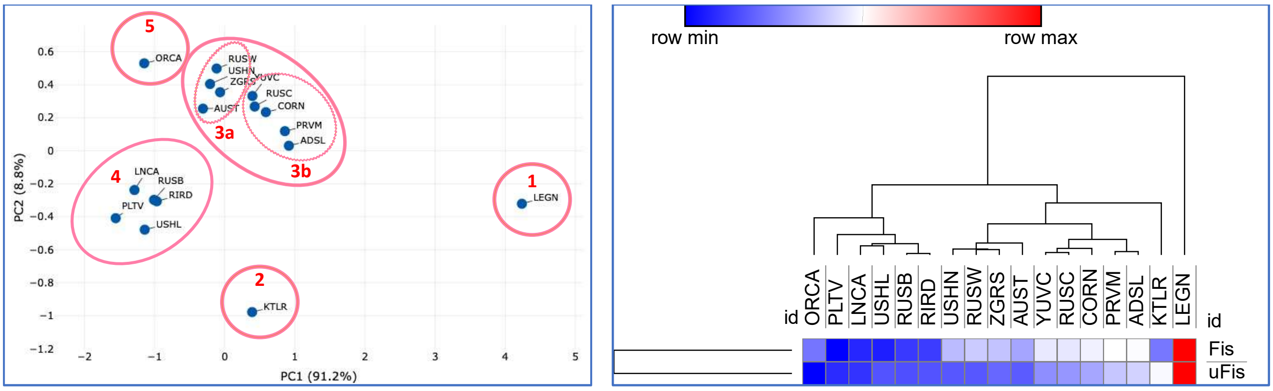

A **B**

Supplementary Figure S2. Distribution of SNP-genotyped chicken breeds into five clusters depending on their genetic structure. This was inferred from the inbreeding (*Fis*) and unbiased inbreeding (*uFis*) coefficients using the Phantasus web tool [56]. (**A**) PCA plot based on the unit variance scaling applied to the rows of the original matrix and multilevel singular value decomposition (SVD) with imputation used to calculate the principal components (PC). The *x* and *y* axes represent PC1 and PC2 that account for 91.2 and 8.8% of the total variance, respectively. (**B**) Hierarchical clustering tree using Euclidean distances. See also explanations in the text. Breed abbreviations: ADSL, Adler Silver; AUST, Australorp Black; CORN, Cornish White; KTLR, Kotlyarevsky; LEGN, White Leghorn; LNCA, Leningrad Mille Fleur; ORCA, Orloff Mille Fleur; PLTV, Poltava Clay; PRVM, Pervomai; RIRD, Rhode Island Red; RUSB, Russian Black Bearded; RUSC, Russian Crested; RUSW, Russian White; USHL, Ushanka Foot-feathered; USHN, Ushanka; YUVC, Yurlov Crower; ZGRS, Zagorsk Salmon.

A

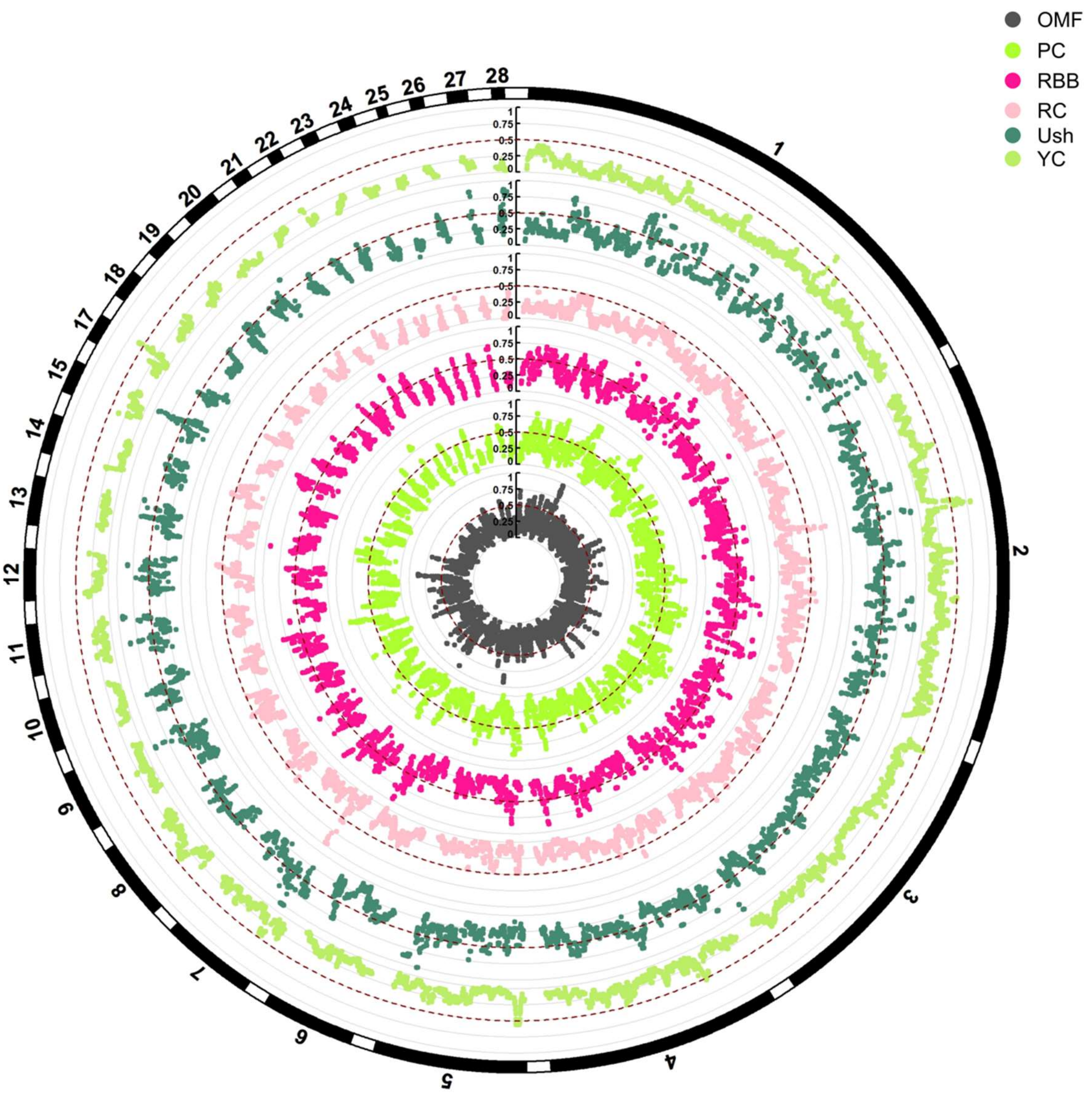
OMF
PC
RBB
RC
Ush
YC
1
2
3
4
5
6
7
8
9
10
11
12
13
14
15
17
18
19
20
21
22
23
24
25
26
27
28
0
0.25
0.5
0.75
1

B

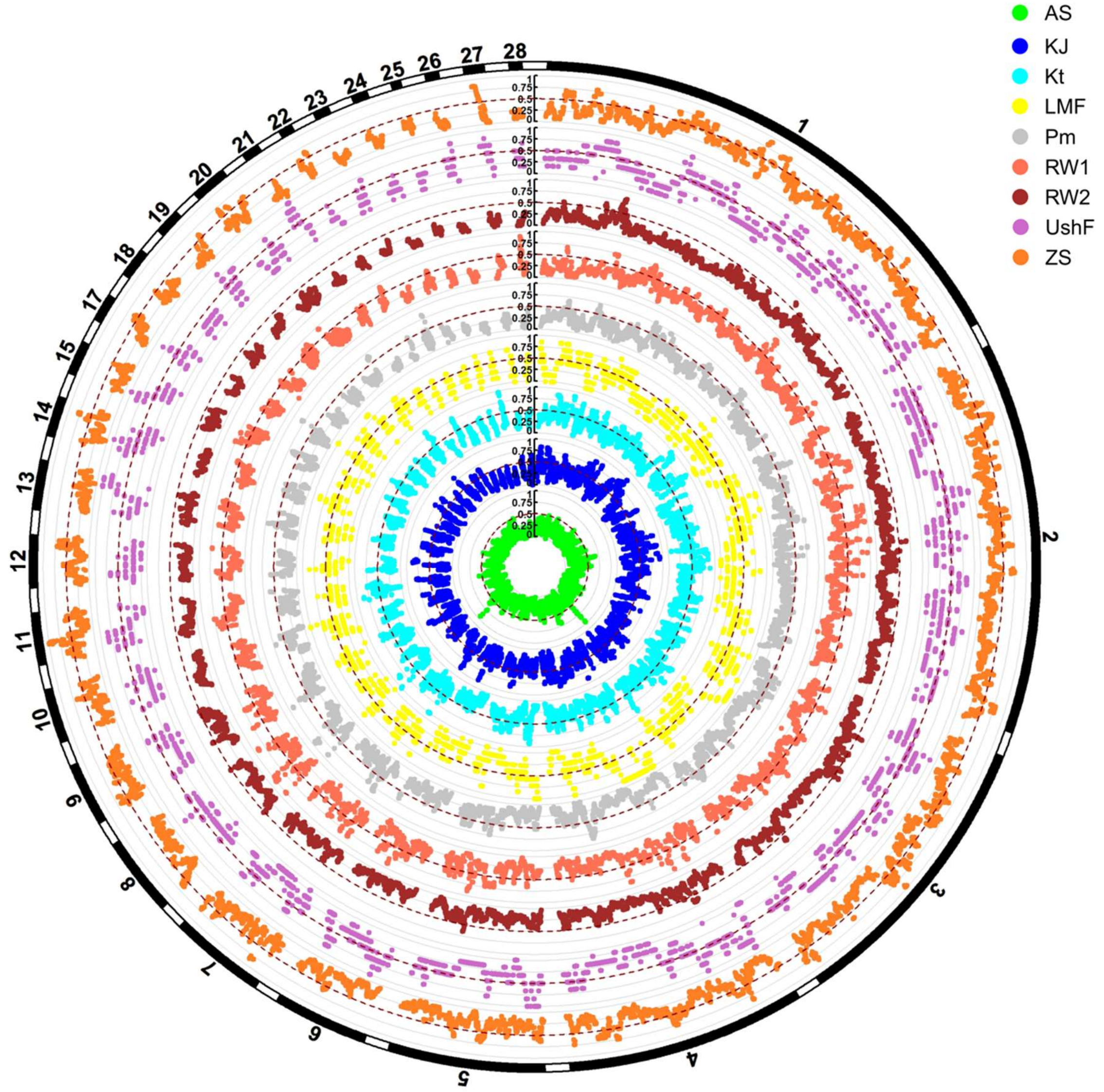

AS
KJ
Kt
LMF
Pm
RW1
RW2
UshF
ZS
1
2
3
4
5
6
7
8
9
10
11
12
13
14
15
17
18
19
20
21
22
23
24
25
26
27
28
1
0.75
0.5
0.25
0

C

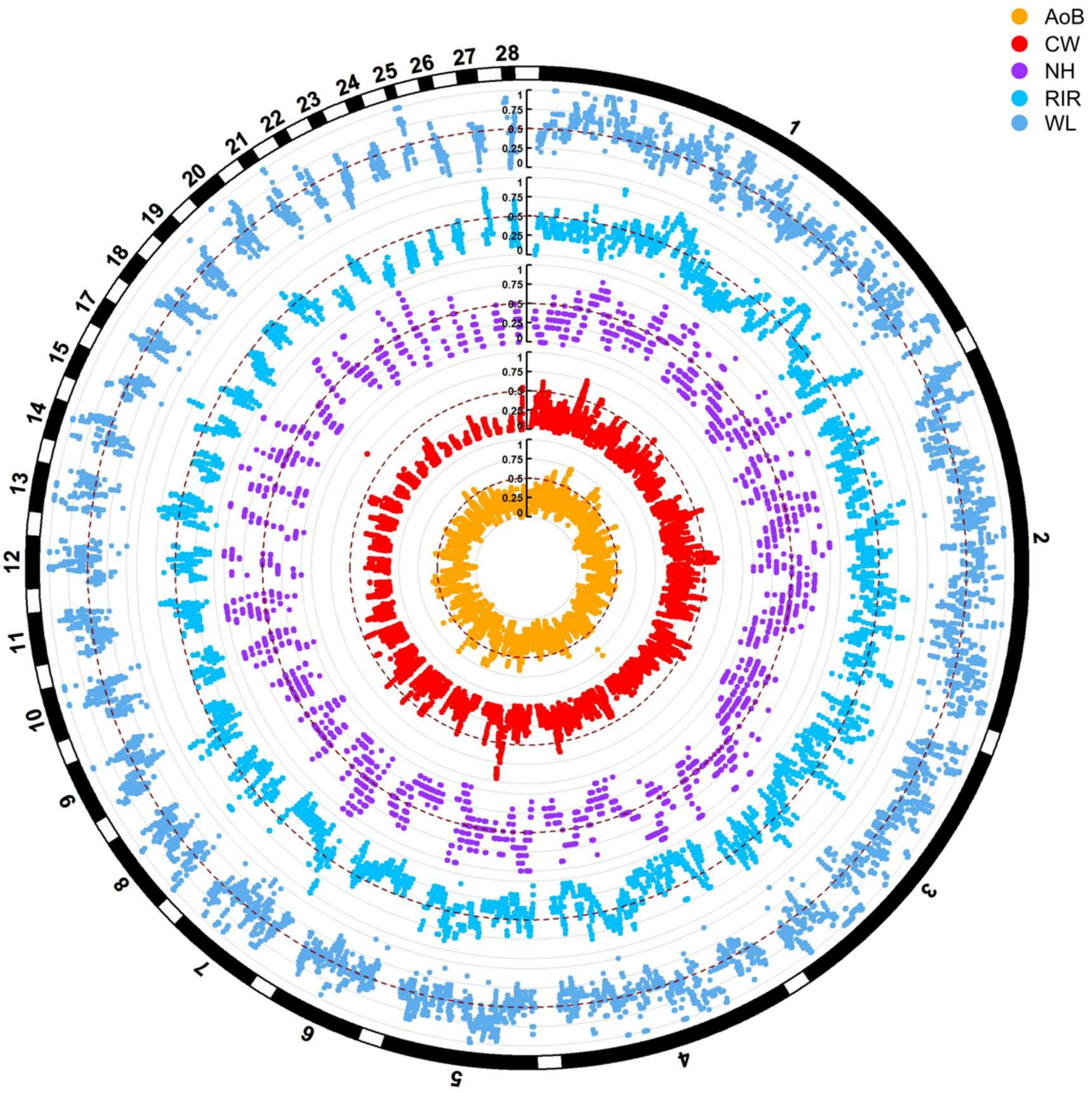

Supplementary Figure S3. Manhattan plots of the distribution of runs of homozygosity (ROH) islands in each chicken breed. These Manhattan plots for ROH distribution grouped by breed groups: (**A**) Old Russian indigenous breeds, (**B**) Improved Russian breeds, and (**C**) Specialized foreign breeds. The scale from 0 to 1 is the proportion of animals in which the corresponding SNP is detected; the red dotted line is the 50% threshold. Breed codes: AoB, Australorp Black; AS, Adler Silver; CW, Cornish White; KJ, Kuchino Jubilee; Kt, Kotlyarevsky; LMF, Leningrad Mille Fleur; NH, New Hampshire; OMF, Orloff Mille Fleur; PC, Poltava Clay; Pm, Pervomai; RBB, Russian Black Bearded; RC, Russian Crested; RIR, Rhode Island Red; RW1, Russian White (ARPRTI); RW2, Russian White (RRIFAGB); Ush, Ushanka; UshF, Ushanka Foot-feathered; WL, White Leghorn; YC, Yurlov Crower; ZS, Zagorsk Salmon.

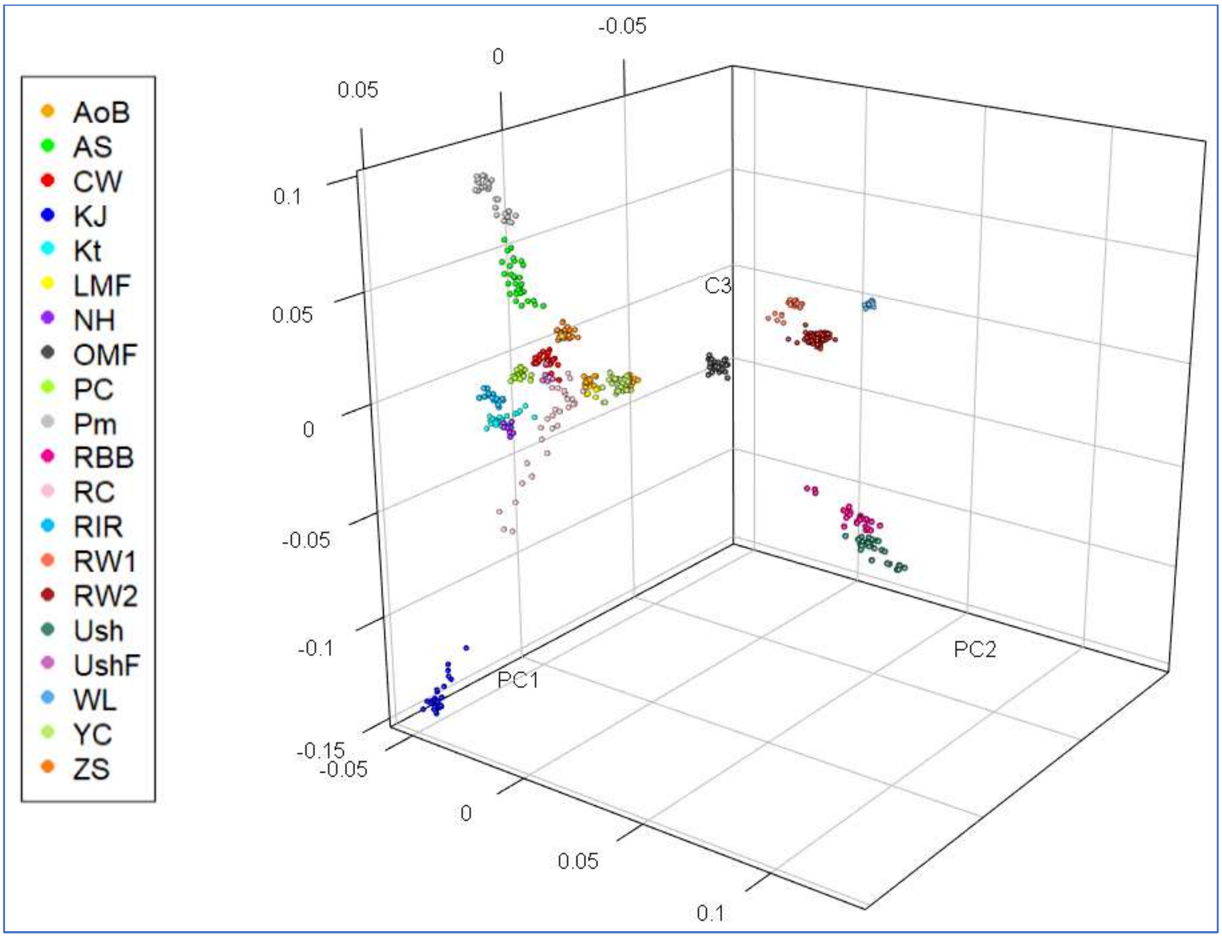

Supplementary Figure S4. 3-D PCA plot for the studied chicken breeds/populations based on genome-wide SNP genotypes. Distribution of breeds and individuals is shown in the dimensions of three coordinates, i.e., the first (PC1; *x*-axis), second (PC2; *y*-axis) and third (PC3; *z*-axis;) principal components (PCs). Breed codes: AoB, Australorp Black; AS, Adler Silver; CW, Cornish White; KJ, Kuchino Jubilee; Kt, Kotlyarevsky; LMF, Leningrad Mille Fleur; NH, New Hampshire; OMF, Orloff Mille Fleur; PC, Poltava Clay; Pm, Pervomai; RBB, Russian Black Bearded; RC, Russian Crested; RIR, Rhode Island Red; RW1, Russian White (ARPRTI); RW2, Russian White (RRIFAGB); Ush, Ushanka; UshF, Ushanka Foot-feathered; WL, White Leghorn; YC, Yurlov Crower; ZS, Zagorsk Salmon.

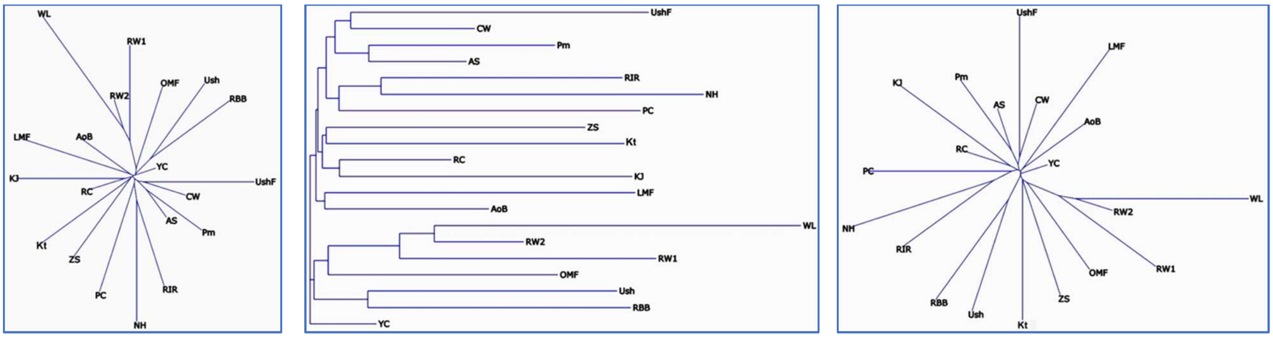

A B C

Supplementary Figure S5. Unrooted Neighbor Joining phylogenetic trees reconstructed for the 20 studied chicken breeds/populations based on the F_{ST} genetic distances. Radial (**A**) and hierarchical horizontal (**B**) trees using the Saitou and Nei [59] reconstruction method; and a radial Unweighted Neighbor Joining tree [63] (**C**). Breed codes: AoB, Australorp Black; AS, Adler Silver; CW, Cornish White; KJ, Kuchino Jubilee; Kt, Kotlyarevsky; LMF, Leningrad Mille Fleur; NH, New Hampshire; OMF, Orloff Mille Fleur; PC, Poltava Clay; Pm, Pervomai; RBB, Russian Black Bearded; RC, Russian Crested; RIR, Rhode Island Red; RW1, Russian White (ARPRTI); RW2, Russian White (RRIFAGB); Ush, Ushanka; UshF, Ushanka Foot-feathered; WL, White Leghorn; YC, Yurlov Crower; ZS, Zagorsk Salmon.

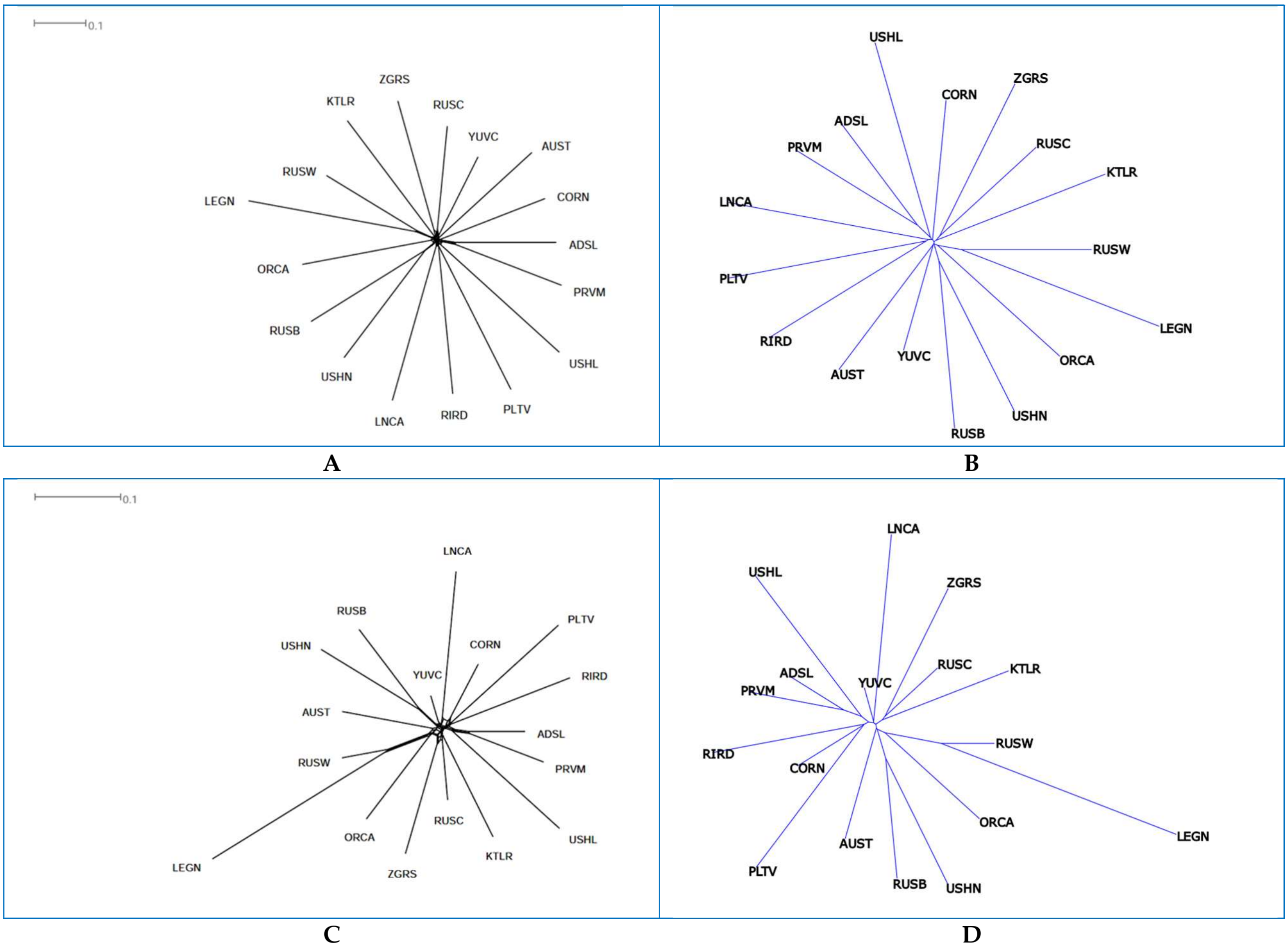

Supplementary Figure S6. Unrooted radial Neighbor Joining phylogenetic trees for 17 breeds (except KJ and NH) according to the results of SNP genotyping. These were based on the Reynolds et al. [61] genetic distance (**A**, **B**) and F_{ST} statistics [60] (**C**, **D**). Trees were reconstructed using the Neighbor-Net algorithm [57] (**A**, **C**) and the Neighbor Joining method [59] (**B**, **D**). Breed abbreviations: ADSL, Adler Silver; AUST, Australorp Black; CORN, Cornish White; KTLR, Kotlyarevsky; LEGN, White Leghorn; LNCA, Leningrad Mille Fleur; ORCA, Orloff Mille Fleur; PLTV, Poltava Clay; PRVM, Pervomai; RIRD, Rhode Island Red; RUSB, Russian Black Bearded; RUSC, Russian Crested; RUSW, Russian White; USHL, Ushanka Foot-feathered; USHN, Ushanka; YUVC, Yurlov Crower; ZGRS, Zagorsk Salmon.

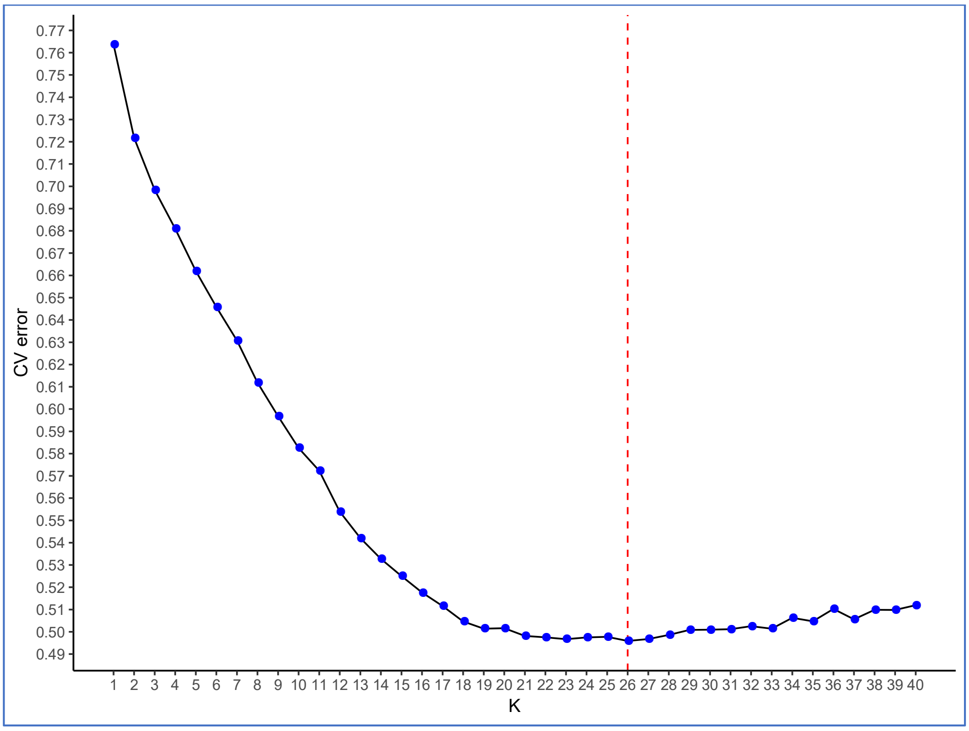

Supplementary Figure S7. Plot of cross-validation errors (CV error). This was built for different numbers of ancestral populations (K) using the Admixture program [65]. The minimum CV error was obtained at K = 26.

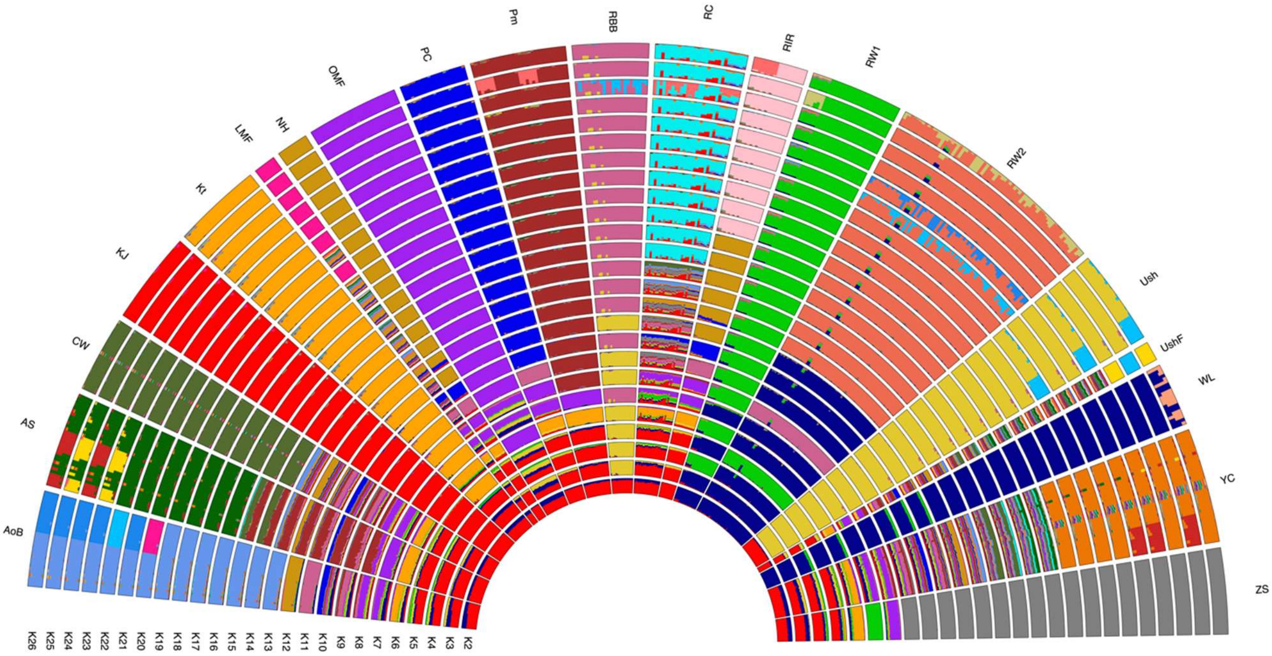

Supplementary Figure S8. Admixture-derived [65,66] structure of studied populations in the form of a semicircle. Each admixture diagram represents the cluster structure of the studied breed (population) at the given number K of clusters (at K = 2 to 26 ancestral populations). Breed codes: AoB, Australorp Black; AS, Adler Silver; CW, Cornish White; KJ, Kuchino Jubilee; Kt, Kotlyarevsky; LMF, Leningrad Mille Fleur; NH, New Hampshire; OMF, Orloff Mille Fleur; PC, Poltava Clay; Pm, Pervomai; RBB, Russian Black Bearded; RC, Russian Crested; RIR, Rhode Island Red; RW1, Russian White (ARPRTI); RW2, Russian White (RRIFAGB); Ush, Ushanka; UshF, Ushanka Foot-feathered; WL, White Leghorn; YC, Yurlov Crower; ZS, Zagorsk Salmon.
