# Supplementary material for: Whole Genome Screening Procures a Holistic Hold of the Russian Chicken Gene Pool Heritage and Demographic History
Source: Biology (Basel). 2023 Jul 10;12(7):979. doi: 10.3390/biology12070979 (PMC10376169; doi:10.3390/biology12070979)
Supplement: Supplementary file 1 [file biology-12-00979-s001.zip › Suppl Table S3 edited.pdf]

Supplementary Table S3: Pairwise Reynolds et al. [61] distances between the chicken breeds studied.

	AS	AoB	CW	Kt	WL	LMF	OMF	PC	Pm	RIR	RBB	RC	RW	UshF	Ush	YC	ZS
AS	*	0.490	0.449	0.524	0.621	0.562	0.507	0.558	0.425	0.532	0.536	0.450	0.489	0.549	0.535	0.400	0.517
AoB		*	0.478	0.545	0.622	0.579	0.523	0.584	0.517	0.560	0.547	0.469	0.489	0.582	0.545	0.426	0.538
CW			*	0.518	0.610	0.554	0.494	0.550	0.483	0.524	0.522	0.447	0.482	0.546	0.524	0.400	0.510
Kt				*	0.658	0.621	0.564	0.617	0.553	0.592	0.591	0.505	0.537	0.618	0.592	0.478	0.561
WL					*	0.694	0.631	0.704	0.639	0.691	0.666	0.608	0.541	0.707	0.671	0.567	0.648
LMF						*	0.597	0.639	0.587	0.611	0.621	0.557	0.574	0.645	0.619	0.518	0.612
OMF							*	0.599	0.531	0.577	0.553	0.494	0.505	0.595	0.549	0.444	0.552
PC								*	0.581	0.606	0.616	0.554	0.589	0.642	0.617	0.518	0.609
Pm									*	0.556	0.558	0.482	0.514	0.568	0.560	0.436	0.542
RIR										*	0.595	0.525	0.573	0.613	0.590	0.489	0.582
RBB											*	0.523	0.546	0.613	0.531	0.476	0.579
RC												*	0.471	0.554	0.522	0.390	0.478
RW													*	0.593	0.551	0.420	0.523
UshF														*	0.611	0.515	0.611
Ush															*	0.474	0.581
YC																*	0.468
ZS																	*

Note. Breed codes are given in Table 1.
